# Low‐Pressure CO_2_ and Aromatic Recognition by Interlayer Adaptive Crystal of π‐Hole‐Functionalized Zn(II) Coordination Sheets

**DOI:** 10.1002/anie.4090353

**Published:** 2026-08-24

**Authors:** Masahiro Abe, Tomoki Jitsukata, Shinpei Kusaka, Ryotaro Matsuda, Mikihiro Nomura, Akiko Hori

**Affiliations:** ^1^ Department of Applied Chemistry Graduate School of Engineering and Science Shibaura Institute of Technology Saitama Japan; ^2^ Department of Chemistry and Biotechnology School of Engineering and Department of Materials Chemistry Graduate School of Engineering Nagoya University Nagoya Aichi Japan; ^3^ Department of Applied Chemistry Graduate School of Engineering and Science Shibaura Institute of Technology Tokyo Japan

**Keywords:** adsorption, crystal‐to‐crystal, molecular recognition, porous material, zinc complex

## Abstract

A layer‐type host, an interlayer adaptive crystal (LAC) constructed from paddle‐wheel Zn(II) nodes, tetrafluoroisophthalate (*L*) linkers, and pyridine (Py) axial ligands, [Zn(*L*)Py]_2n_, exhibits guest‐induced, electrostatically driven breathing. The crystal contains 2.6 Å π‐hole‐defined ultramicropores that act not as classical channels but as electrostatic triggers for guest‐induced single‐crystal‐to‐single‐crystal expansion. Dynamic modulation of interlayer spacing and surface contacts yields exceptional flexibility and molecular selectivity. Tetrafluorophenyl π‐holes generate positive quadrupole fields that drive adsorption through host–host repulsion and strong recognition of guests with negative quadrupole moments. Uniaxial voids occupy 12% of the volume, allowing uptake of molecules larger than the ultramicropore. N_2_, CO_2_, CH_4_, and benzene show type‐I adsorption from very low pressure, incorporating 1.2, 2.0, 1.5, and 1.0 molecules per [Zn(*L*)Py]_2_ unit. CO_2_ uptake is accompanied by fully reversible interlayer expansion, while benzene inclusion expands the void space to 186% of that of the guest‐free crystal. CO_2_ is preferentially adsorbed over N_2_ and CH_4_, corroborated by recognition trends among benzene, trifluorobenzene, and hexafluorobenzene. Guest molecules form one‐dimensional arrays within the expanded lattice, and the breathing two‐dimensional sheet combines the uptake capability of metal‐organic frameworks (MOFs) with the molecular precision of nonporous adaptive crystals (NACs), demonstrating an interaction‐programmed mode of selective molecular inclusion.

## Graphical abstract

A perfluorinated Zn(II) coordination sheet operates as an interlayer adaptive crystal (LAC), where a small ultramicropore acts as an electrostatic trigger. Guest‐induced breathing driven by fluorinated π‐hole surfaces enables highly selective inclusion of CO_2_ over N_2_ and CH_4_, and benzene over other six‐membered hydrocarbons, beyond the intrinsic aperture, and promotes one‐dimensional guest ordering.

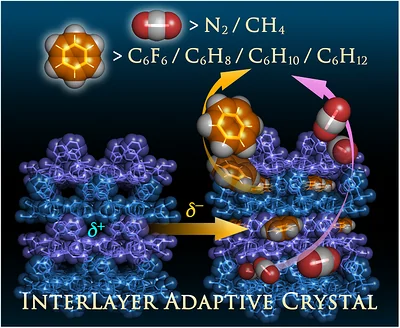

## 1 Introduction

Precise recognition of small molecules that share similar sizes, shapes, and physicochemical properties remains a fundamental challenge in molecular separation [1, 2, 3]. Conventional molecular‐sieving and physisorption‐based methods primarily rely on steric exclusion and adsorption energetics [4, 5, 6, 7], and therefore show limited efficiency when target molecules have similar dimensions and interaction characteristics. Accordingly, developing advanced separation strategies that transcend simple size exclusion is imperative. Such technologies are not only of fundamental interest but also directly support energy‐efficient chemical purification [8, 9, 10], CO_2_ capture and subsequent catalytic utilization [11, 12], environmental remediation [13, 14], and drug delivery and pharmaceutical production [15, 16], and have consequently attracted broad attention [17, 18, 19, 20]. In particular, the recognition of nonpolar molecules or small molecules lacking distinctive functional groups or strong interaction sites represents a key target, because these systems provide an opportunity to elucidate subtle intermolecular interactions and guide the rational design of next‐generation host materials, thereby linking industrial relevance with fundamental molecular science.

Porous adsorbents have emerged as central materials owing to their large accessible surface areas and permanent porosity. However, high surface area alone does not guarantee selectivity, especially when wide pore‐size distributions lead to nonspecific interactions. State‐of‐the‐art metal–organic frameworks (MOFs) [17, 18, 21, 22, 23, 24, 25] and covalent–organic frameworks (COFs) [26, 27, 28, 29, 30] mitigate this limitation through engineered pore environments and functional binding sites, enabling high‐performance sorbents for gas purification and fine chemical separations. Molecular crystals provide a complementary strategy in which molecular recognition can be coupled directly to guest‐induced lattice adaptation through precisely organized electrostatic surfaces and intermolecular contacts. Among molecular crystals, nonporous adaptive crystals (NACs) [31, 32, 33, 34, 35, 36, 37, 38, 39, 40, 41, 42, 43] exhibit guest‐induced structural changes and gated inclusion driven by host–guest interactions that outweigh host–host cohesion. However, NACs often suffer from stability or solubility challenges, and their structural transformations are frequently discontinuous and kinetically triggered, limiting applicability under mild adsorption conditions. Recent studies have demonstrated that molecular accommodation can also be enabled by local reorganization of densely fluorinated nanospaces or by conformational adaptation of flexible ligands in two‐dimensional MOFs [44, 45].

Here we introduce a distinct class of adaptive host, an interlayer adaptive crystal (LAC), whose selectivity is governed not by permanent pore architecture but by host‐guest interaction‐driven lattice breathing. In this system, a perfluorinated Zn(II) coordination sheet generates π‐hole‐rich surfaces that electrostatically repel each other, producing 2.6 Å ultramicropores that function not as transport channels but as electrostatic triggers for guest‐induced interlayer expansion. This mechanism differs from typical gate‐opening frameworks [46] in that the guest‐dependent structural response in the present system is discussed primarily in terms of electrostatic complementarity between fluorinated π‐hole surfaces and guest quadrupole distributions, rather than framework flexibility alone. This dynamic yet controllable adaptive behavior merges the molecular recognition precision of crystalline hosts with the adsorption efficiency of porous frameworks, offering a new design strategy for discriminating structurally similar molecules and expanding the scope of adaptive host materials. Here we elucidate molecular recognition of nonpolar molecules with notable quadrupole moments, such as N_2_, CO_2_, and benzene, and reveal guest alignment within fluorinated aromatic walls [28, 29, 47, 48]. In particular, at very low pressure, bellows‐like expansion and contraction of the interlayer space, mediated by tunable surface contacts, afford high guest uptake with fully reversible structural modulation.

## 2 Results and Discussion

### 2.1 Synthesis and Crystallographic Studies of **1**

Complex **1** was synthesized from Zn(OAc)_2_·2H_2_O, tetrafluoroisophthalic acid (H_2_ *L*), and pyridine (Py) in a 97% yield. Typically, a hexane solution (10 mL) of Py (0.80 mmol) was slowly added to a methanol solution (10 mL) containing H_2_ *L* (0.50 mmol) and Zn(OAc)_2_·2H_2_O (0.50 mmol), and the reaction mixture was kept at room temperature under liquid–liquid diffusion conditions. After several days, colorless prismatic single crystals (SCs) of [Zn(*L*)Py]_2n_ (**1**) were obtained, as shown in Figures 1a and S1. The result of elemental analysis shows good agreement for complex **1**: calcd. for C_26_H_10_F_8_N_2_O_8_Zn_2_·3H_2_O (%): C 38.31, H 1.98, N 3.44; found: C 38.61, H 1.79, N 3.68. Although elemental analysis indicates the presence of water molecules in the as‐grown crystals, the channel surface is lined with fluorinated aromatic rings and therefore provides a confined aromatic environment rather than a typical hydrophilic pore.

**FIGURE 1:**
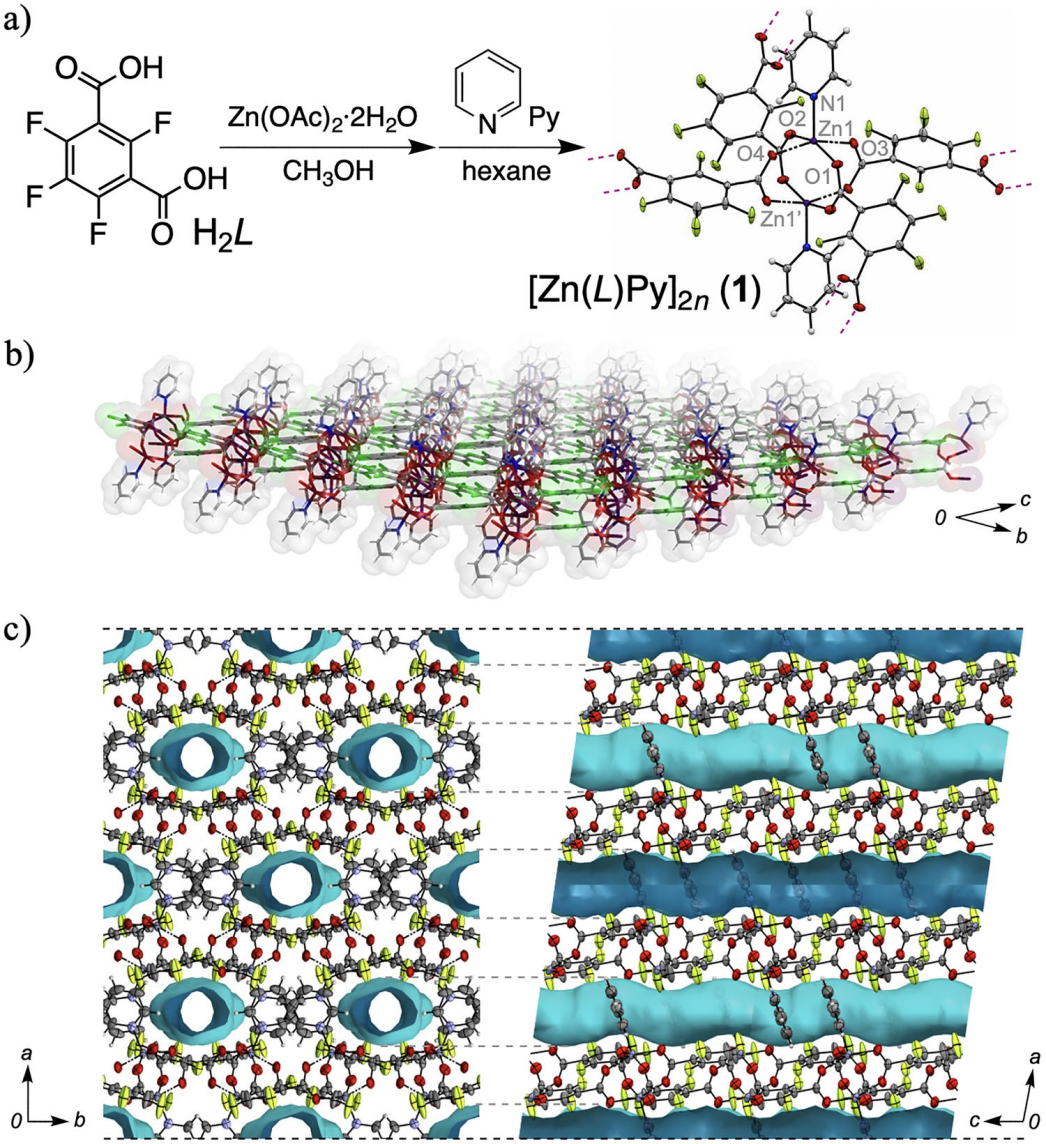
(a) Preparation and a part of the crystal structure, (b) a two‐dimensional sheet structure, and (c) packing structure at 100 K (CCDC2503165) with void spaces in blue of empty crystal of **1**; color schemes: C, gray; F, green; O, red; inside voids, dark blue; outside voids, blue.

SCs suitable for x‐ray diffraction analysis were obtained. The asymmetric unit consists of one Zn(II) ion, one *L* as a dianion and linker ligand, and one Py as an axial ligand, forming a dinuclear paddle‐wheel Zn_2_ core [49, 50]. These dinuclear units are interconnected by *L* ligands to generate a two‐dimensional sheet structure extending along the *bc* plane (Figures 1b, S2–S10, and Tables S1–S4). In this two‐dimensional sheet, grooves aligned along the 011 plane are formed by the exposed surfaces of the tetrafluorophenylene moieties of *L*. The axial Py coordinated to the Zn(II) centers protrude above and below the sheet. As a result, the fluorine‐lined grooves (highlighted in green in the figure) create electron‐deficient π‐hole surfaces with unique electronic characteristics. These sheets stack along the *a*‐axis through weak π–π interactions between the protruding Py moieties, giving a three‐dimensional packing. However, the partial positive charge on the Py hydrogens and the electropositive nature of the π‐hole regions prevent close interdigitation of the sheets, so that the layers adopt an offset stacking mode. As a consequence, small “ultramicropores” are created between the sheets that penetrate through the lattice along the *c* axis, as illustrated in Figure 1c, giving a calculated porosity of 12.0% (probe radius = 1.20 Å with grid = 0.3 Å). The mean internal diameter of these one‐dimensional channels is approximately 2.6 Å (estimated by assuming a rigid cylindrical channel defined by the van der Waals radii (vdW) of the host framework). This dimension is slightly smaller than the kinetic diameters required for the diffusion of small molecules such as water or nitrogen. Consequently, a continuous one‐dimensional channel is observed for a hydrogen‐sized probe (1.20 Å; void = 339 Å^3^), whereas the pathway becomes segmented for N,O‐sized probes (1.55 Å; void = 178 Å^3^, 6.3%) and is completely closed for a carbon‐sized probe (1.70 Å; void = 0.0 Å^3^, 0.0%) (Figure S11).

Consistently, the final difference Fourier map exhibited no significant residual electron density, with the highest peak of 0.679 e Å^−3^ and the deepest hole of −0.446 e Å^−3^, supporting the absence of guest molecules in the crystal lattice. Along the *a*‐axis, the π‐hole planes protrude in such a way that vertical gaps form, as if repelled by neighboring π‐hole regions. Laterally, the Py acts as flexible pillars that support the framework structure. This crystal maintains its ultramicropore architecture even under vacuum or mechanical stress such as grinding, as evidenced by variable‐condition powder x‐ray diffraction (pXRD) measurements and thermogravimetric (TG) studies (Figures S12 and S13). Furthermore, variable‐temperature structural analysis revealed that the *a*‐axis tends to expand with increasing temperature, while the *b*‐ and *c*‐axes remain largely unchanged, indicating robust two‐dimensional structural integrity.

### 2.2 Guest Adaptive Adsorption of **1**

Gas adsorption/desorption behaviors of **1** were investigated using microcrystalline powder samples activated by heating under vacuum at 100°C for 3 h (Figures 2, S15, and Tables S6–S9). The molecular volumes *V* shown in the figure represent vdW volumes, whereas *d* denotes the kinetic diameter relevant to molecular diffusion.

**FIGURE 2:**
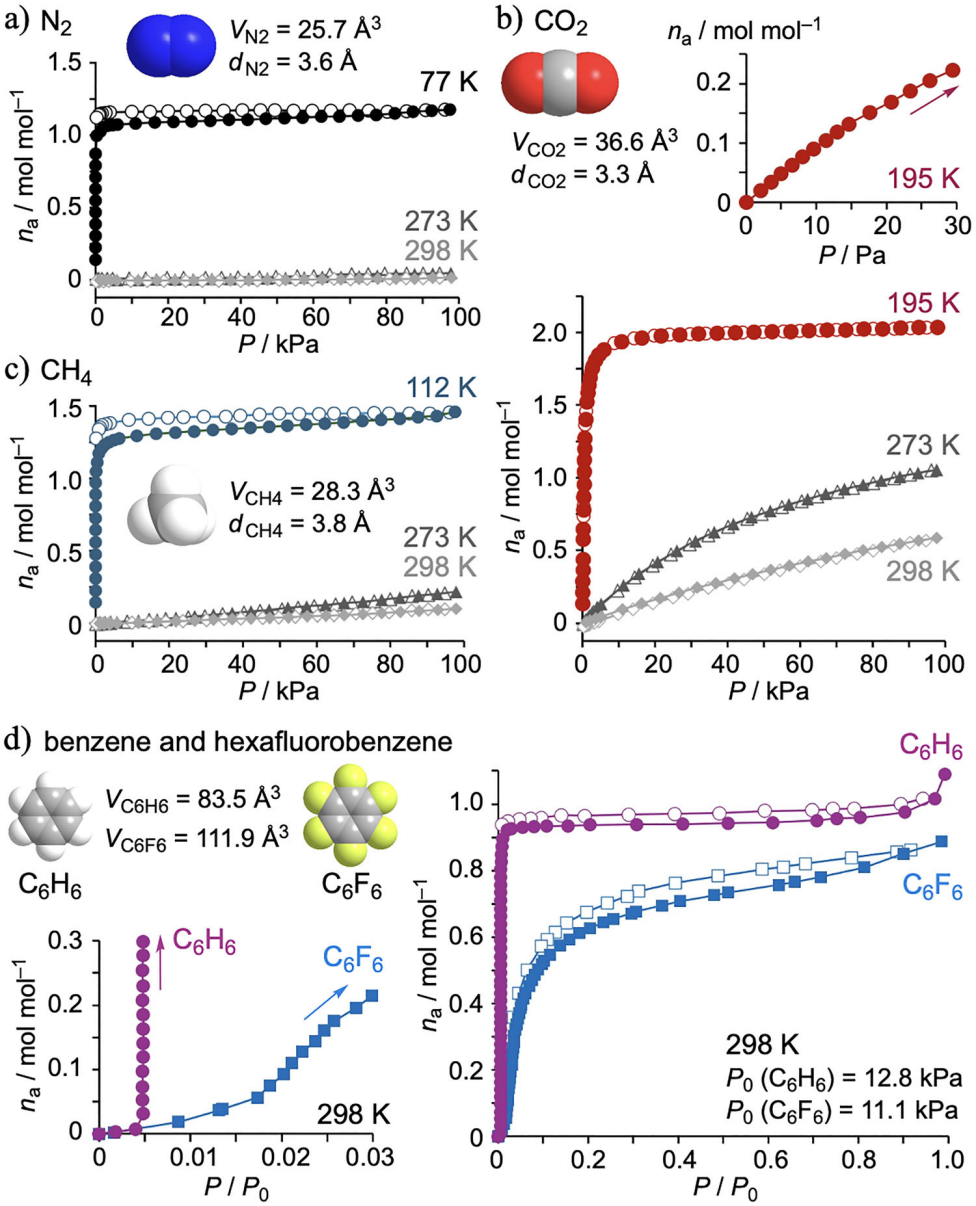
Adsorption and desorption isotherms of **1** for (a) N_2_ at 77, 273, and 298 K, (b) CO_2_ at 195, 273, and 298 K, (c) CH_4_ at 112, 273, and 298 K, and (d) benzene (C_6_H_6_) and hexafluorobenzene (C_6_F_6_) vapors at 298 K. The upper panel in (b) shows the CO_2_ adsorption behavior in the ultra‐low‐pressure region at 195 K, and the lower‐left panel in (d) shows an expanded view of the low‐relative‐pressure region. Filled and open symbols represent the adsorption and desorption branches, respectively. The adsorbed amounts, *n*_a_, are expressed per [Zn(*L*)Py]_2_ unit. The molecular volumes, *V*, are vdW volumes, whereas *d* represents the kinetic diameter.

The N_2_ adsorption isotherm exhibited a type‐I profile at 77 K, with a sharp increase in uptake in the low‐pressure region, indicating microporosity (Figure 2a). The adsorption rapidly reached approximately 1.0 mol mol^−1^ of N_2_ per [Zn(*L*)Py]_2_ unit below 0.28 kPa. The uptake then plateaued, reaching 1.2 mol mol^−1^ at 97.5 kPa. A pronounced hysteresis was observed during desorption, with 1.1 mol mol^−1^ of N_2_ remaining at 0.23 kPa. In contrast, the N_2_ uptakes at 273 and 298 K were only 0.049 and 0.017 mol mol^−1^ near 98 kPa, respectively. These results indicate that **1** accommodates N_2_ efficiently at 77 K, whereas its affinity for N_2_ is weak near ambient temperature. The adsorption profiles were reproducible over repeated measurements, supporting the intrinsic nature of the observed adsorption behavior.

The CO_2_ adsorption behavior and its temperature dependence were particularly noteworthy (Figure 2b). Despite the kinetic diameter of CO_2_ (3.3 Å) being larger than the estimated pore size of the ultramicropores, the adsorption isotherm followed a type‐I profile, and rapid uptake into the crystal lattice was observed. At 195 K, the uptake reached approximately 1.5 mol mol^−1^ at 1 kPa and approached a plateau of 2.0 mol mol^−1^ at 98 kPa. The adsorption and desorption branches almost overlapped, demonstrating reversible CO_2_ uptake. A separate measurement focused on the ultra‐low‐pressure region revealed appreciable uptake at pressures as low as 2.0 Pa. As shown in the upper inset of Figure 2b, the framework accommodated approximately 0.2 mol mol^−1^ of CO_2_ at only 26 Pa. The adsorption uptake corresponded to approximately two CO_2_ molecules per [Zn(*L*)Py]_2_ unit, suggesting that CO_2_ is accommodated at a defined number of adsorption sites within the adaptive interlayer space. Because this uptake is accompanied by reversible expansion of the lattice along the *a*‐axis, as demonstrated by the temperature‐dependent pXRD measurements described below, CO_2_ accommodation cannot be attributed solely to filling of the static ultramicropore but involves adaptive expansion of the interlayer space. The CO_2_ uptake decreased systematically with increasing temperature but remained substantial near ambient conditions. At approximately 98 kPa, the uptakes were 2.0 mol mol^−1^ at 195 K, 1.1 mol mol^−1^ at 273 K, and 0.58 mol mol^−1^ at 298 K. The considerably higher uptake of CO_2_ than N_2_ at 273 and 298 K demonstrates the preferential affinity of the fluorinated interlayer environment for CO_2_.

To further evaluate this molecular preference, CH_4_ adsorption was examined at 112, 273, and 298 K (Figure 2c). At 112 K, CH_4_ exhibited a type‐I profile, reaching 1.2 mol mol^−1^ at 0.89 kPa and gradually increasing to 1.5 mol mol^−1^ at 97.5 kPa. In contrast, the uptake at 98 kPa decreased to 0.22 mol mol^−1^ at 273 K and 0.10 mol mol^−1^ at 298 K. Thus, CH_4_ can be accommodated within the interlayer space at cryogenic temperatures, but its affinity near ambient temperature is considerably weaker than that of CO_2_.

Taken together, the temperature‐dependent isotherms reveal a clear preference for CO_2_ over both N_2_ and CH_4_ near ambient temperature. This adsorption hierarchy cannot be explained solely by molecular size because the kinetic diameters of N_2_, CO_2_, and CH_4_ are 3.6, 3.3, and 3.8 Å, respectively. Instead, the particularly strong CO_2_ affinity, including appreciable adsorption at very low pressure, reflects favorable interactions between CO_2_ and the fluorinated adaptive interlayer environment. These single‐component adsorption results provide the thermodynamic basis for the subsequent dynamic separation of CO_2_/N_2_ and CO_2_/CH_4_ mixtures.

The uniqueness of the interlayer characteristics of this crystal, namely the guest‐adaptive expansion and the structural flexibility of the two‐dimensional sheets, was revealed by remarkable transformations. Figure 2d presents the vapor adsorption isotherms of benzene (C_6_H_6_) and hexafluorobenzene (C_6_F_6_), showing that molecules larger than the ultramicropores depicted in Figure 1c can nevertheless be incorporated. Notably, at 298 K, the adsorption of C_6_H_6_ by **1** increased sharply at about 5 × 10^−3^ *P/P*_0_ and nearly reached saturation at ∼0.01 *P/P*_0_. Such behavior, rarely observed in NACs, indicates an exceptionally high affinity, which can reasonably be ascribed to the assisting role of the ultramicropores. Although the total pore volume of the empty lattice (*V* = 339 Å^3^) and the volume of the adsorbed benzene corresponding to four incorporated molecules (*V*_C6H6_ = 83.5 × 4 = 334 Å^3^) appear nearly identical, the actual diameter of benzene (*d* = 5.9 Å) is about twice as large as the diameter of the empty ultramicropore (*d* = 2.6 Å). Interestingly, C_6_F_6_, despite its similar shape and physical properties, was also incorporated into the lattice but required a higher relative pressure threshold and exhibited a lower overall capacity compared to benzene. At 0.01 *P/P*_0_, the uptake was 0.90 mol mol^−1^ for C_6_H_6_ but only 0.03 mol mol^−1^ for C_6_F_6_. At 0.02 *P/P*_0_, the respective values were 0.92 and 0.09 mol mol^−1^, highlighting a distinct difference in low‐pressure inclusion behavior. At 0.9 *P/P*_0_, the final uptake amounts were 0.98 mol mol^−1^ for C_6_H_6_ and 0.85 mol mol^−1^ for C_6_F_6_.

The molecular recognition properties of the nearly nonporous two‐dimensional sheet **1**, as predicted from nitrogen adsorption, follow simple integer ratios relative to the [Zn(*L*)Py]_2n_ units. The steep increase in uptake is rationalized by a micropore filling associated with the ultramicropores, whereas the selective accommodation of molecules of different sizes, from CO_2_ to benzene, arises from correlated expansion of the two‐dimensional sheets. Although possible contributions from defect sites or local linker motion cannot be completely excluded, the crystallographic and pXRD data indicate that the uptake occurs predominantly within the framework structure. The fact that **1** shows a stronger affinity for CO_2_ gas and benzene vapor than for N_2_ gas is particularly noteworthy. The π‐hole surfaces formed by the tetrafluoroaromatic walls, such as C_6_F_4_(COOH)_2_ (electrostatic potential (ESP) at the ring center = +83 kJ mol^−1^) and the corresponding C_6_H_2_F_4_ (ESP = +37 kJ mol^−1^, zz component of the quadrupole moment (*Q*_zz_) = +11 × 10^−40^ C·m^2^, Figure S14 and Table S5) [51], constitute the ultramicropores and are capable of stabilizing molecules with opposite quadrupole distributions, such as CO_2_ (ESP = −67 kJ mol^−1^, *Q*_zz_ = −14 × 10^−40^ C·m^2^) [52] and benzene (ESP at the ring center = −94 kJ mol^−1^, *Q*_zz_ = −29 × 10^−40^ C·m^2^) [53, 54].

In this system, fluorination plays multiple roles beyond simple lattice construction. The fluorinated aromatic rings generate electron‐deficient π‐hole surfaces that favor guests with negative quadrupole moments, while electrostatic repulsion between the layers creates adaptive interlayer voids for guest inclusion. In addition, the fluorinated rings directly participate in host–guest stabilization after inclusion. Thus, the guest selectivity of **1** is governed primarily by electrostatic complementarity on the π‐hole surface rather than by a simple hydrophilic/hydrophobic effect. The *Q*_st_ analysis (Figures S16 and S17) supports a relatively strong host–guest electrostatic interaction in the low‐uptake region at low temperature, consistent with preferential stabilization of CO_2_ at the fluorinated interlayer surface. Although this analysis does not exclude the contribution of confinement, it supports the view that electrostatic host–guest interactions play an important role in the initial uptake process. These findings prompted further investigation into the structural features of CO_2_ inclusion and its separation behavior, as well as the uptake and discrimination of aromatic molecules.

### 2.3 Structural and Separation Studies of CO_2_

pXRD measurements clearly revealed a guest‐induced structural change associated with CO_2_ inclusion (Figures 3, S18, and S19). The diffraction pattern of the activated framework after evacuation under vacuum (Figure 3b) was in good agreement with that of the empty phase of **1** obtained from SC‐XRD simulation (Figure 3a). Upon introduction of CO_2_ at 273 K, a characteristic 200 reflection shifted, indicating the formation of a CO_2_‐loaded phase in which the lattice expanded by 0.3 Å along the *a*‐axis. This structural change was fully reversible, and no amorphization was observed, demonstrating the robustness of the crystal lattice. When the temperature was lowered from 273 to 195 K, a further expansion of 0.2 Å along the *a*‐axis (with contraction along the *b*‐ and *c*‐axes) was observed. This additional expansion is consistent with increased CO_2_ incorporation at lower temperatures and with the interlayer expansion mechanism inferred from the adsorption data. The adsorption uptake approaches two CO_2_ molecules per [Zn(*L*)Py]_2_ unit under these conditions. This uniaxial flexibility highlights the adaptability of the two‐dimensional sheet toward CO_2_ accommodation. Although lattice expansion upon cooling may superficially resemble negative thermal expansion (NTE) behavior reported for some MOFs, the present response is accompanied by additional CO_2_ uptake and is therefore not interpreted as a simple thermal response.

**FIGURE 3:**
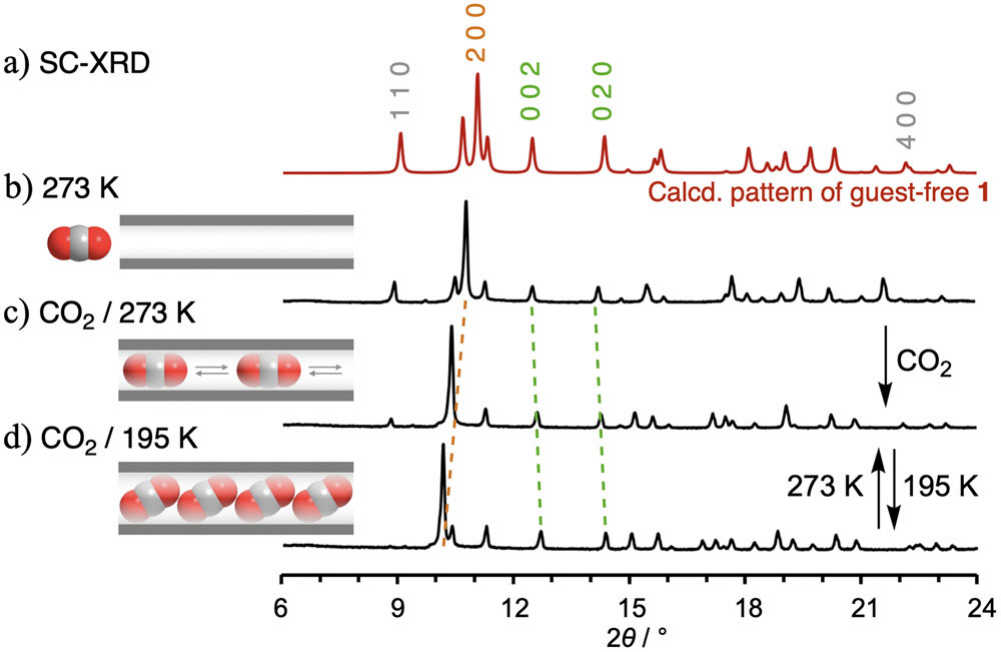
Temperature‐dependent pXRD patterns (Cu*K*α, *λ* = 1.54 Å) showing the structural response of **1** to CO_2_: (a) pattern calculated from the SC‐XRD structure of guest‐free **1** at 100 K, (b) activated **1** under vacuum at 273 K, (c) **1** under CO_2_ at 273 K, and (d) **1** under CO_2_ at 195 K. Dashed lines indicate the shifts of selected reflections.

To evaluate the CO_2_ separation performance of **1**, breakthrough measurements were performed at 303 K using equimolar CO_2_/N_2_ and CO_2_/CH_4_ mixtures under dry and humid conditions [42, 43, 55]. For the CO_2_/N_2_ mixture under dry conditions, N_2_ appeared at the outlet substantially earlier than CO_2_ (Figures 4a, S21, and S22). The normalized outlet signal reached *I*/*I*_0_ = 0.5 at 151 s for N_2_, whereas the corresponding value for CO_2_ was reached at 211 s, giving a breakthrough delay of approximately 60 s. Integration of the breakthrough curves relative to the blank measurement gave estimated dynamic uptake amounts of approximately 0.05 mol mol^−1^ for N_2_ and 0.24 mol mol^−1^ for CO_2_. The substantially delayed breakthrough and approximately fourfold greater uptake of CO_2_ clearly demonstrate selective CO_2_ capture over N_2_ by **1**. A similar but distinct separation behavior was observed for the CO_2_/CH_4_ mixture (Figure 4b). Under dry conditions, CH_4_ reached *I*/*I*_0_ = 0.5 at 148 s, followed by CO_2_ at 200 s, corresponding to a delay of approximately 52 s. The estimated dynamic uptake amounts were 0.06 mol mol^−1^ for CH_4_ and 0.20 mol mol^−1^ for CO_2_. Thus, although CH_4_ was measurably retained in the column, CO_2_ showed a markedly longer retention time and an approximately threefold greater uptake. This result confirms that the dynamic separation is not limited to CO_2_/N_2_ discrimination but also operates for the more challenging CO_2_/CH_4_ combination. Taken together, both experiments show that N_2_ and CH_4_ pass through the column earlier, whereas CO_2_ is selectively retained and elutes more gradually. This common behavior toward two chemically different competing gases is consistent with the single‐component adsorption isotherms at 298 K, which show a stronger affinity of **1** for CO_2_ than for either N_2_ or CH_4_. The observed preference is consistent with favorable quadrupolar complementarity between CO_2_ and the positively polarized fluorinated interlayer surface [56], rather than a general affinity toward nonpolar molecules or separation governed solely by molecular size and transport properties.

**FIGURE 4:**
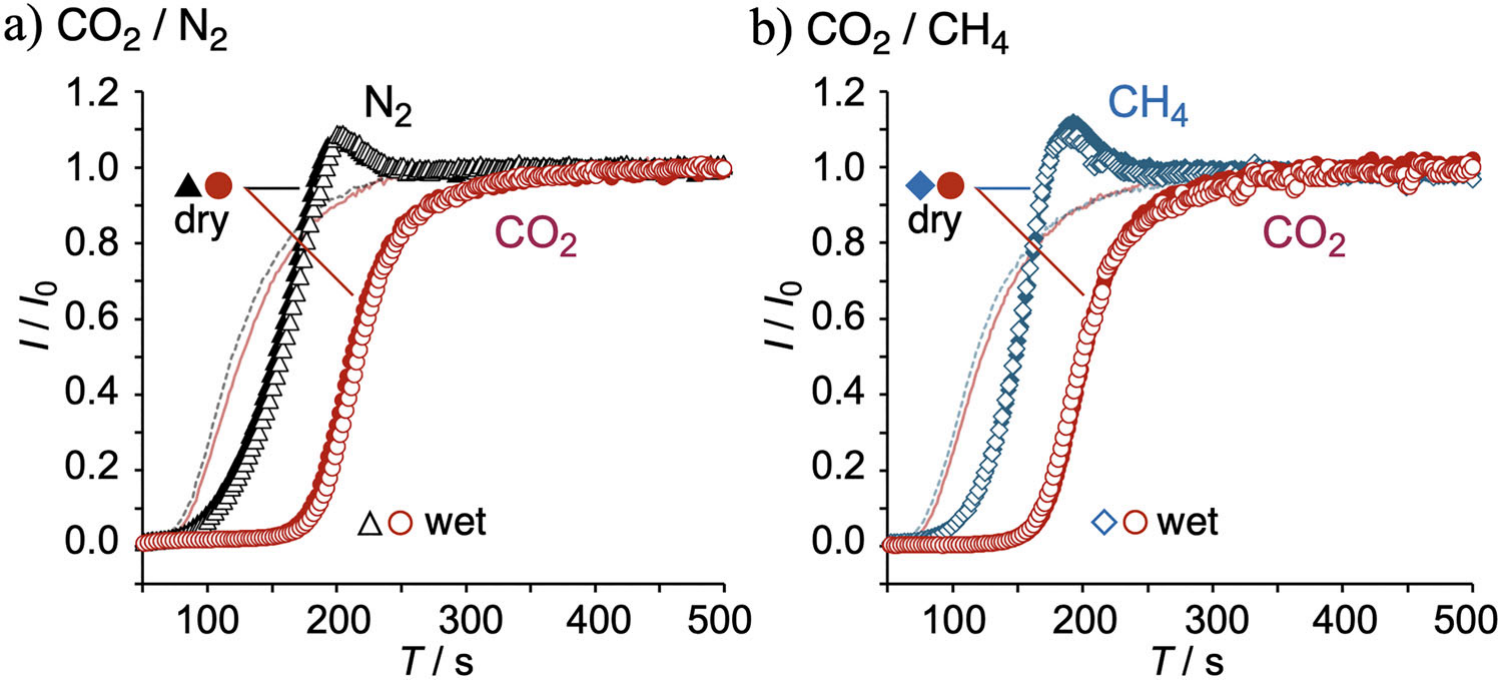
Breakthrough curves for equimolar (a) CO_2_/N_2_ and (b) CO_2_/CH_4_ mixtures at 303 K under dry and humid conditions using **1**. Filled and open symbols represent measurements under dry and humid conditions, respectively, and gray and blue dashed lines show the corresponding blank measurements of N_2_ and CH_4_, respectively, while the red solid lines show the corresponding CO_2_ blank measurements. The humidified feed had a relative water‐vapor pressure of *P*/*P*_0_ = 0.89. The outlet signals are expressed as normalized mass‐spectrometric intensities, *I*/*I*_0_. Color scheme: red, CO_2_; black, N_2_; blue, CH_4_.

Remarkably, the CO_2_ separation behavior was preserved under highly humid conditions at *P*/*P*_0_ = 0.89. For the CO_2_/N_2_ mixture, the 50% breakthrough times were 157 s for N_2_ and 216 s for CO_2_, maintaining a delay of approximately 59 s. The estimated CO_2_ uptake was 0.25 mol mol^−1^, essentially identical to the value of 0.24 mol mol^−1^ obtained under dry conditions. Similarly, for the CO_2_/CH_4_ mixture, CH_4_ and CO_2_ reached *I*/*I*_0_ = 0.5 at 147 and 199 s, respectively, retaining the same breakthrough delay of approximately 52 s observed under dry conditions. The estimated CO_2_ uptake also remained nearly constant at 0.21 mol mol^−1^, compared with 0.20 mol mol^−1^ under dry conditions. Thus, even at high water‐vapor pressure, neither the CO_2_ breakthrough delay nor its dynamic uptake was appreciably diminished. This humidity‐tolerant selectivity highlights the distinctive CO_2_‐recognition capability of the fluorinated adaptive interlayer space, even in the presence of competing water vapor.

### 2.4 Structural and Separation Studies of Aromatic Guests

The influence of fluorination on host recognition was next examined using aromatic molecules. Vapor adsorption experiments revealed that compound **1** adsorbs aromatic guests, including both benzene (C_6_H_6_) and hexafluorobenzene (C_6_F_6_), with C_6_H_6_ being preferentially taken up. Because these aromatic guests are substantially larger than the intrinsic ultramicropores of **1**, SC‐XRD analyses of the guest‐included crystals were performed to clarify how the interlayer structure accommodates them. Vapors of C_6_H_6_ (ESP at the ring center = −94 kJ mol^−1^, *Q*_zz_ = −29 × 10^−40^ C·m^2^), trifluorobenzene (C_6_H_3_F_3_; ESP = +6.0 kJ mol^−1^, *Q*_zz_ = −1.1 × 10^−40^ C·m^2^) [51], and C_6_F_6_ (ESP = +98 kJ mol^−1^, *Q*_zz_ = +34 × 10^−40^ C·m^2^) were reversibly incorporated into both single crystals and microcrystalline powders of **1**. In particular, single‐crystal experiments revealed a complete single crystal‐to‐single crystal (SC‐to‐SC) phase transition, in which guest molecules fully filled the [Zn(*L*)Py]_2_ unit simply by immersing an empty crystal in the corresponding solvent for approximately 5 s. Table 1 summarizes the unit‐cell changes, and Figure 5 displays the structures of the guest‐loaded crystals. Upon benzene inclusion, the space group remained *C*2/*c*, and a pronounced expansion along the *a*‐axis was observed (*a* = 16.11 Å for **1** and *a′* = 18.94 Å for **1**•C_6_H_6_). This transformation proceeded while retaining crystallinity, and four benzene molecules per unit cell (equivalent to one molecule per [Zn(*L*)Py]_2_ unit) were incorporated (Figure 5a). The benzene guests were sandwiched between the tetrafluorophenyl rings of ligand *L* (Figure 5b). The centroid(*C*_g_)‐to‐centroid(*C*_g_) distance between the benzene ring of guest and the fluorinated phenylene ring of host [*C*_g_(C_6_H_6_)⋯*C*_g_(C_6_F_4_)] was 3.658 Å, with a perpendicular offset of 3.633 Å. This molecular recognition is primarily driven by π‐hole⋯π‐interactions [34, 35, 57, 58], in which the electron‐deficient region (positive electrostatic potential) of the fluorinated aromatic ring electrostatically interacts with the electron‐rich π‐region of benzene (negative electrostatic potential). These interactions play a key role in stabilizing the guest molecules within the host lattice. Similarly, C_6_H_3_F_3_ and C_6_F_6_ were fully incorporated into the [Zn(*L*)Py]_2_ unit of **1** by immersing empty crystals in the corresponding solvents, further demonstrating the flexibility of the two‐dimensional sheet structure of **1**.

**TABLE 1:** Parts of cell parameters of **1**, **1**•C_6_H_6_, **1**•C_6_H_3_F_3_, and **1**•C_6_F_6_ with expansion rate of *a*‐axis in the crystals; all crystals are isomorphous: Monoclinic C2/c.

	1	1•C_6_H_6_	1•C_6_H_3_F_3_	1•C_6_F_6_
*a*/Å	16.1114(8)	18.943(7)	19.14(1)	17.845(2)
*b*/Å	12.3499(6)	12.457(5)	12.566(6)	12.7339(15)
*c*/Å	14.2728(7)	13.595(5)	13.780(7)	14.0076(16)
*β*/°	95.859(2)	102.822(10)	104.269(14)	100.076(3)
*V*/Å^3^	2825.1(2)	3128(2)	3212(3)	3133.9(6)
Void space^a^	339 Å^3^	629 Å^3^	662 Å^3^	608 Å^3^
Expansion rate^b^	100%	186%	195%	179%
CCDC no.	2503165	2503166	2503167	2503168

^a^ Void spaces were calculated using probe radius = 1.20 Å with grid = 0.3 Å.

^b^ Expansion rate (%) was calculated as void space (with guest) / void space (guest free) × 100.

**FIGURE 5:**
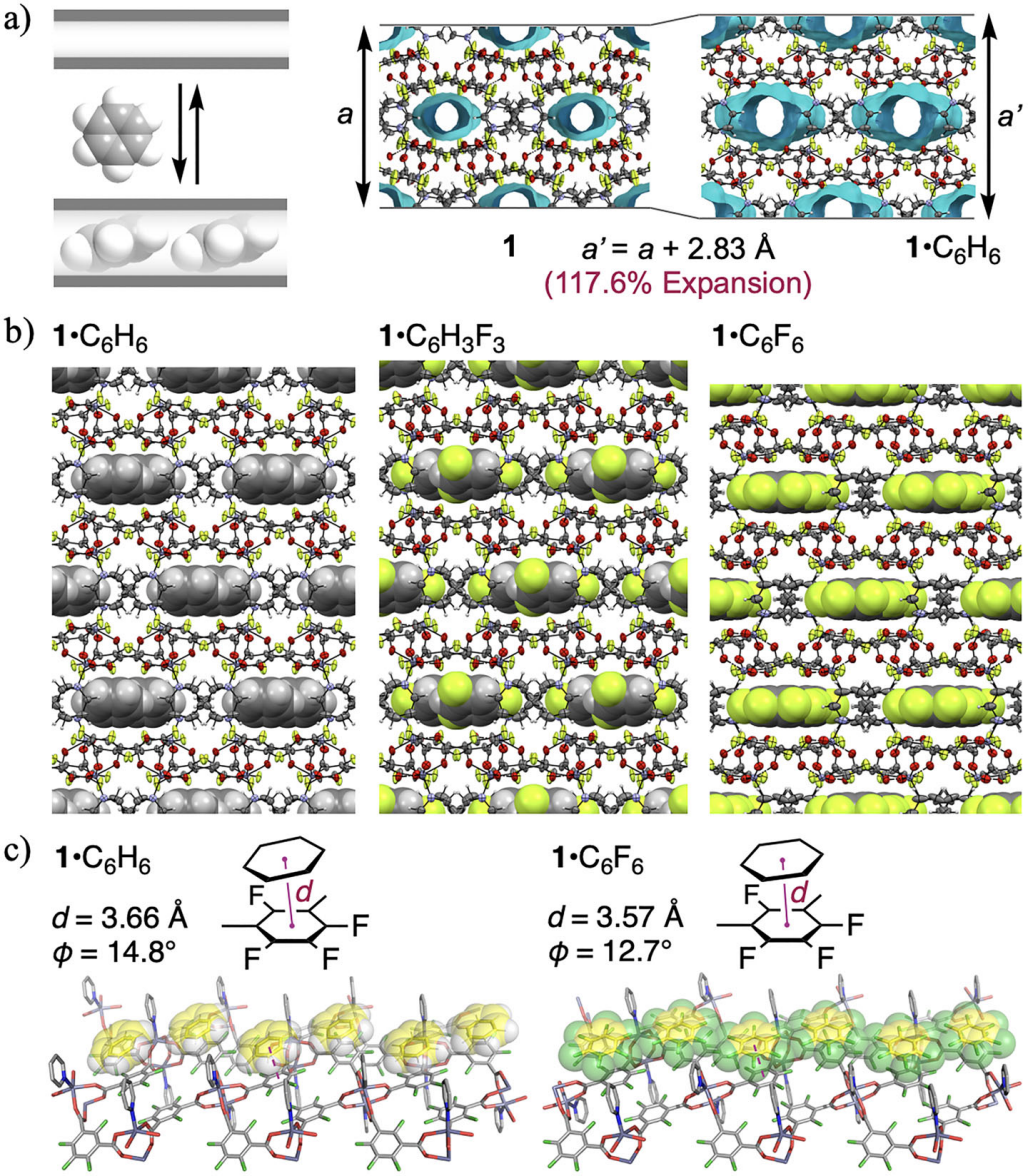
(a) Schematic illustrations and void spaces of interlayer benzene‐adaptive crystal **1** and **1**•C_6_H_6_ (benzene omitted; blue regions indicate void spaces). (b) Partial packing structures of **1**•C_6_H_6_, **1**•C_6_H_3_F_3_, and **1**•C_6_F_6_. (c) Guest arrangements in **1**•C_6_H_6_ and **1**•C_6_F_6_, showing a zigzag arrangement for benzene and a planar alignment for hexafluorobenzene. ORTEP views are drawn with 50% thermal ellipsoids; color scheme: C, gray (host) and yellow (guest); N, blue; F, green; O, red; Zn, dark gray.

The driving force for guest insertion arises from a combination of electrostatic and dispersion interactions. Among these contributions, π‐hole⋯π interactions are expected to play an important role because of the complementary charge distributions of the host and guest molecules. However, it is well known that π⋯π interactions between π‐electron systems and π‐hole⋯π‐hole interactions between electron‐deficient rings can also contribute to the insertion process, unlike classical ionic columns. Interestingly, a distinctive trend was observed in the expansion along the *a*‐axis: 19.14 Å for **1**•C_6_H_3_F_3_ and 17.84 Å for **1**•C_6_F_6_, the latter being unexpectedly shorter relative to benzene despite its larger size (Figure 5b). This behavior is associated with the planar alignment of C_6_F_6_ molecules through F⋯F contacts, whereas the other two aromatic guests adopt oblique arrangements. Although hydrogen atoms are generally *δ* ^+^ and repulsive, fluorine atoms possess both *δ* ^–^ lone‐pair regions (sp^3^‐hybridized) and adjacent *δ* ^+^ σ‐hole regions [59, 60], enabling F⋯F interactions when complementary positions are adopted [61, 62, 63, 64, 65, 66]. The charge separation on fluorine is relatively small in the context of halogen bonding, and although F⋯F contacts are sometimes observed in crystal packing, they are often influenced by the overall packing environment of the crystal. Therefore, cases in which H⋯H interactions (as in the C_6_H_6_ arrangement in **1**•C_6_H_6_) and F⋯F interactions (as in the C_6_F_6_ arrangement in **1**•C_6_F_6_) can be compared this clearly are exceedingly rare. The corresponding host–guest contact distances were 3.680 Å for [*C*_g_(C_6_H_3_F_3_)···*C*_g_(C_6_F_4_)], with a vertical offset of 3.662 Å, and 3.567 Å for [*C*_g_(C_6_F_6_)···*C*_g_(C_6_F_4_)], with a vertical offset of 3.350 Å. These interactions stabilize the guest arrangement. Thus, this LAC enables the one‐dimensional alignment of guest molecules inside the crystal without disrupting intermolecular interactions among the guests, a unique characteristic of this material.

To investigate the selectivity of molecular recognition within this void, single crystals were immersed in a mixture of guests, and their separation performance was examined. As shown in Figure 6a, briefly immersing the empty single crystals of **1** in an equimolar mixture of C_6_H_6_ and C_6_F_6_ resulted in selective inclusion of C_6_H_6_, as confirmed by SC‐XRD analysis, TG analysis (Figure 6b), and pXRD (Figures 6c and S23). The guest desorption amount determined from TG was 9.0% up to 110°C, matching the theoretical C_6_H_6_ weight fraction in **1**•C_6_H_6_ (9.3%) and clearly differing from that of **1**•C_6_F_6_ (19.6%), indicating exclusive incorporation of C_6_H_6_. Consistently, the crystal structure revealed an *a*‐axis length of 18.686(12) Å, supporting the formation of **1**•C_6_H_6_.

**FIGURE 6:**
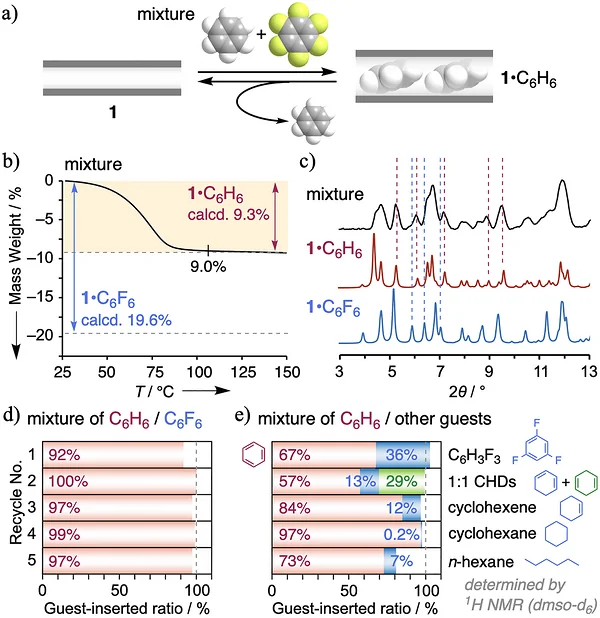
(a) Schematic illustration of the separation of a mixed C_6_H_6_/C_6_F_6_ stream by **1**. (b) TGA trace of **1** after sorption of a 1:1 C_6_H_6_/C_6_F_6_ mixture, showing **1**•C_6_H_6_. (c) Corresponding pXRD patterns (Mo*K*α; *λ* = 0.71 Å) of **1** exposed to a 1:1 C_6_H_6_/C_6_F_6_ mixture in capillary, together with SC‐XRD simulations of **1**•C_6_H_6_ and **1**•C_6_F_6_. (d) Reversible and repeated guest insertion processes (five cycles) between C_6_H_6_/C_6_F_6_ for **1**. (e) Ratio of guest selectivity between C_6_H_6_ and other guest molecules: 1,3,5‐trifluorobenzene, 1:1 mixtures of cyclohexadienes, cyclohexene, cyclohexane, and *n*‐hexane.

Quantitative uptake and C_6_H_6_ selectivity were further evaluated by immersing empty crystals of **1** in mixed guest solutions, followed by dissolution of the crystals in DMSO‐*d*_6_ to compare the proton signals by ^1^H NMR. The quantification procedure consists of three steps: (i) immersion of empty single crystals of **1** in the guest mixture, (ii) complete dissolution of the guest‐inserted crystals, and (iii) determination of the guest ratio by integrating the Py protons of **1** and the aromatic protons. Typically, when **1** was exposed to a C_6_H_6_–C_6_F_6_ (1:1) solution for 10 min, ca. 97% of C_6_H_6_ was incorporated relative to **1**•C_6_H_6_, defined as 100%. No evidence of C_6_F_6_ insertion was observed by ^13^C NMR. Repeating the wetting–drying sequence five times resulted in nearly identical uptake and selectivity, indicating that the material is reusable (Figures 6d and S24–S27).

Subsequently, competitive uptake experiments were performed using C_6_H_3_F_3_, cyclohexadienes, cyclohexane, cyclohexene, and *n*‐hexane instead of C_6_F_6_ (Figures 6e and S28–S30). In this system, selective recognition is governed by the positively polarized *Q*_zz_ region on the fluorinated π‐hole surface, providing a straightforward electrostatic basis for differentiating among nonpolar molecules. The results were unambiguous and demonstrated separation based on the selective recognition of benzene, which has a strong affinity for the π‐hole. Higher uptake of C_6_H_6_ (67%) was observed compared with molecules such as C_6_H_3_F_3_ that possess a smaller magnitude of *Q*_zz_. Remarkably, in the benzene:1,3‐cyclohexadiene:1,4‐cyclohexadiene (2:1:1) mixture, the uptake ratios were 57%, 13%, and 29%, respectively, and in the benzene:cyclohexene (1:1) mixture, the respective values were 84% and 12%. Although C_6_H_6_/cyclohexane separation has been highlighted in many recent systems [67, 68, 69], this material also exhibits 97% uptake of C_6_H_6_ with only 0.2% cyclohexane, and 73% uptake of C_6_H_6_ with 7% *n*‐hexane. These results clearly indicate that the aromatic guest with negative *Q*_zz_ is preferentially stabilized in the adaptive cavity. Notably, benzene was still preferentially incorporated even when its fraction was lower than that of cyclohexane or cyclohexene in the feed mixtures, as shown by the composition‐dependent competitive uptake experiments (Table S13, Figures S29 and S30). The decrease in total uptake only in the presence of *n*‐hexane is appropriate, given the longer molecular length of the incorporated hexane chain. Importantly, all guest uptake and release processes were fully reversible and could be repeated multiple times while maintaining SC‐to‐SC integrity. Taken together, these results show that the LAC accommodates nonpolar guests through electrostatically guided adjustments of its interlayer contacts, enabling adaptive and selective inclusion of CO_2_ and precise structural inclusion of benzene through interactions with the positively polarized fluorinated host surface.

## 3 Conclusion

The perfluorinated Zn(II) coordination sheet, [Zn(*L*)Py]_2n_ (**1**), provides a structural prototype of an interlayer guest‐adaptive crystal in which selective molecular recognition is governed not by permanent porosity but by electrostatically driven structural adaptation. The 2.6 Å ultramicropores do not operate as classical transport channels; instead, they function as electrostatic triggers that initiate guest‐induced expansion of the interlayer spacing, enabling guests larger than the intrinsic aperture to enter and assemble within the lattice. This electrostatically defined structural adaptability represents a departure from size‐based separation and conventional gate‐opening mechanisms. The key feature of this system is structural adaptability defined by electrostatic host–guest interactions at π‐hole surfaces, which enables molecular recognition and low‐pressure CO_2_ uptake in a nonporous coordination framework.

For CO_2_, quadrupole complementarity promotes uptake from very low pressure together with reversible expansion along the *a*‐axis, enabling adaptive accommodation while preserving crystallinity. The CO_2_ uptake reaches approximately two molecules per [Zn(*L*)Py]_2_ unit, and CO_2_ is selectively retained over N_2_ and CH_4_, demonstrating that electrostatic surface design, rather than brute porosity, can drive precise recognition of nonpolar gases that lack conventional binding motifs. The same principle governs aromatic inclusion. C_6_H_6_, C_6_H_3_F_3_, and C_6_F_6_ undergo reversible SC‐to‐SC transformations, yet benzene is preferentially captured. Guests adopt one‐dimensional arrays stabilized by π‐hole⋯π contacts or, in the case of C_6_F_6_, cooperative F⋯F interactions. Notably, the lattice preserves the intrinsic packing preferences of different aromatics, revealing that **1** does not merely confine guests but accommodates and amplifies their intermolecular electrostatics. These results establish interlayer electrostatic breathing as an alternative to pore‐geometry control in molecular separation. By coupling fluorinated π‐surfaces with quadrupole‐based host‐guest interactions, LACs combine the selectivity of molecular crystals with the uptake performance of porous frameworks, offering a blueprint for π‐hole‐engineered crystalline hosts capable of discriminating subtle electronic differences and stabilizing ordered guest assemblies under mild conditions. Ultimately, fluorination is what defines this adaptive behavior, transforming a rigid coordination sheet into a host capable of discriminating subtle differences in guest electrostatics among nonpolar molecules.

## Author Contributions

**Masahiro Abe**: methodology, data curation, investigation, validation, formal analysis, visualization, writing – original draft. **Tomoki Jitsukata**: conceptualization, methodology, investigation, formal analysis, writing – original draft. **Shinpei Kusaka**: methodology, investigation, formal analysis. **Ryotaro Matsuda**: conceptualization, methodology, supervision, resources. **Mikihiro Nomura**: resources, supervision. **Akiko Hori**: conceptualization, methodology, data curation, formal analysis, supervision, funding acquisition, visualization, project administration, writing – review and editing, resources.

## Conflicts of Interest

The authors declare no conflicts of interest.

## Supporting information



Synthesis and physical properties (Figure S1); crystallographic studies (Tables S1–S4 and Figures S2–S10); estimated void space (Figure S11); pXRD and TGA (Figures S12 and S13); theoretical data (Table S5 and Figure S14); adsorption data (Tables S6–S9 and Figures S15–S19); comparison with reported porous materials (Tables S10–S12 and Figure S20); breakthrough (Figures S21 and S22); aromatic recognitions for **1** (Table S13 and Figures S23–S30). Deposition Numbers 2503165 (**1**), 2503166 (**1**•C_6_H_6_), 2503167 (**1**•C_6_H_3_F_3_), and 2503168 (**1**•C_6_F_6_) contain the supplementary crystallographic data for this paper. These data are provided free of charge by the joint Cambridge Crystallographic Data Centre and Fachinformationszentrum Karlsruhe Access Structures service.
**Supporting File**: anie74303‐sup‐0001‐SuppMat.pdf.

## Data Availability

The data that support the findings of this study are available in the Supporting Information of this article.
