# Supplementary material for: Low‐Pressure CO2 and Aromatic Recognition by Interlayer Adaptive Crystal of π‐Hole‐Functionalized Zn(II) Coordination Sheets
Source: Angew Chem Int Ed Engl. 2026 Aug 24;65(42):e4090353. doi: 10.1002/anie.4090353 (PMC13653574; doi:10.1002/anie.4090353)
Supplement: Supplementary file 1 [file ANIE-65-e4090353-s001.pdf]

Supporting Information

Low-Pressure CO_2 and Aromatic Recognition by Interlayer Adaptive Crystal of π-Hole-Functionalized Zn(II) Coordination Sheets

M. Abe, T. Jitsukata, S. Kusaka, R. Matsuda, M. Nomura and A. Hori*

*Corresponding author (ahori@shibaura-it.ac.jp)

Table of Contents

S1. Preparation and physical properties of **1** ··· 2

S2. Crystal data of **1**, **1**•C_6H_6, **1**•$C_6H_3F_3$, and **1**•C_6F_6 ··· 3

S3. Main intermolecular interactions in the crystals ··· 9

S4. Structural information for the hydrogen analogue of **1** ··· 9

S5. Estimated void space of **1** ··· 11

S6. pXRD and thermogravimetric (TG) studies ··· 12

S7. Dipole and quadrupole moments and related parameters for guest molecules ··· 13

S8. Gas adsorption isotherms ··· 14

S9. CO_2 adsorption and the corresponding isosteric heat of adsorption (Q_{st}) of **1** ··· 19

S10. pXRD under a CO_2 atmosphere ··· 20

S11. Comparison with reported porous framework materials ··· 22

S12. Breakthrough measurements of CO_2/N_2 and CO_2/CH_4 mixtures ··· 23

S13. pXRD of guest-soaked crystals ··· 24

S14. NMR spectra of guest-inserted **1** in a C_6H_6-C_6F_6 (1:1) mixture ··· 25

S15. Competitive guest uptake determined by ^{1}H NMR ··· 28

S1. Preparation and physical properties of 1

(1) General

Reagents and solvents were used for synthesis and host-guest investigations without further purification. ^{1}H and ^{13}C NMR spectra were recorded on a JEOL JNM-ECZL400S spectrometer with tetramethylsilane as an internal standard. Thermogravimetric (TG) analysis was performed using a TA Instruments TGA-Q500. Gas adsorption isotherms were obtained using a MicrotracBEL BELSORP MAX II. Low-temperature gas adsorption measurements were performed using a Bell cryostat for temperature control. Breakthrough measurements were performed using a BELCAT II system connected to a BELMass quadrupole mass spectrometer. The ESP was calculated by DFT using the Spartan'20 (Wavefunction Inc.) package (V1.1.4) at the ωB97X-D/6-31G* level.

(2) Synthesis of **1** (**Figure S1**)

A methanol solution (10 mL) containing tetrafluoroisophthalic acid (119 mg, 0.50 mmol) and zinc acetate dihydrate (110 mg, 0.50 mmol) was prepared. A hexane solution of pyridine (0.80 mmol, 10 mL) was then carefully layered on top of the methanol solution, and colorless prismatic crystals of **1** were obtained by liquid-liquid diffusion. The product was isolated to give **1** (0.184 g, 97% yield). Elemental analysis data were consistent with the formula of **1**: calcd. for $C_{26}H_{10}F_8N_2O_8Zn_2\cdot 3H_2O$ (%): C 38.31, H 1.98, N 3.44; found: C 38.61, H 1.79, N 3.68.

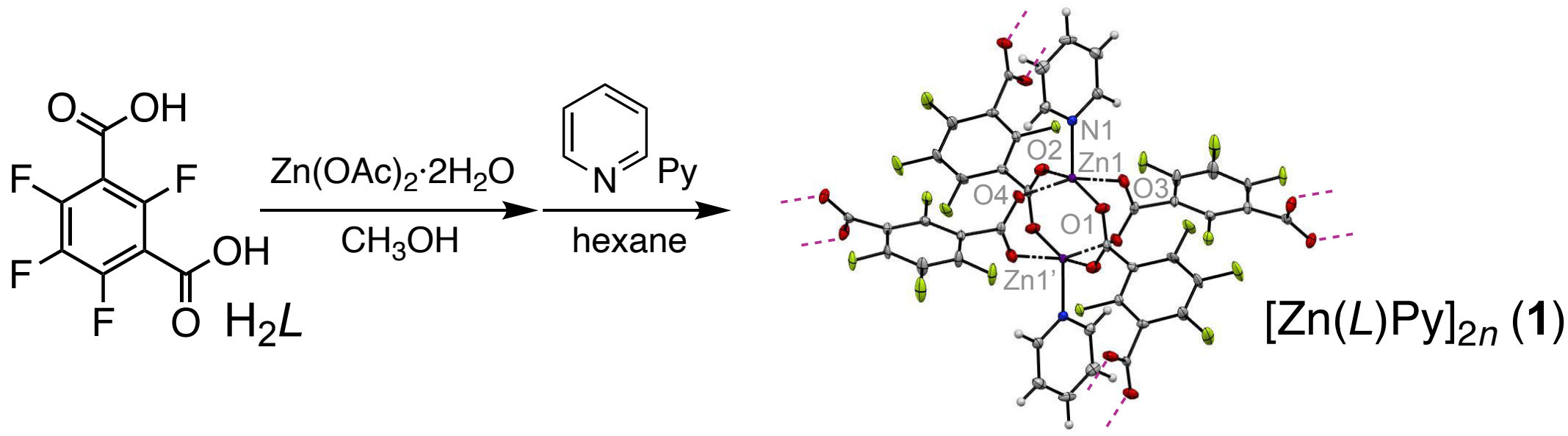

Figure S1. Synthesis of **1**.

S2. Crystal data of 1, 1•C_6H_6, 1•$C_6H_3F_3$, and 1•C_6F_6

(1) Crystal structure determination

The single-crystal X-ray structures were determined using a Bruker D8 Quest with Mo*K*α radiation (λ = 0.71073 Å) generated at 50 kV and 1 mA. Crystals were coated with Paratone-N oil prior to data collection. Powder X-ray diffraction (pXRD) patterns were collected using (i) a Bruker D8 Quest diffractometer with Mo*K*α radiation (λ = 0.71073 Å, 50 kV, 1 mA) and (ii) a Rigaku SmartLab diffractometer with Cu*K*α radiation (λ = 1.541862 Å, 40 kV, 50 mA).

(2) Crystal data of **1**

The crystal data of **1** are shown in **Table S1**.

Table S1. Crystal data and structure refinements for **1**.

	**1**
Chemical formula	$C_{13}H_5F_4NO_4Zn$
Description, color	Prismatic, colorless
Crystal size [mm]	0.55 × 0.13 × 0.08
Formula weight	380.55
T [K]	100
Crystal system	Monoclinic
Space group	$C2/c$
a [Å]	16.1114 (8)
b [Å]	12.3499 (6)
c [Å]	14.2728 (7)
β [°]	95.859 (2)
V [Å^3]	2825.1 (2)
Z	8
D_c [g cm^{-3}]	1.789
F(000)	1504
R_{int}	0.097
Reflections measured	15499
Reflections independent	2490
GOF	1.055
R [(I) > 2σ(I)]	0.0329
wR (F_o^2)	0.0791
CCDC No.	2503165

(3) Molecular and packing structures of **1**

For crystal **1**, the molecular and packing structures are shown in **Figures S2** and **S3**, respectively. The asymmetric unit contains half of the complex, [Zn(L)Py]. H atoms attached to C atoms were refined as riding on their idealized positions, with C-H = 0.95 Å for aromatic C–H bonds.

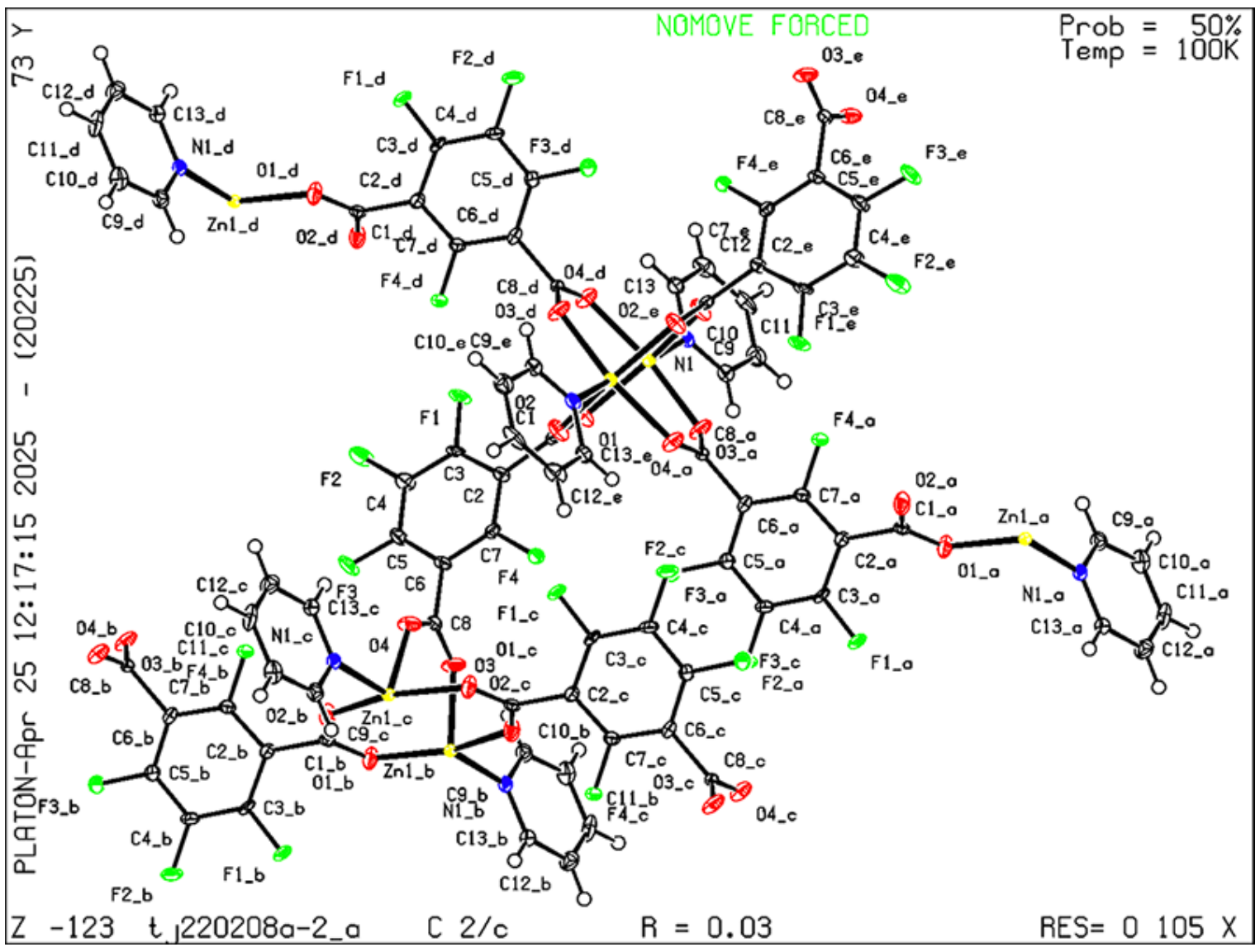

Figure S2. The molecular structure of **1** obtained using PLATON (checkCIF).

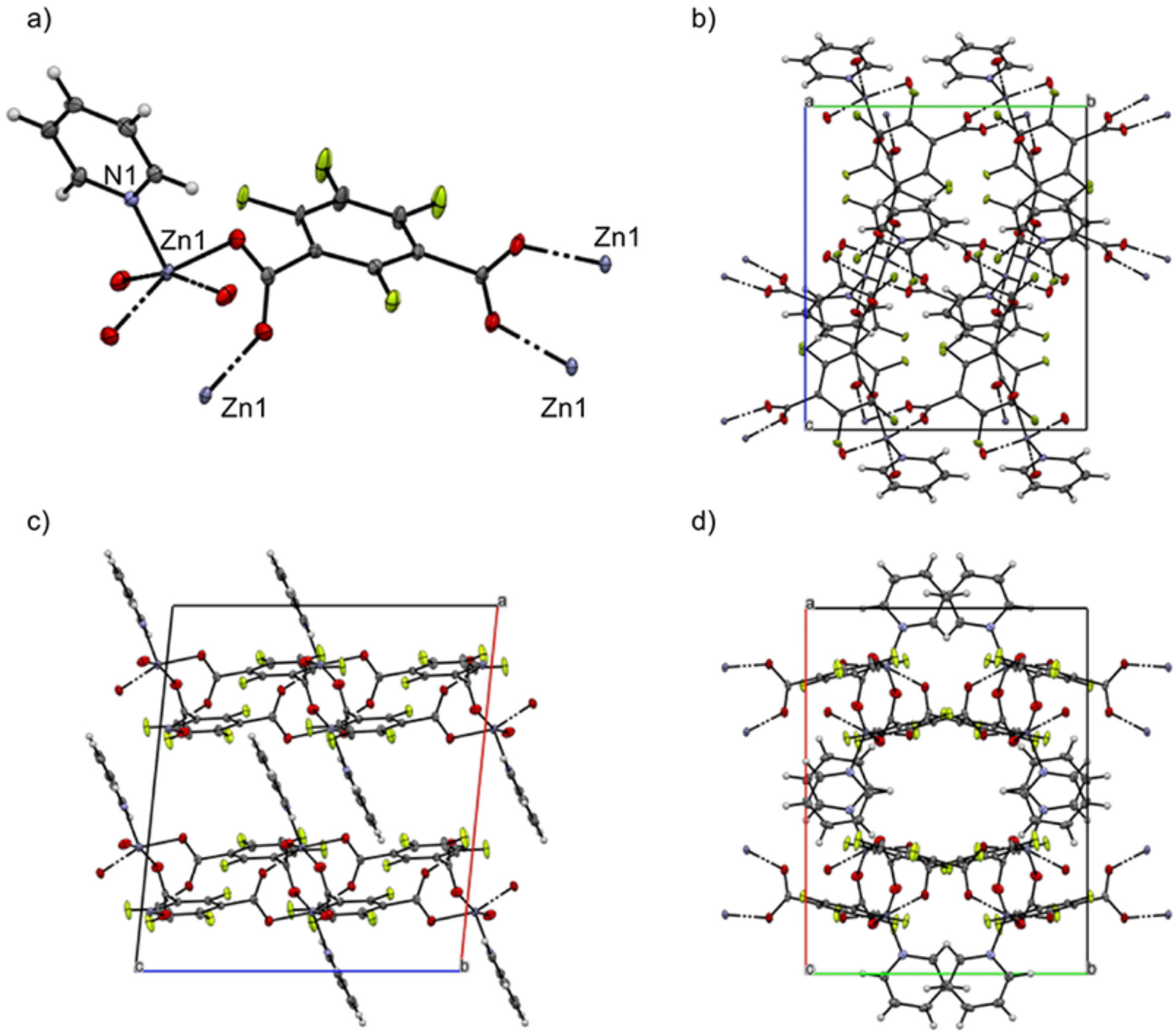

Figure S3. a) Molecular structure of **1**, and crystal packing structures viewed along the b) *a*-axis, c) *b*-axis, and d) *c*-axis.

(4) Crystal data of **1**•C_6H_6, **1**•$C_6H_3F_3$ and **1**•C_6F_6

The crystal data of the aromatic-loaded crystals are shown in **Table S2**.

Table S2. Crystal data and structure refinements for **1**•C_6H_6, **1**•$C_6H_3F_3$, and **1**•C_6F_6.

	**1**•C_6H_6	**1**•$C_6H_3F_3$	**1**•C_6F_6
Chemical formula	$C_{13}H_5F_4NO_4Zn$•0.5(C_6H_6)	2($C_{13}H_5F_4NO_4Zn$)•$C_6H_3F_3$	$C_{13}H_5F_4NO_4Zn$•0.5(C_6F_6)
Description, color	Prismatic, colorless	Prismatic, colorless	Prismatic, colorless
Crystal size [mm]	0.32 × 0.20 × 0.09	0.28 × 0.18 × 0.14	0.26 × 0.18 × 0.08
Formula weight	419.60	893.18	473.58
T [K]	200	200	200
Crystal system	Monoclinic	Monoclinic	Monoclinic
Space group	$C2/c$	$C2/c$	$C2/c$
a [Å]	18.943 (7)	19.14 (1)	17.845 (2)
b [Å]	12.457 (5)	12.566 (6)	12.7339 (15)
c [Å]	13.595 (5)	13.780 (7)	14.0076 (16)
β [°]	102.822 (10)	104.269 (14)	100.076 (3)
V [Å^3]	3128 (2)	3212 (3)	3133.9
Z	8	4	8
D_c [g cm^{-3}]	1.782	1.847	2.007
F(000)	1672	1768	1864
R_{int}	0.100	0.084	0.085
Refl. measured	12663	11480	13288
Refl. independent	2749	2837	2762
GOF	1.014	1.050	1.048
R [(I) > 2σ(I)]	0.0469	0.0592	0.0548
wR (F_o^2)	0.0839	0.1578	0.1519
CCDC No.	2503166	2503167	2503168

(5) Molecular and packing structures of **1**•C_6H_6

For crystal **1**•C_6H_6, the molecular and packing structures are shown in **Figures S4** and **S5**, respectively. The asymmetric unit contains half of the complex, [Zn(L)Py], together with half of a benzene molecule. H atoms attached to C atoms were refined as riding on their idealized positions, with C-H = 0.95 Å for aromatic C–H bonds.

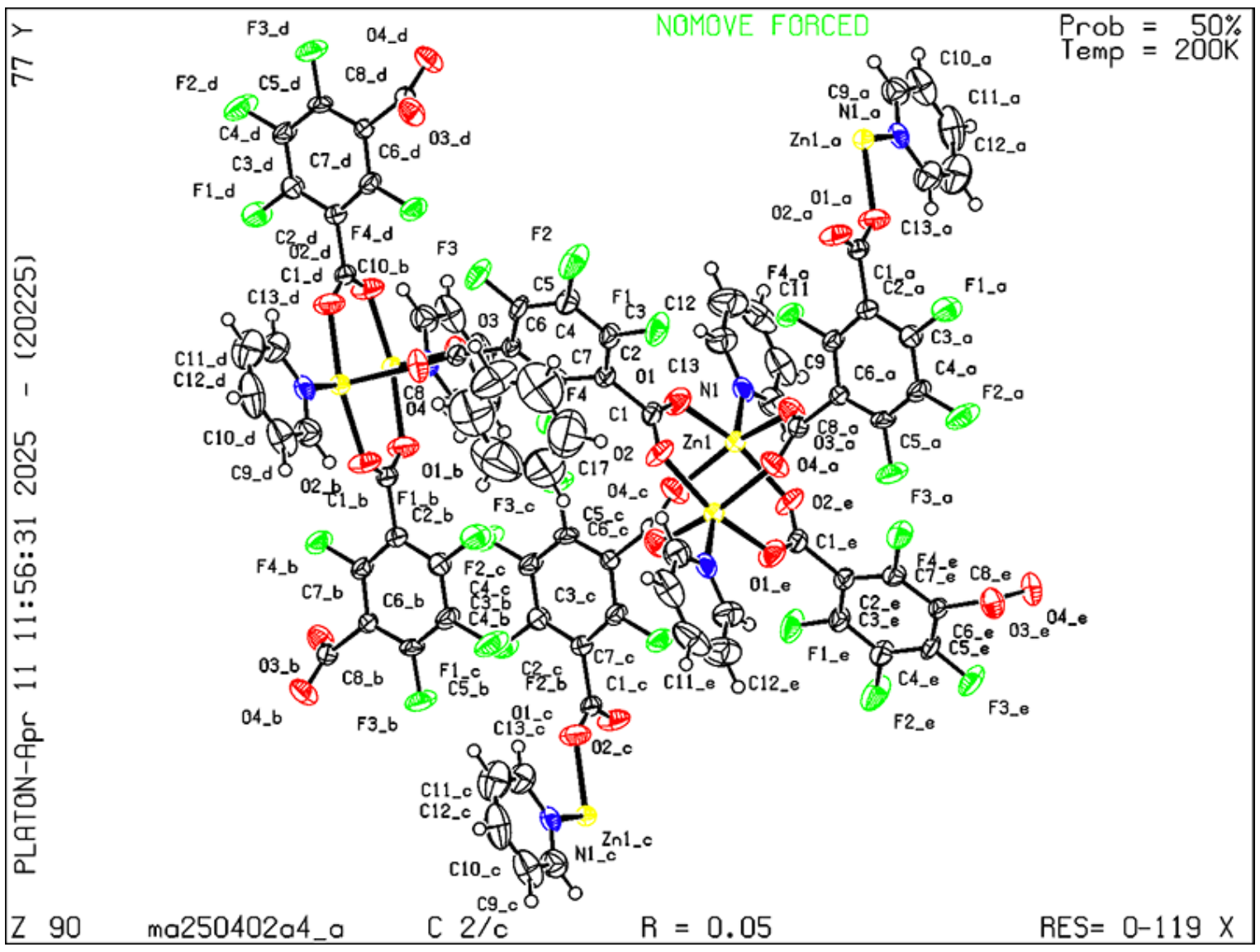

Figure S4. The molecular structure of **1**•C_6H_6 obtained using PLATON (checkCIF).

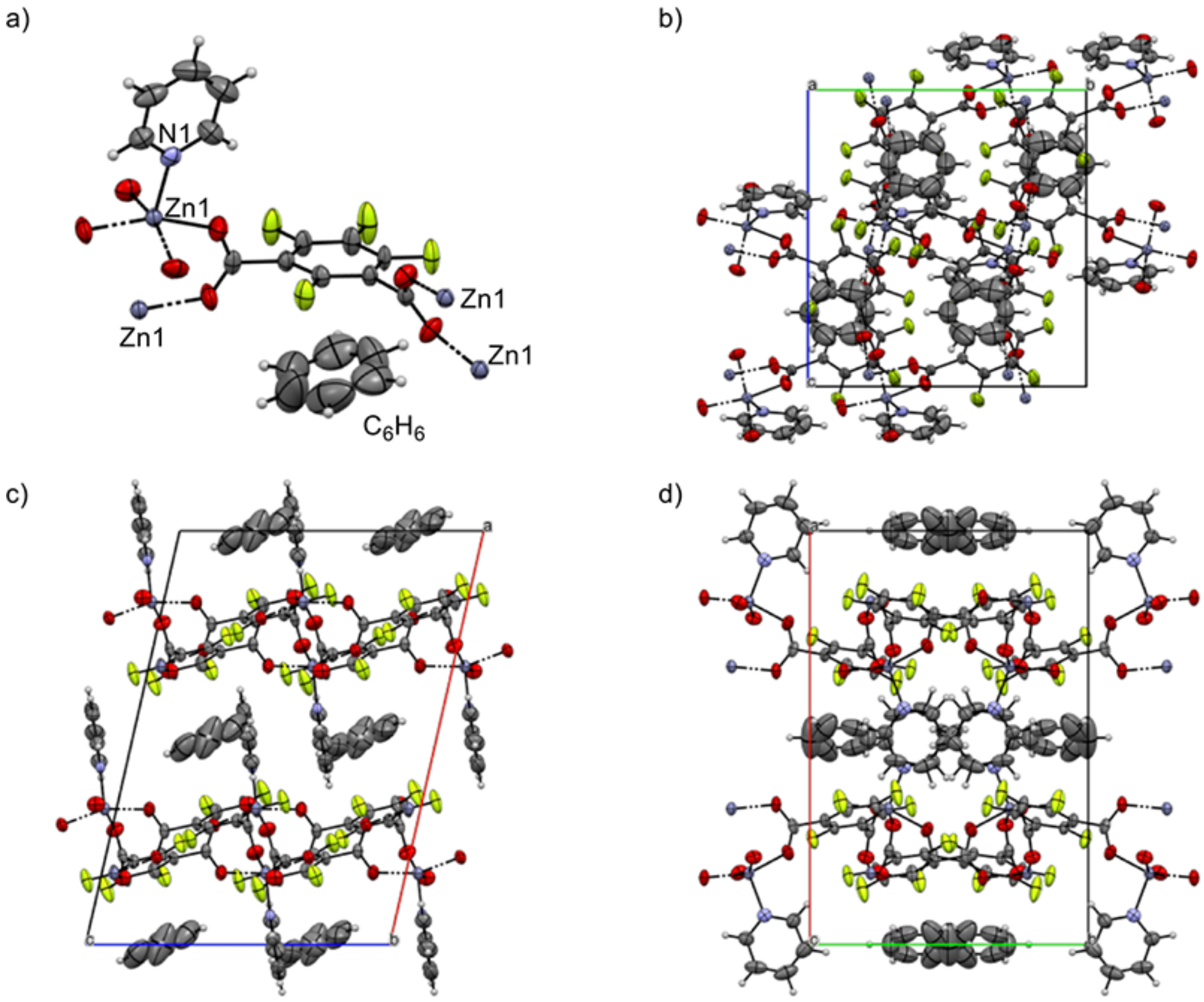

Figure S5. a) Molecular structure of **1**•C_6H_6, and crystal packing structures viewed along the b) *a*-axis, c) *b*-axis, and d) *c*-axis.

(6) Molecular and packing structures of **1**•$C_6H_3F_3$

For crystal **1**•$C_6H_3F_3$, the molecular and packing structures are shown in **Figures S6** and **S7**, respectively. The asymmetric unit contains half of the complex, [Zn(L)Py], together with half of a trifluorobenzene molecule. H atoms attached to C atoms were refined as riding on their idealized positions, with C-H = 0.95 Å for aromatic C–H bonds.

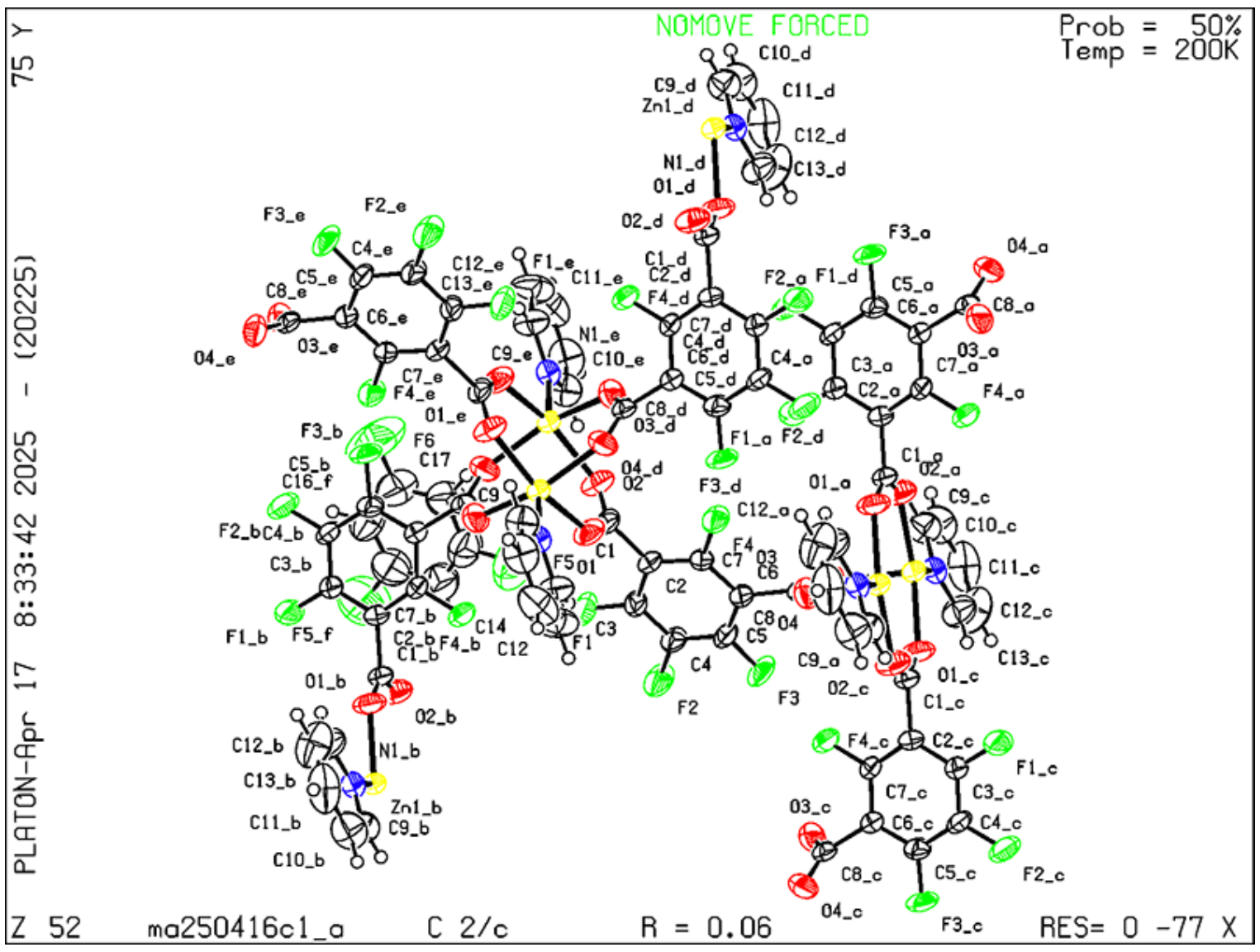

Figure S6. The molecular structure of **1**•$C_6H_3F_3$ obtained using PLATON (checkCIF).

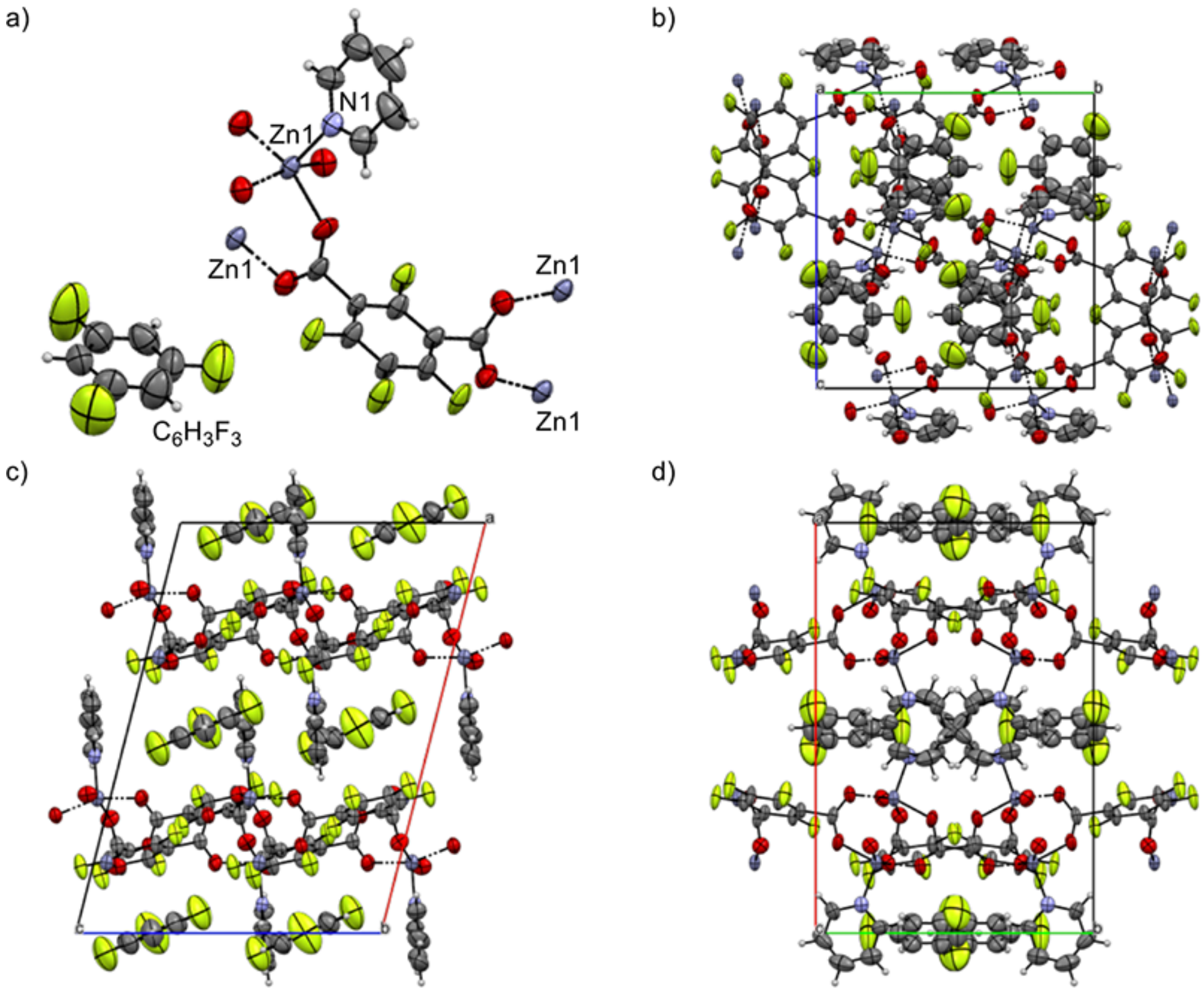

Figure S7. a) Molecular structure of **1**•$C_6H_3F_3$ and crystal packing structures viewed along the b) *a*-axis, c) *b*-axis, and d) *c*-axis.

(7) Molecular and packing structures of **1**•C_6F_6

For crystal **1**•C_6F_6, the molecular and packing structures are shown in **Figures S8** and **S9**, respectively. The asymmetric unit contains half of the complex, [Zn(L)Py], together with half of a hexafluorobenzene molecule. H atoms attached to C atoms were refined as riding on their idealized positions, with C-H = 0.95 Å for aromatic C–H bonds.

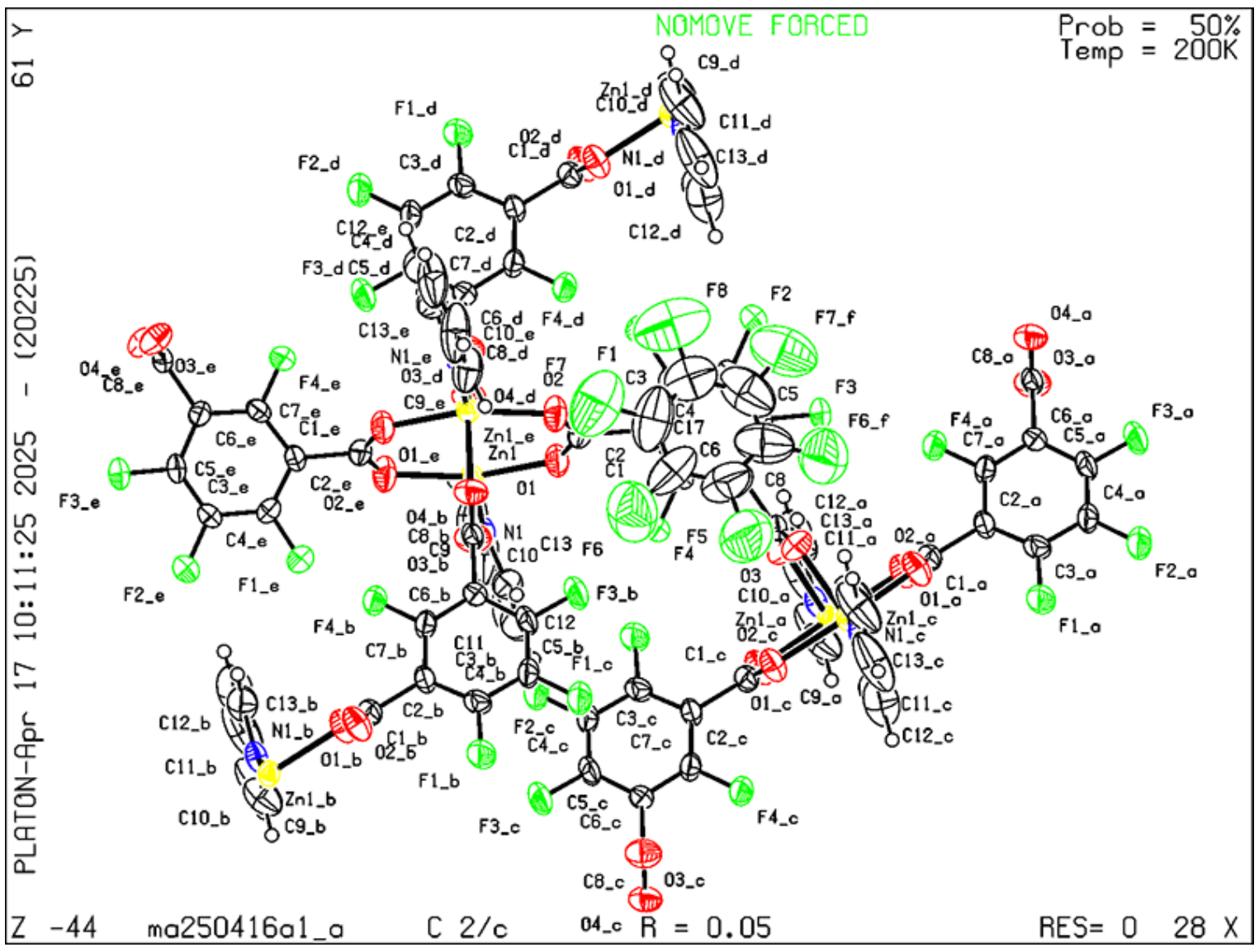

Figure S8. The molecular structure of **1**•C_6F_6 obtained using PLATON (checkCIF).

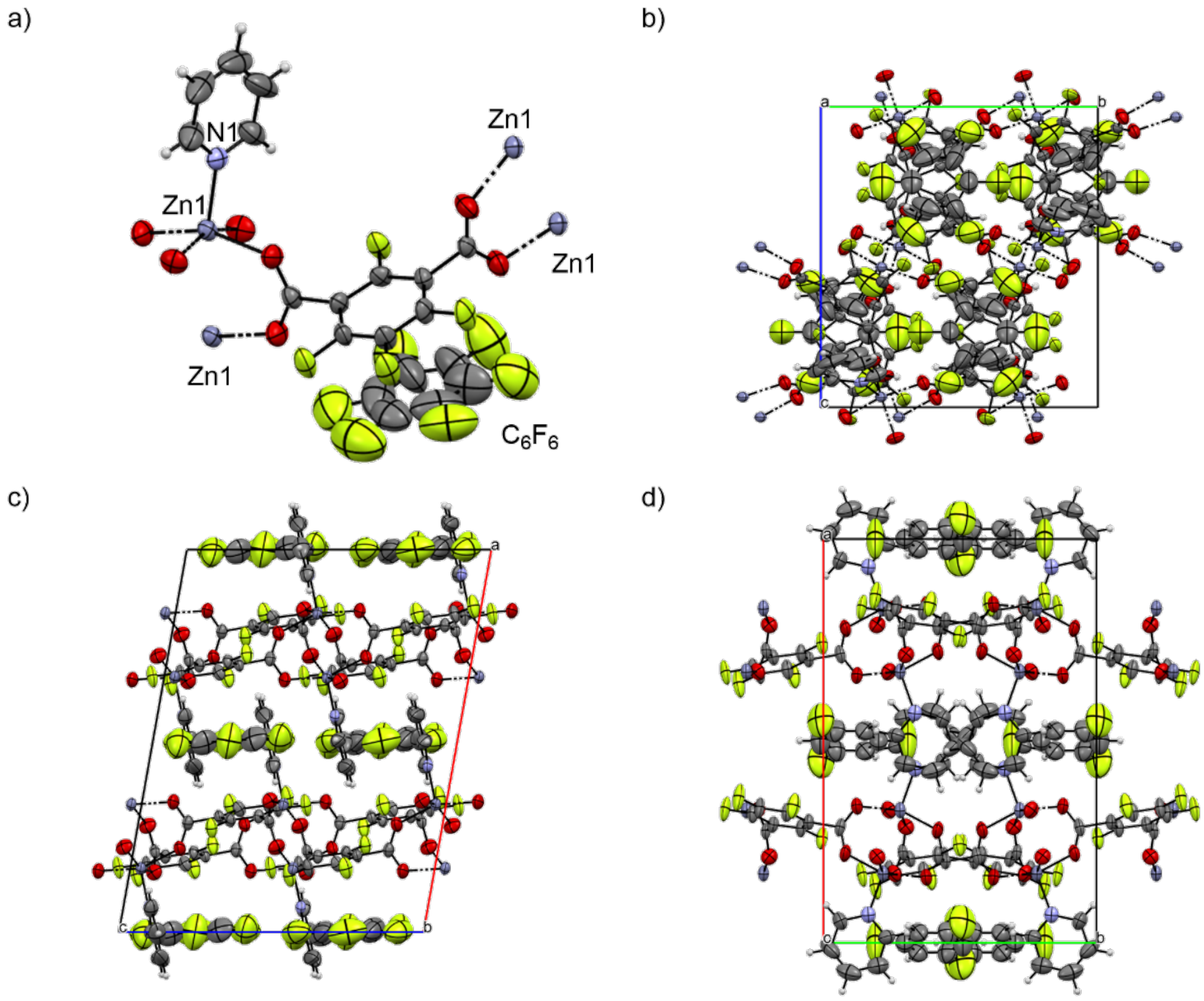

Figure S9. a) Molecular structure of **1**•C_6F_6, and crystal packing structures viewed along the b) *a*-axis, c) *b*-axis, and d) *c*-axis.

S3. Main intermolecular interactions in the crystals

The significant intermolecular contacts were extracted from the PLATON "short-contact" analysis. Distances shorter than the sum of the van der Waals radii were identified and classified into π-hole···π, C–H···O, C–H···F, and F···F interactions.

Table S3. Remarkable intermolecular interactions for **1**, **1**•C_6H_6, **1**•$C_6H_3F_3$, and **1**•C_6F_6.

Crystals	π-hole···π	C–H···O	C–H···F	F···F
**1**	no	no	Exist (3.34, 3.40 Å)	Exist (2.92, 3.13 Å)
**1**•C_6H_6	Exist (3.66 Å)	no	no	Exist (2.74 Å)
**1**•$C_6H_3F_3$	Exist (3.68 Å)	no	Exist (3.24 Å)	Exist (2.71 Å)
**1**•C_6F_6	Exist (3.57 Å)	no	Exist (2.84, 3.20, 3.37 Å)	Exist (2.77, 3.09 Å)

S4. Structural information for the hydrogen analogue of 1

The hydrogen analogue of **1** (CCDC152547), reported previously by M. J. Zaworotko [ref. S1], possesses the same dinuclear metal framework but crystallizes in a different space group and is therefore not isomorphous with the structure described here.

Table S4. Crystal data for **1** and non-fluorinated analogue **1'**.

	**1**	**1'** (non-fluorinated complex)
Chemical formula	$C_{13}H_5F_4NO_4Zn$	$C_{38}H_{30}N_2O_8Zn_2$
Crystal system	Monoclinic	Tetragonal
Space group	*C*2/*c*	*P*4/*ncc*
a [Å]	16.1114 (8)	19.0356 (9)
b [Å]	12.3499 (6)	19.0356 (9)
c [Å]	14.2728 (7)	16.2530 (11)
β [°]	95.859 (2)	90.00
V [Å^3]	2825.1 (2)	5889.3 (6)
Z	8	8

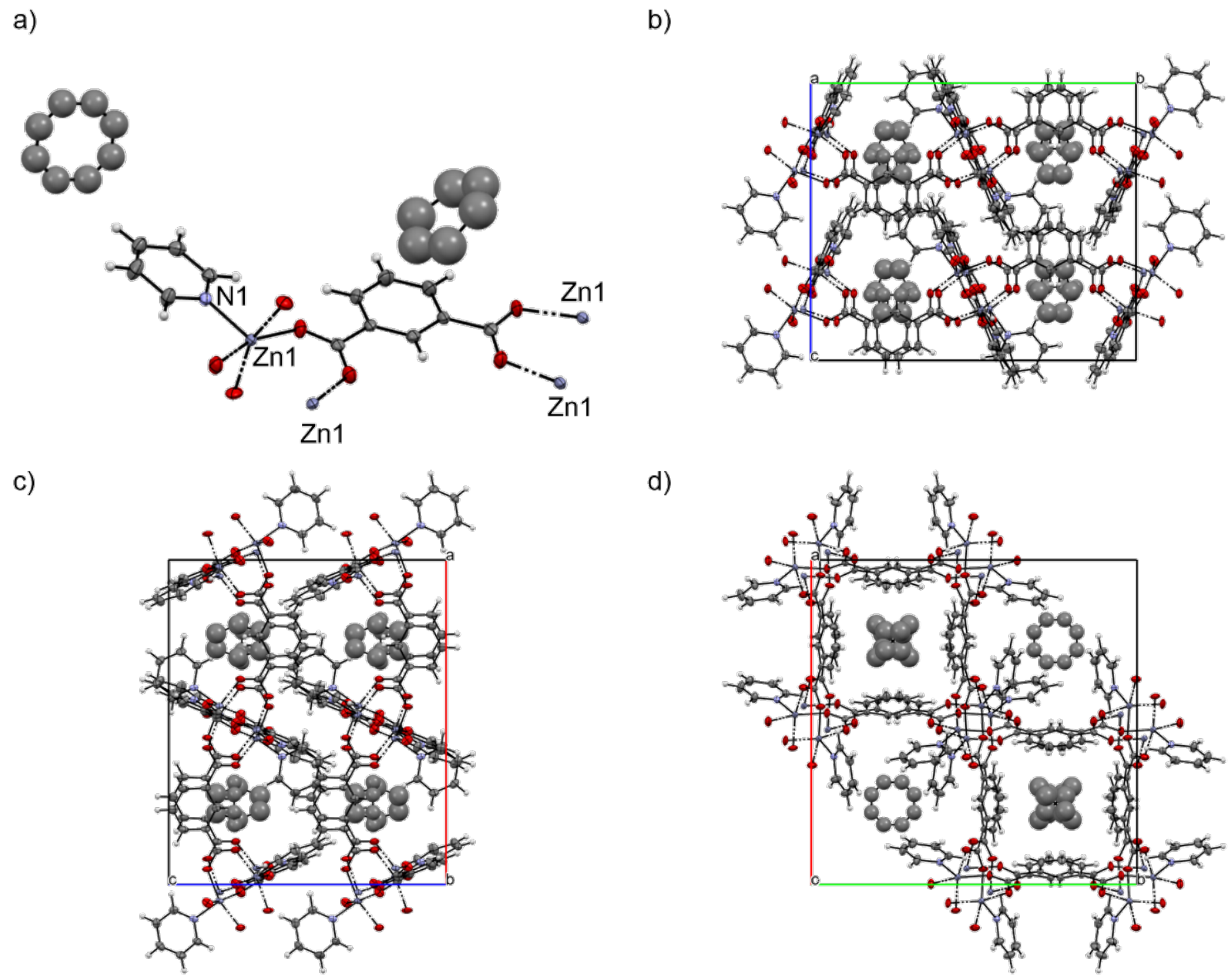

Figure S10. a) Molecular structure of **1'**, and crystal packing structures viewed along the b) *a*-axis, c) *b*-axis, and d) *c*-axis.

S5. Estimated void space of 1

The unoccupied spaces (voids) within the crystal unit cells were determined by evaluating whether a spherical probe of a specified radius could be accommodated. A probe radius of 1.2 Å, corresponding to the van der Waals radius of hydrogen, was used. Additional analyses were performed using probe radii of 1.2-1.7 Å (H: 1.2 Å, O: 1.52 Å, N: 1.55 Å, C: 1.70 Å) and grid spacing of 0.3 Å (**Figure S11**). Void calculations were performed using Mercury (probe routine).

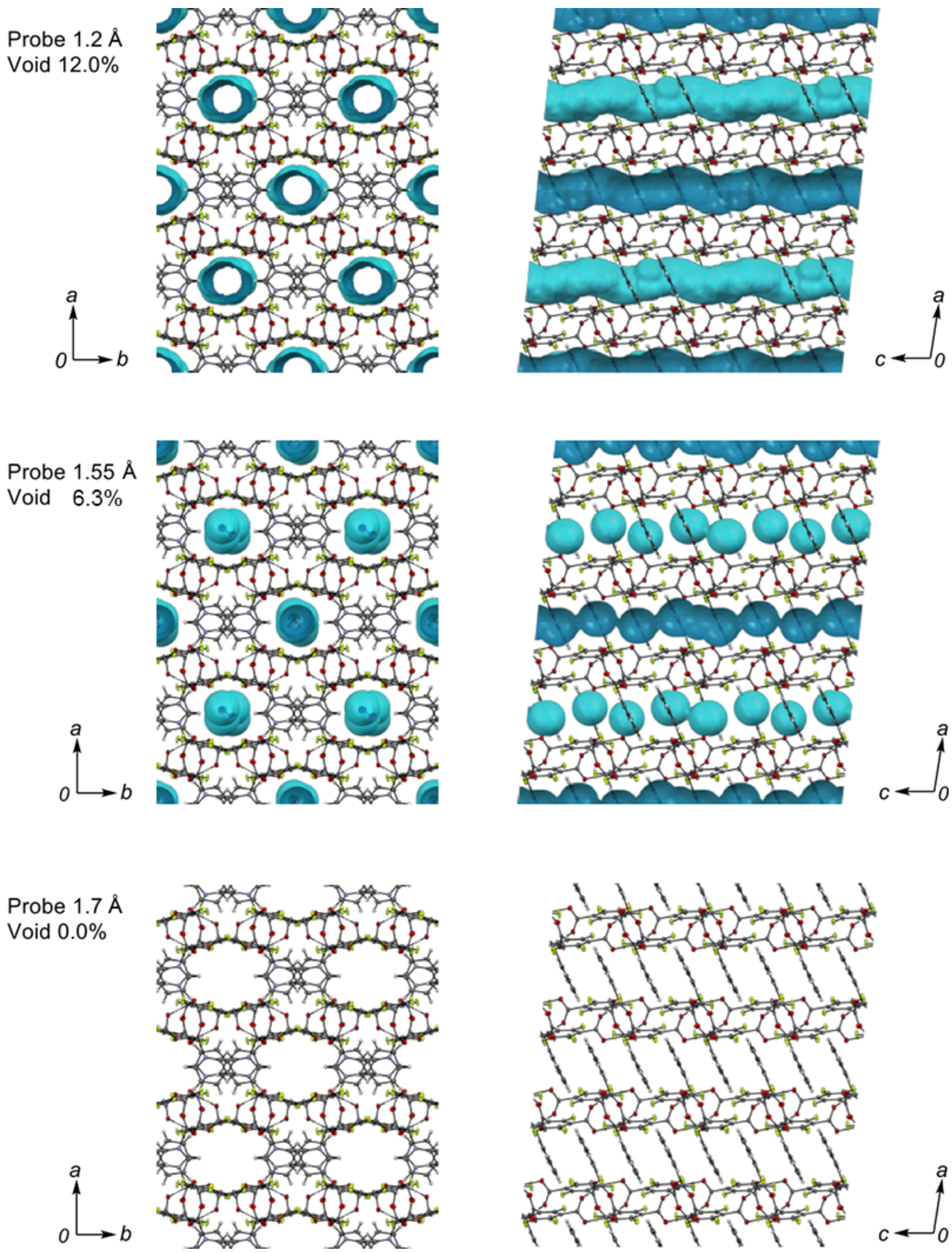

Figure S11. Spherical probe analysis and corresponding void volume of **1**.

S6. pXRD and thermogravimetric (TG) studies

(1) Powder X-ray diffraction (pXRD) measurements

Data were obtained using a Bruker D8 Quest diffractometer equipped with Mo*K*α radiation (λ = 0.71073 Å). All measurements were conducted using the capillary method.

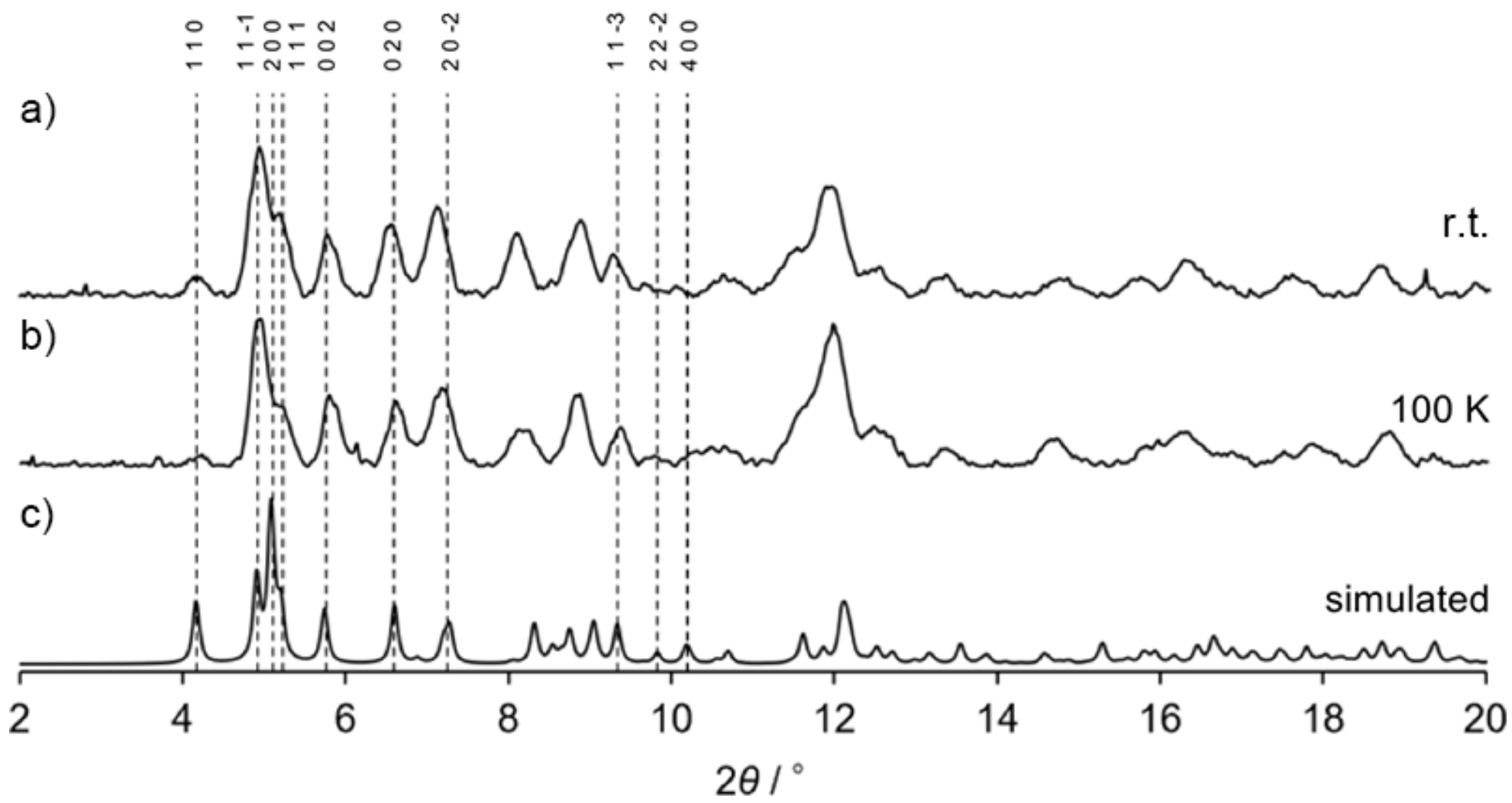

Figure S12. pXRD patterns obtained using the capillary method: a) room temperature, b) 100 K, and c) simulated pattern from SC-XRD of **1**.

(2) TG analysis

TG analysis shows no significant weight change below 230 °C (**Figure S13**). A minor mass loss of −5.0% at 240.1 °C precedes rapid decomposition above 240 °C, indicating thermal stability up to this temperature. TG measurements were carried out on an 8.17 mg sample under a flow of He at a heating rate of 5 °C min^{-1}.

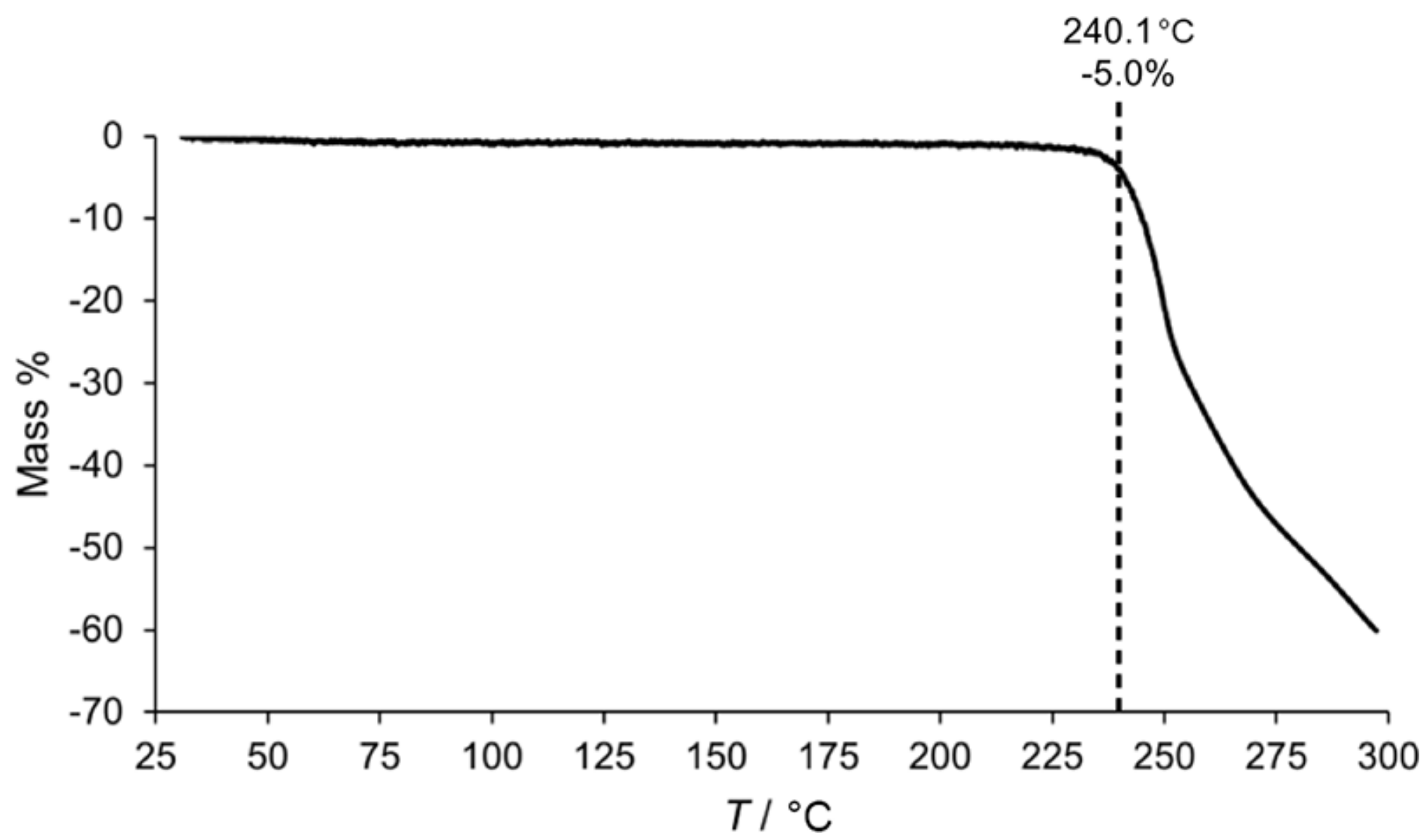

Figure S13. TG curve (heating rate: 5 °C min^{-1}).

S7. Dipole and quadrupole moments and related parameters for guest molecules

The ESPs of guest molecules were calculated by DFT at the ωB97X-D/6-31G* level (**Figure S14**).

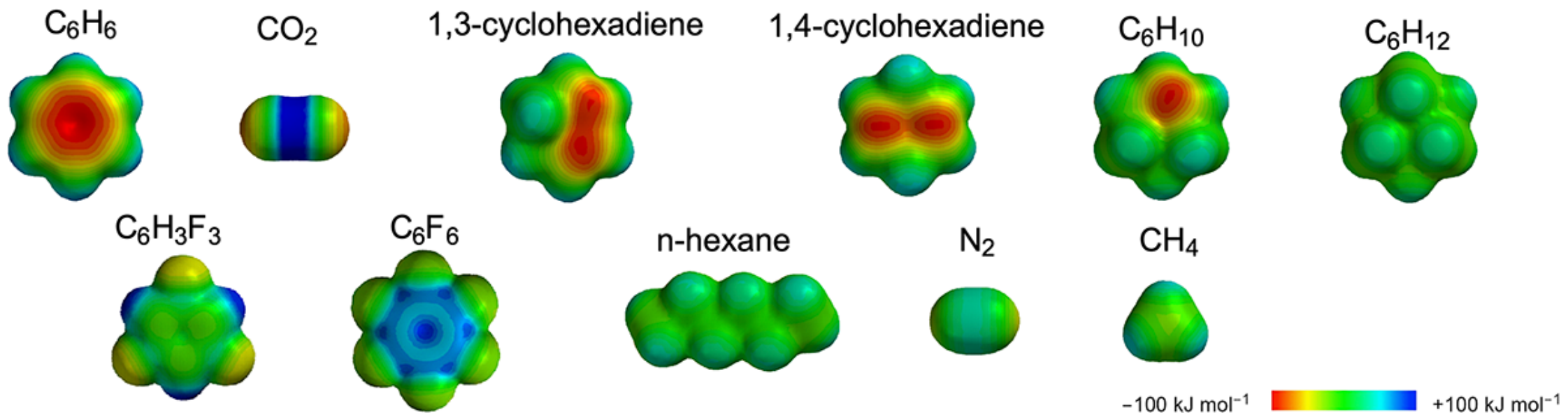

Figure S14. ESP maps of the guest molecules; color scheme: −100 kJ mol^{-1} (red) and +100 kJ mol^{-1} (blue).

Table S5. Experimental quadrupole moments (10^{-40} C·m^2), ESP values at the aromatic centroid along the Q_{zz} direction (kJ mol^{-1}), and melting and boiling points for selected guest molecules [ref. S2].

Compounds	Formula	Q_{xx} / C m^2	Q_{yy} / C m^2	Q_{zz} / C m^2	Q *[1] / C m^2	ESP of C_g *[2] / kJ mol^{-1}	m.p. /°C	b.p. /°C
benzene	C_6H_6	+14.5	+14.5	–29.0	–29.0	–94	5.5	80
carbon dioxide	CO_2	+7.2	+7.2	–14.4	–14.4	–67	−57*[3]	−78
1,3-cyclohexadiene	C_6H_8	−15.3	+6.03	+9.22	+9.22	−73	−89	81
1,4-cyclohexadiene	C_6H_8	+12.8	+3.05	−15.9	−15.9	−72	−49	82
cyclohexene	C_6H_{10}	−6.01	+5.31	+7.01	+7.01	−44	−104	83
cyclohexane	C_6H_{12}	−9.0	−0.90	+1.80	+1.80	−5.3	6.5	81
1,3,5-trifluorobenzene	$C_6H_3F_3$	−1.2	+2.3	−1.1	−1.1	+6.0	−5.5	75
hexafluorobenzene	C_6F_6	−16.8	−16.8	+33.6	+33.6	+98	5.0	80
n-hexane	C_6H_{14}	n/a	n/a	n/a	n/a	−66	−94	69
nitrogen	N_2	+2.3	+2.3	–4.7	–4.7	−48	−210	−196
methane	CH_4	n/a	n/a	n/a	n/a	−22	−182	−161

*[1] Because the principal axis (z-axis) varies depending on the position of the substituents, the main quadrupole moment (Q) in six-membered ring structures represents the in-plane value, whereas for other structures it corresponds to Q_{zz}, the component perpendicular to the *z*-axis. *[2] ESP values were obtained by DFT (ωB97X-D/6-31G*). *[3] CO_2 undergoes sublimation, triple point is indicated.

S8. Gas adsorption isotherms

Adsorption and desorption isotherms of N_2, CO_2, and CH_4 measured at different temperatures are summarized below (**Figure S15**). The adsorbed amounts are expressed per [Zn(*L*)Py]$_2$ unit. The selected data were summarized in **Table S6-S9** (Values used in the manuscript are highlighted in yellow).

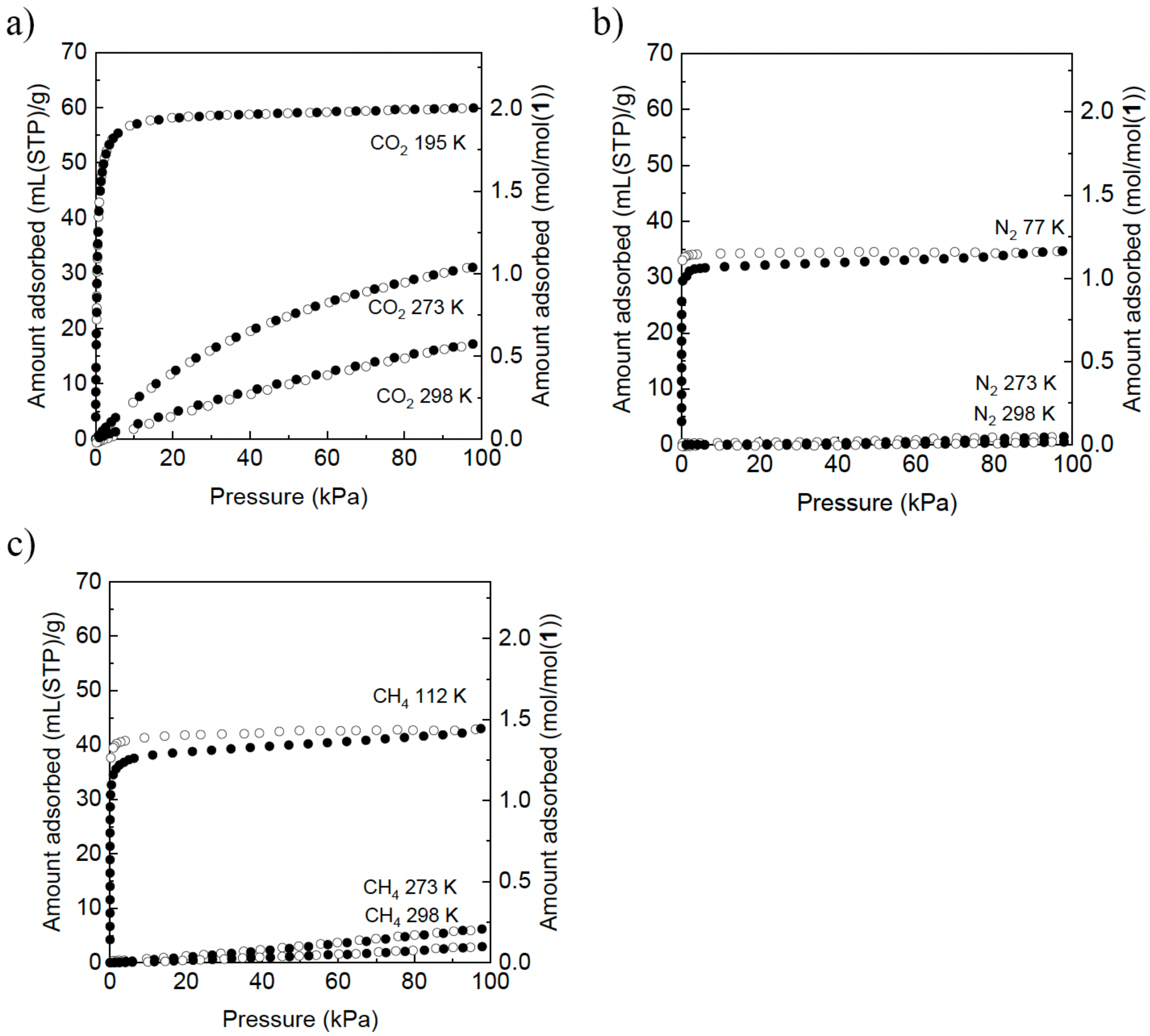

Figure S15. Temperature dependence of a) CO_2, b) N_2, and c) CH_4 isotherms.

Table S6. CO_2 adsorption/desorption data at 195 K.

	Adsorption			Desorption	
P / kPa	V / mL(STP)	n_a / mol mol^{-1}	P / kPa	V / mL(STP)	n_a / mol mol^{-1}
0.02002	3.9796	0.135	94.887	59.876	2.03
0.03598	6.2624	0.213	90.451	59.721	2.03
0.05552	8.5272	0.290	86.027	59.647	2.03

0.07881	10.744	0.365	80.141	59.649	2.03
0.10655	12.947	0.440	75.756	59.525	2.02
0.17033	17.063	0.580	69.966	59.395	2.02
0.20414	19.08	0.648	65.626	59.249	2.01
0.27104	22.932	0.779	59.84	59.189	2.01
0.31686	25.592	0.869	55.527	59.082	2.01
0.36489	28.167	0.957	49.769	58.978	2.00
0.41753	30.676	1.04	44.076	58.84	2.00
0.48088	33.054	1.12	39.771	58.771	2.00
0.56076	35.338	1.20	34.036	58.675	1.99
0.65117	37.461	1.27	29.723	58.565	1.99
0.862	41.238	1.40	24.024	58.382	1.98
1.1884	44.891	1.53	19.787	58.167	1.98
1.407	46.637	1.58	14.2	57.689	1.96
1.7043	48.295	1.64	8.8774	56.735	1.93
2.0626	49.802	1.69	4.3182	54.317	1.85
2.6886	51.623	1.75	3.4933	53.327	1.81
3.566	53.248	1.81	2.8072	52.134	1.77
4.5977	54.466	1.85	2.286	50.845	1.73
5.8118	55.385	1.88	1.9391	49.626	1.69
10.737	57.07	1.94	1.5012	47.538	1.62
16.387	57.792	1.96	1.1646	44.958	1.53
21.68	58.17	1.98	0.96491	42.861	1.46
26.828	58.386	1.98	0.77711	40.2	1.37
31.914	58.599	1.99	0.6432	37.589	1.28
37.024	58.726	2.00	0.53807	35.082	1.19
42.095	58.841	2.00	0.45762	32.653	1.11
47.169	58.958	2.00	0.39881	30.256	1.03
52.233	59.08	2.01	0.35455	28.08	0.954
57.324	59.13	2.01	0.31425	25.878	0.879
62.369	59.318	2.02	0.27768	23.726	0.806
67.438	59.39	2.02	0.24269	21.641	0.735
72.557	59.415	2.02			
77.551	59.649	2.03			
82.651	59.671	2.03			
87.706	59.783	2.03			
92.721	59.936	2.04			
97.873	59.932	2.04			

Table S7. CO_2 adsorption data (very low pressure) at 195 K.

P / kPa	n_a / mol mol^{-1}	P / kPa	n_a / mol mol^{-1}	P / kPa	n_a / mol mol^{-1}
0	0	0.14406	0.600	0.66682	1.36
2.0295E-03	0.0196	0.15393	0.623	0.70006	1.38
3.4915E-03	0.0342	0.1633	0.646	0.73177	1.40
4.9546E-03	0.0486	0.17259	0.668	0.76005	1.41
6.4789E-03	0.0630	0.18165	0.689	0.7913	1.43
8.0355E-03	0.0771	0.19053	0.710	0.83921	1.45
9.6262E-03	0.0909	0.19912	0.730	0.88188	1.47
0.01131	0.105	0.20736	0.750	0.91628	1.48
0.012967	0.119	0.21566	0.770	0.95395	1.50
0.014586	0.132	0.22366	0.789	0.98874	1.51
0.017579	0.151	0.23126	0.807	1.0007	1.51
0.020608	0.170	0.23918	0.826	2.0554	1.72
0.023362	0.188	0.24643	0.844	3.6554	1.82
0.026115	0.206	0.25402	0.861	4.6173	1.85
0.029375	0.223	0.26133	0.878	5.0433	1.86
0.032639	0.240	0.26848	0.894	9.4365	1.91
0.035439	0.256	0.27575	0.911	10	1.91
0.038888	0.273	0.28293	0.926	14.86	1.93
0.04259	0.288	0.28999	0.942	15.04	1.93
0.046104	0.303	0.30293	0.968	20.05	1.95
0.049271	0.317	0.31506	0.992	25	1.95
0.052949	0.332	0.32773	1.02	30.05	1.96
0.056407	0.346	0.34007	1.04	35.01	1.96
0.059658	0.359	0.3534	1.06	40.01	1.97
0.063531	0.372	0.3659	1.08	45.02	1.97
0.066751	0.385	0.37874	1.10	50.03	1.97
0.071555	0.400	0.39153	1.11	54.99	1.98
0.076325	0.416	0.40448	1.13	60.03	1.98
0.081314	0.430	0.41657	1.15	65.04	1.98
0.085664	0.444	0.42854	1.16	70.07	1.98
0.089918	0.457	0.44468	1.18	75.09	1.98
0.094242	0.469	0.45981	1.19	80.08	1.98
0.098522	0.481	0.47315	1.20		
0.1024	0.492	0.48624	1.22		
0.10703	0.505	0.49866	1.23		
0.11156	0.517	0.50135	1.23		

0.1158	0.528	0.53792	1.26
0.11995	0.540	0.57046	1.29
0.12474	0.551	0.60272	1.32
0.13422	0.576	0.63512	1.34

Table S8. N_2 adsorption/desorption data at 77 K.

Adsorption			Desorption		
P / kPa	*V* / mL(STP)	n_a / mol mol^{-1}	*P* / kPa	*V* / mL(STP)	n_a / mol mol^{-1}
0.0034	4.1677	0.142	96.206	34.608	1.18
0.00474	6.5835	0.224	90.937	34.519	1.17
0.00571	8.9877	0.305	85.644	34.384	1.17
0.00661	11.377	0.387	80.393	34.27	1.16
0.00763	13.778	0.468	75.16	34.433	1.17
0.00888	16.164	0.549	69.945	34.505	1.17
0.01087	18.555	0.630	64.751	34.42	1.17
0.01332	20.937	0.711	59.568	34.436	1.17
0.01667	23.302	0.792	54.403	34.435	1.17
0.03446	25.666	0.872	49.275	34.506	1.17
0.2813	29.352	0.997	45.425	34.531	1.17
1.2669	30.17	1.02	40.326	34.498	1.17
2.0283	31.066	1.06	35.23	34.395	1.17
3.3002	31.439	1.07	30.17	34.437	1.17
4.7032	31.559	1.07	25.103	34.379	1.17
6.01	31.664	1.08	19.957	34.328	1.17
11.017	31.865	1.08	14.947	34.25	1.16
16.227	32.006	1.09	9.9916	34.208	1.16
21.354	32.158	1.09	3.9489	34.087	1.16
26.493	32.308	1.10	2.7977	34.027	1.16
31.598	32.393	1.10	1.7736	33.903	1.15
36.708	32.558	1.11	1.1672	33.742	1.15
41.788	32.645	1.11	0.79475	33.593	1.14
46.88	32.773	1.11	0.23174	33.001	1.12
51.951	32.931	1.12			
57.037	33.032	1.12			
62.099	33.196	1.13			
67.173	33.28	1.13			
72.223	33.427	1.14			
77.302	33.585	1.14			

82.358	33.857	1.15
87.427	34.186	1.16
92.459	34.474	1.17
97.51	34.68	1.18

Table S9. CH_4 adsorption/desorption data at 112 K.

	Adsorption			Desorption	
P / kPa	V / mL(STP)	n_a / mol mol^{-1}	P / kPa	V / mL(STP)	n_a / mol mol^{-1}
0.01815	4.2228	0.143	96.091	42.866	1.46
0.01827	6.6541	0.226	90.626	42.684	1.45
0.01844	9.0993	0.309	85.133	42.684	1.45
0.01871	11.543	0.392	79.654	42.723	1.45
0.01938	14.014	0.476	75.565	42.791	1.45
0.02084	16.461	0.559	70.057	42.694	1.45
0.02348	18.919	0.643	64.657	42.663	1.45
0.02843	21.388	0.727	60.605	42.59	1.45
0.03873	23.834	0.810	55.214	42.616	1.45
0.05348	26.249	0.892	49.803	42.663	1.45
0.09476	28.624	0.972	44.527	42.484	1.44
0.18183	30.847	1.05	39.269	42.191	1.43
0.39738	32.676	1.11	35.295	42.07	1.43
0.89119	34.537	1.17	29.427	42	1.43
1.603	35.578	1.21	23.852	41.9	1.42
2.5105	36.31	1.23	19.693	41.827	1.42
3.6776	36.816	1.25	14.373	41.635	1.41
4.9694	37.297	1.27	9.0677	41.336	1.40
6.3696	37.568	1.28	4.0062	40.767	1.38
11.242	38.157	1.30	2.8302	40.56	1.38
16.466	38.522	1.31	1.7951	40.257	1.37
21.673	38.789	1.32	1.2341	39.829	1.35
26.716	39.041	1.33	0.89322	39.466	1.34
31.802	39.282	1.33	0.21516	37.638	1.28
36.877	39.523	1.34			
41.953	39.768	1.35			
47.022	39.974	1.36			
52.088	40.198	1.37			
57.153	40.427	1.37			
62.223	40.645	1.38			

67.292	40.864	1.39
72.348	41.126	1.40
77.401	41.376	1.41
82.466	41.629	1.41
87.519	41.865	1.42
92.578	42.187	1.43
97.516	43.005	1.46

S9. CO_2 adsorption and the corresponding isosteric heat of adsorption (Q_{st}) of 1

CO_2 adsorption and desorption isotherms of **1** measured at 195, 216, 273, and 298 K are shown in **Figure S16.** The temperature during the CO_2 adsorption measurements was controlled using a Bell cryostat. The isosteric heat of adsorption (Q_{st}) was calculated using the Clausius–Clapeyron equation based on adsorption isotherms measured at two temperatures:

$$Qst = \frac{RT_1T_2}{T_2 - T_1}\ln(\frac{P_2}{P_1})$$

where R is the gas constant, and P_1 and P_2 are the equilibrium pressures corresponding to the same adsorption amount at temperatures T_1 and T_2, respectively. The Q_{st} was estimated using the Clausius–Clapeyron equation from the adsorption isotherms measured at two temperature pairs (195/216 K and 273/298 K). The calculated Q_{st} values as a function of CO_2 uptake are shown in **Figure S17**.

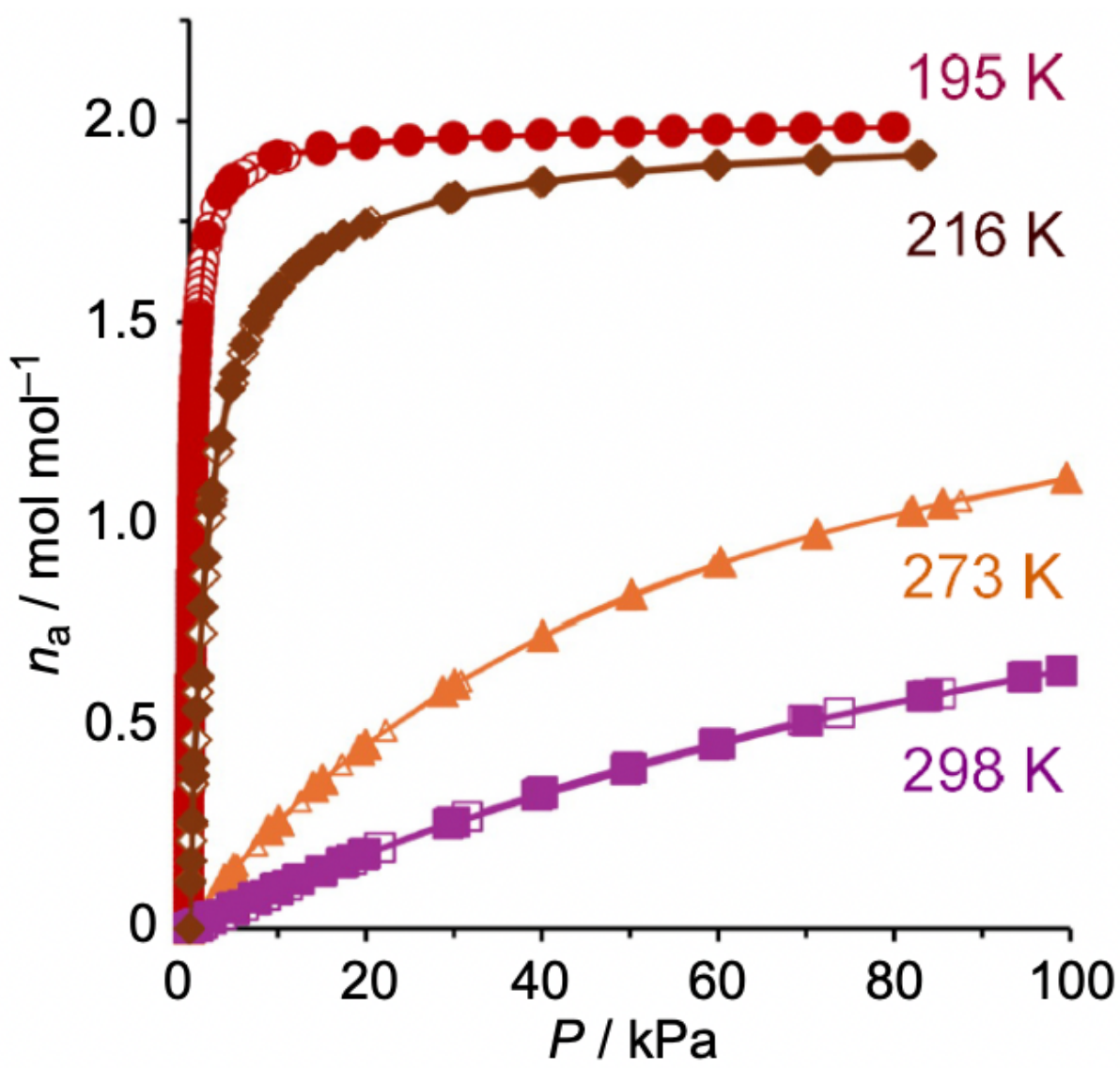

Figure S16. CO_2 adsorption/desorption isotherms of **1** measured at 195, 216, 273, and 298 K.

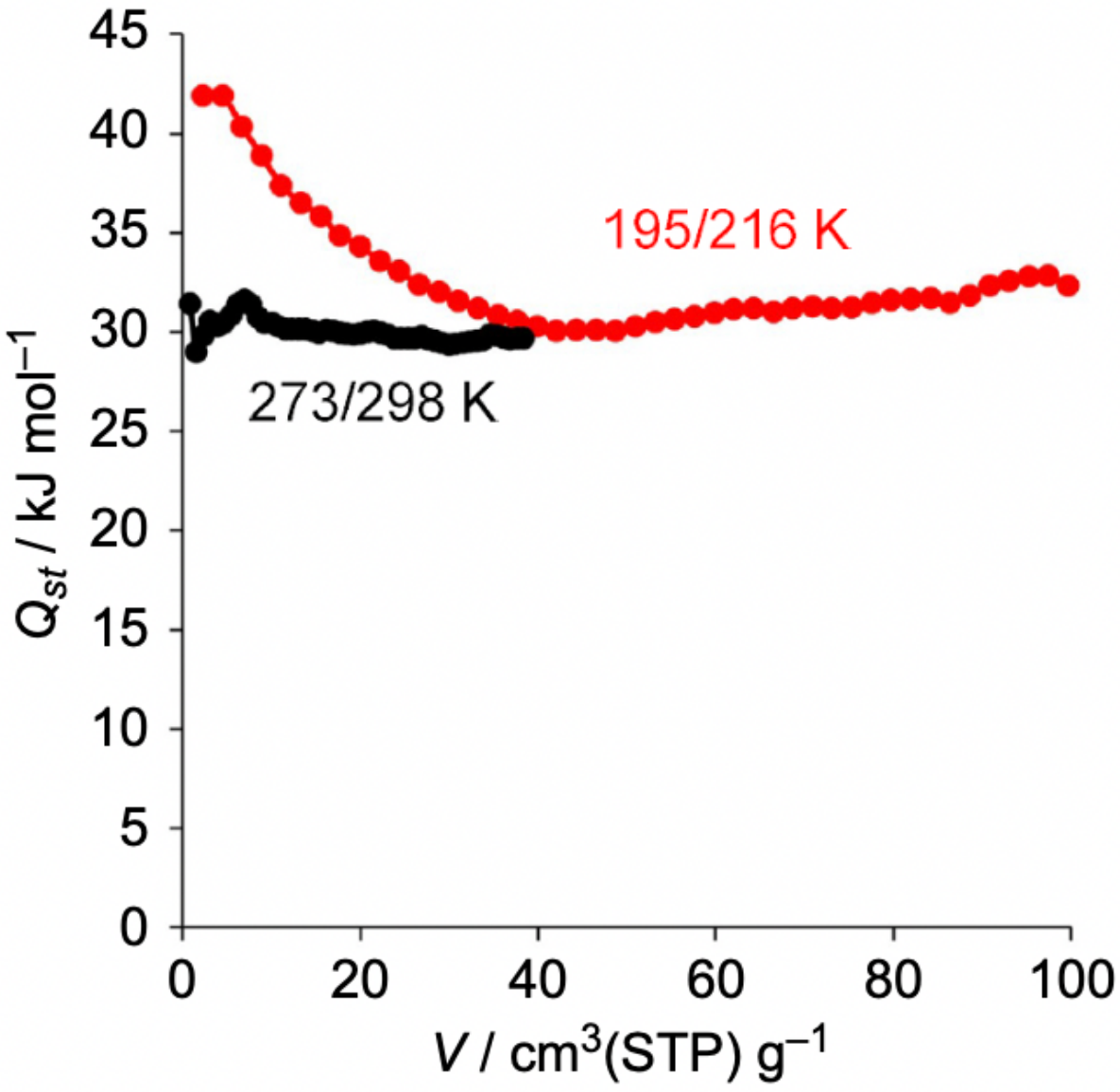

Figure S17. Isosteric heat of adsorption (Q_{st}) for CO_2 calculated using the Clausius–Clapeyron equation from adsorption isotherms measured at 195/216 K and 273/298 K.

S10. pXRD under a CO_2 atmosphere

Powder X-ray diffraction (pXRD) measurements in this section were performed by Rigaku Co. using a Rigaku SmartLab diffractometer (Cu$K\alpha$, λ = 1.541862 Å). pXRD measurements were conducted under CO_2 according to the following procedure. The temperature was changed at a rate of 10 °C min^{-1}, and each target temperature was held for 3 min. CO_2 was introduced at a flow rate of 400 mL min^{-1}, and pXRD patterns were recorded while varying both the atmosphere and the temperature.

(1) Experiment-1

In Experiment-1, the sample was first measured under vacuum at 273 K. The atmosphere was then switched to CO_2 at the same temperature. Subsequently, the measurement was carried out at 195 K under CO_2, followed by a final measurement at 298 K under CO_2.

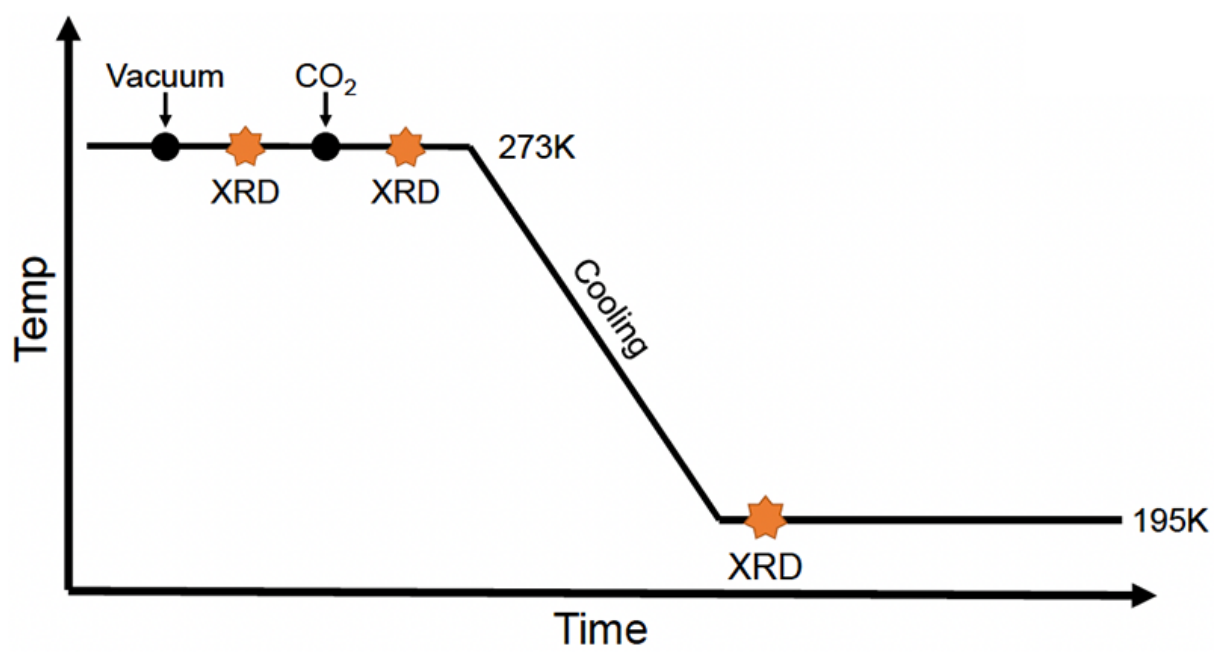

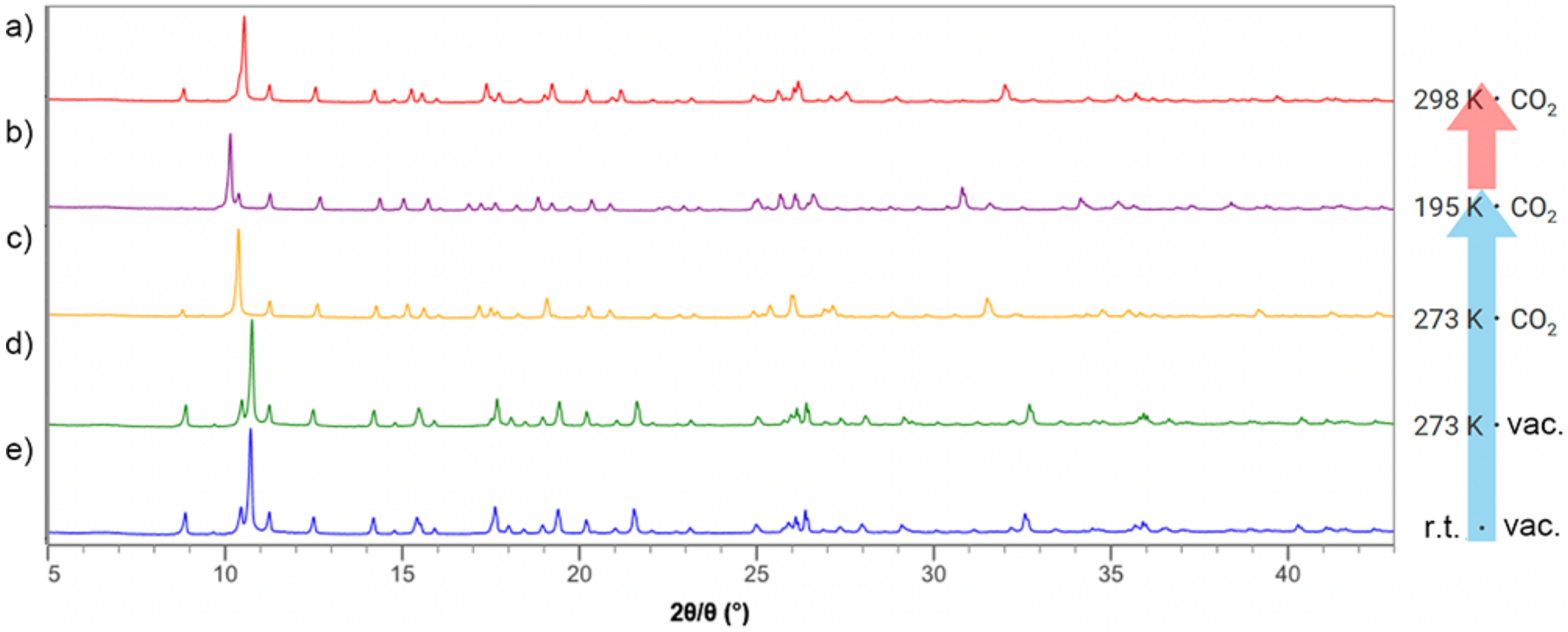

Figure S18. pXRD patterns of **1** under vacuum and CO_2: a) under CO_2 at 298 K, b) under CO_2 at 195 K, c) under CO_2 at 273 K, d) under vacuum at 273 K, and e) empty sample under vacuum at room temperature.

(2) Experiment-2

In Experiment-2, measurements were first conducted under vacuum at 273 K and then under vacuum at 195 K. The atmosphere was subsequently changed to CO_2 at 195 K, and a final measurement was performed at 298 K under CO_2.

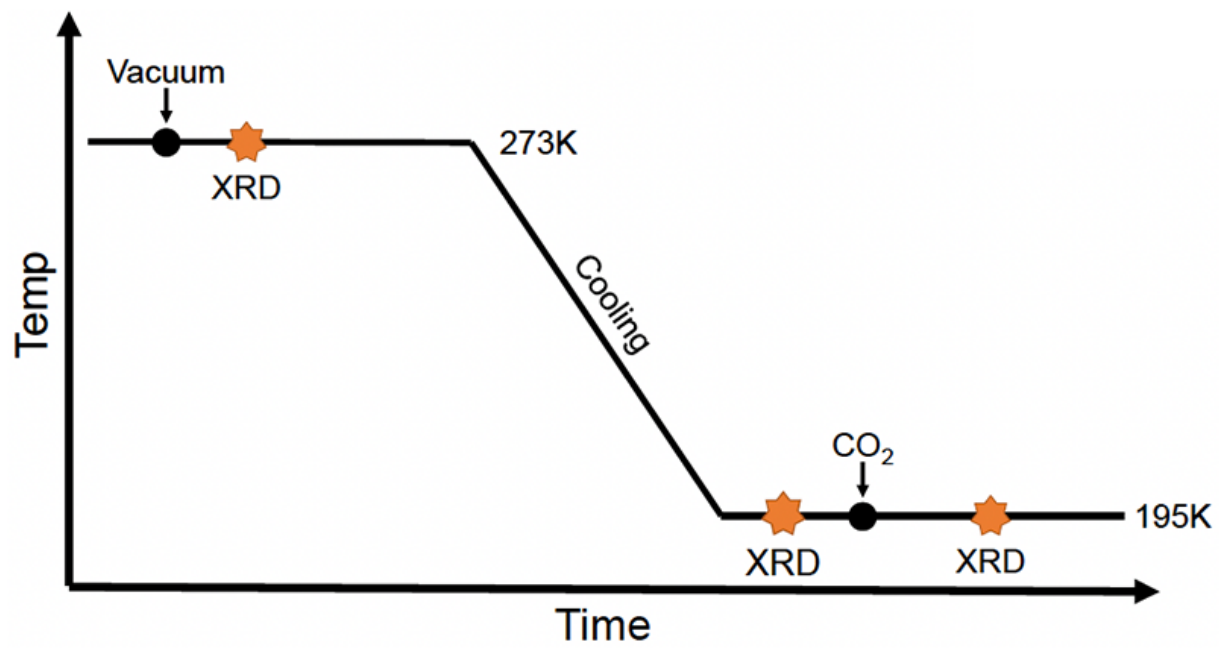

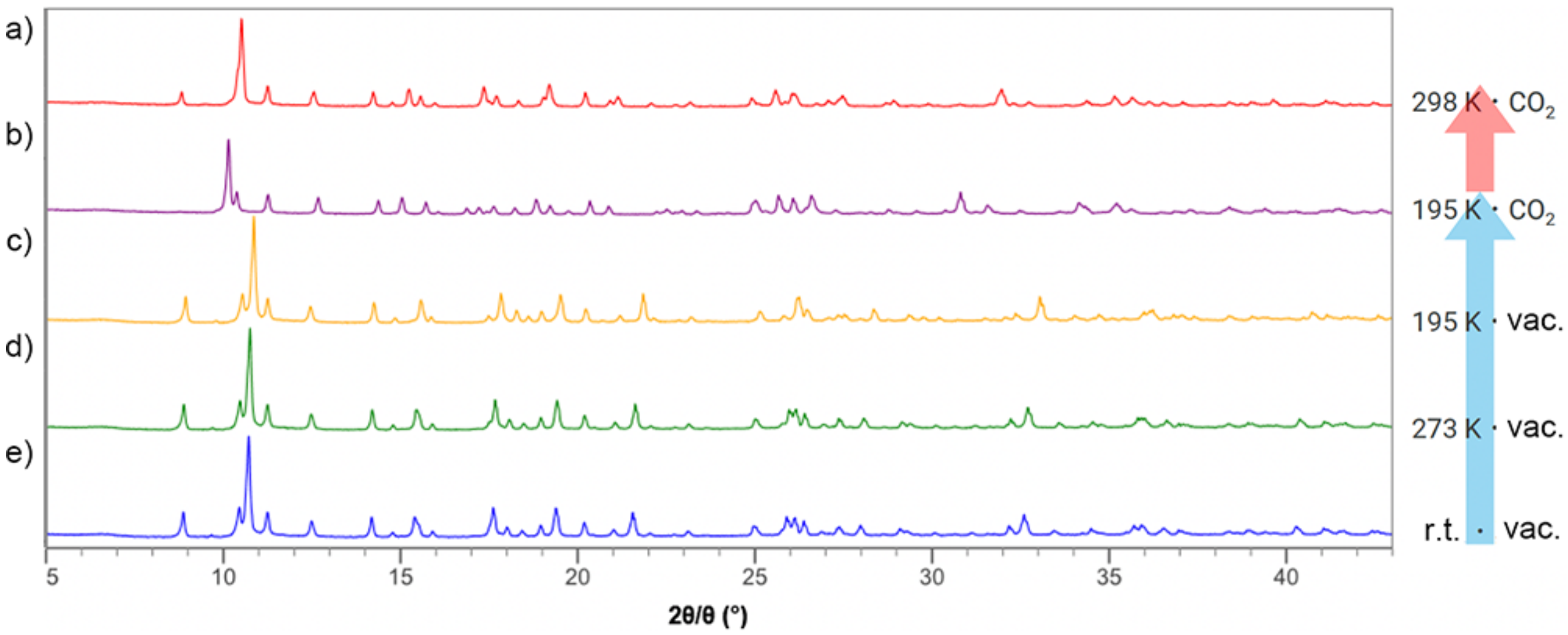

Figure S19. pXRD patterns of powdered sample **1**: a) under CO_2 at 298 K, b) under CO_2 at 195 K, c) under vacuum at 195 K, d) under vacuum at 273 K, and e) empty sample under vacuum at room temperature.

S11. Comparison with reported porous framework materials

The CO_2 uptake of **1** at 273 K was compared with previously reported MOFs and COFs [ref. S3].

Table S10. The CO_2 adsorption parameters of **1** at 273 K with parts of reported MOFs and COFs.

Materials	m^2 g^{-1}	mmol g^{-1}	mmol m^{-2}	Materials	m^2 g^{-1}	mmol g^{-1}	mmol m^{-2}
**1** (This work)	100	1.45	0.0145				
MOF							
MOF-2	708	1.20	0.00169	UIO-67-MIMS$_{(0.25)}$	1627	2.96	0.00182
S-MOF-2	1176	4	0.00340	UIO-67-MIMS$_{(0.50)}$	933	1.33	0.00143
UiO-66(Zr$_{100}$)	1390	2.1	0.00151	UIO-67-MIMS$_{(0.75)}$	601	0.91	0.00151
NU-1000-PhTz	1560	2.2	0.00141	UIO-67-MIMS	325	1.92	0.00591
UIO-67	2407	1.11	0.00046				
COF							
TPT/OH-COF	424	0.90	0.00212	SJTU-COF-Br	944	4.55	0.00482
TPE-COF-I	1535	3.06	0.00199	SJTU-COF-Cl	954	3.34	0.00350
TPE-COF-II	2168	5.3	0.00244	SJTU-COF-AcO	865	3.89	0.00450
T-COF	587	4.04	0.00688	SJTU-COF-CF_3SO_3	879	3.16	0.00359
3D-CageCOF-1	1040	4.63	0.00445	[HO_2C]$_{25\%}$-H_2P-COFs	786	2.18	0.00277
SNW-1	794	2.6	0.00327	[HO_2C]$_{50\%}$-H_2P-COFs	673	3.05	0.00453
3D-HNU5	864	2.8	0.00324	[HO_2C]$_{75\%}$-H_2P-COFs	482	3.57	0.00741
TPA-COF	1896	4.03	0.00213	[HO_2C]$_{100\%}$-H_2P-COFs	364	3.95	0.0109
TPA-4C-NH_2	1827	5.26	0.00288				

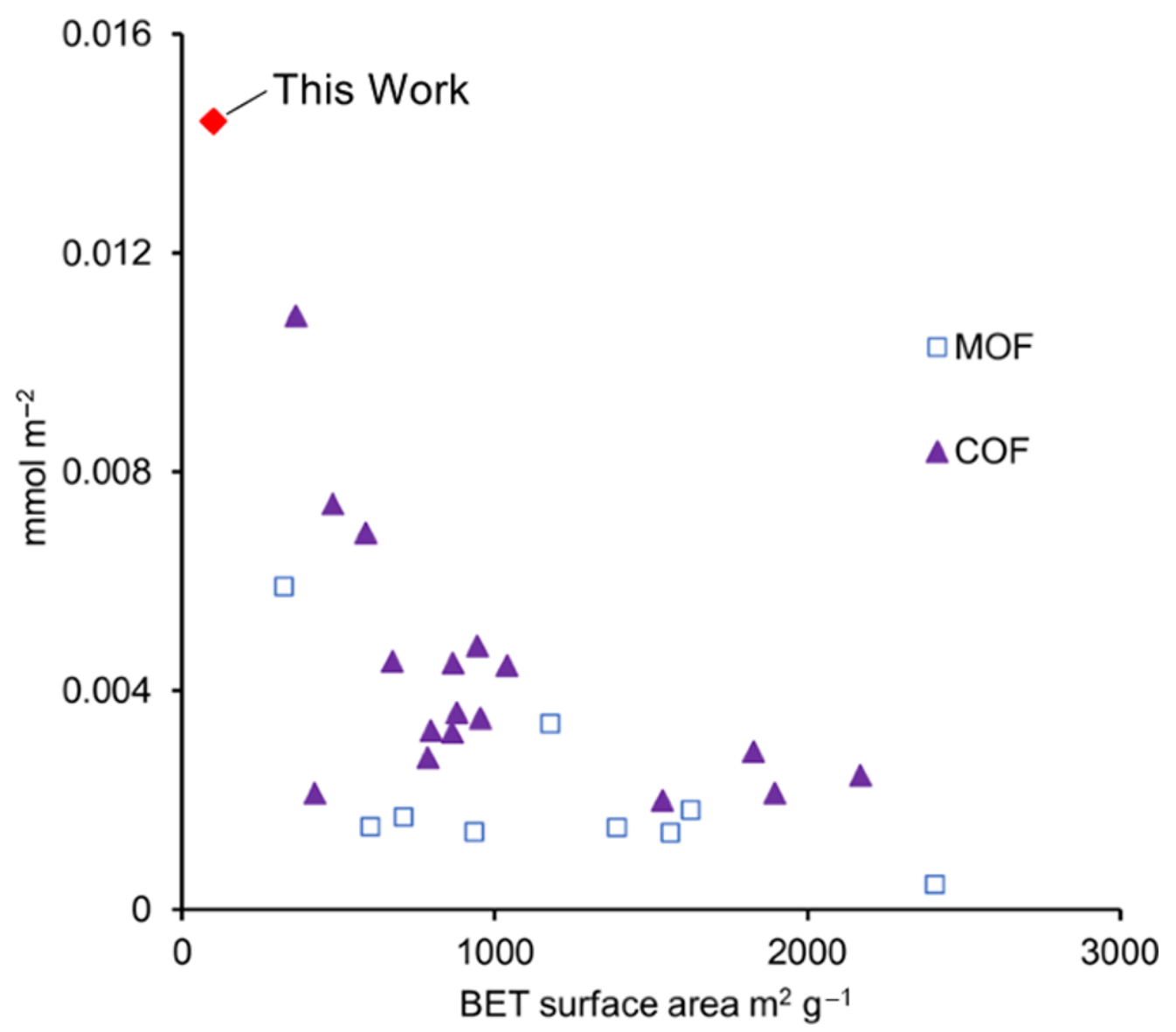

Figure S20. BET surface area (m^2 g^{-1}) vs. CO_2 uptake per surface area (mmol m^{-2}) for **1** and reported MOFs/COFs.

Table S11. The CO_2 uptake of **1** at 298 K was compared with reported MOFs and COFs.

Materials	$m^2\ g^{-1}$	mmol g^{-1}	mmol m^{-2}
**1** (This work)	100	0.83	0.0083
MOF			
ZIF-8	1550	0.88	0.00057
NU-1000	2140	2.80	0.00131
NU-1000-PhTz	1560	1.40	0.00090
UiO-67	2407	1.11	0.00046
UiO-66(Zr)	1390	2.10	0.00151
COF			
TPE-COF-I	1535	1.68	0.00109
TPE-COF-II	2168	2.70	0.00125
T-COF	587	2.66	0.00453
SNW-1	794	1.84	0.00232
3D-CageCOF-1	1040	2.43	0.00234

To compare CO_2 adsorption performance under different conditions, both high-pressure (1 bar; **Table S11**) and low-pressure (400 ppm) uptake values at 298 K were evaluated (**Table S12**). The former reflects the overall adsorption capacity, whereas the latter represents performance under practical conditions.

Table S12. CO_2 uptake at 400 ppm and 298 K compared with benchmark ultramicroporous MOFs.

Materials	$m^2\ g^{-1}$	mmol g^{-1}	mmol m^{-2}	Ref.
**1** (This work)	100	5.5×10^{-4} (estimated)	5.5×10^{-6}	—
SIFSIX-3-Cu	300	1.24	4.13×10^{-3}	ref. S3
SIFSIX-3-Zn	300	0.13	4.3×10^{-4}	ref. S4
KAUST-7 (NbOFFIVE-1-Ni)	280*	~1.3	~4.6×10^{-3}	ref. S5

*CO_2-derived surface area

S12. Breakthrough measurements of CO_2/N_2 and CO_2/CH_4 mixtures

Breakthrough measurements were performed at 303 K using equimolar CO_2/N_2 and CO_2/CH_4 mixtures. A 1.02 g sample of **1** (1.34 mmol based on $C_{26}H_{10}F_8N_2O_8Zn_2$) was packed into the sample tube.

The outlet gas composition was monitored using a BELMass quadrupole mass spectrometer connected to a BELCAT II system (**Figure S21**). For measurements under humid conditions, the feed gas was passed through a water bubbler maintained at 28 °C. The resulting relative water-vapor pressure was $P/P_0 = 0.89$ at the sample temperature of 30 °C. Dry measurements were performed without passing the feed gas through the bubbler.

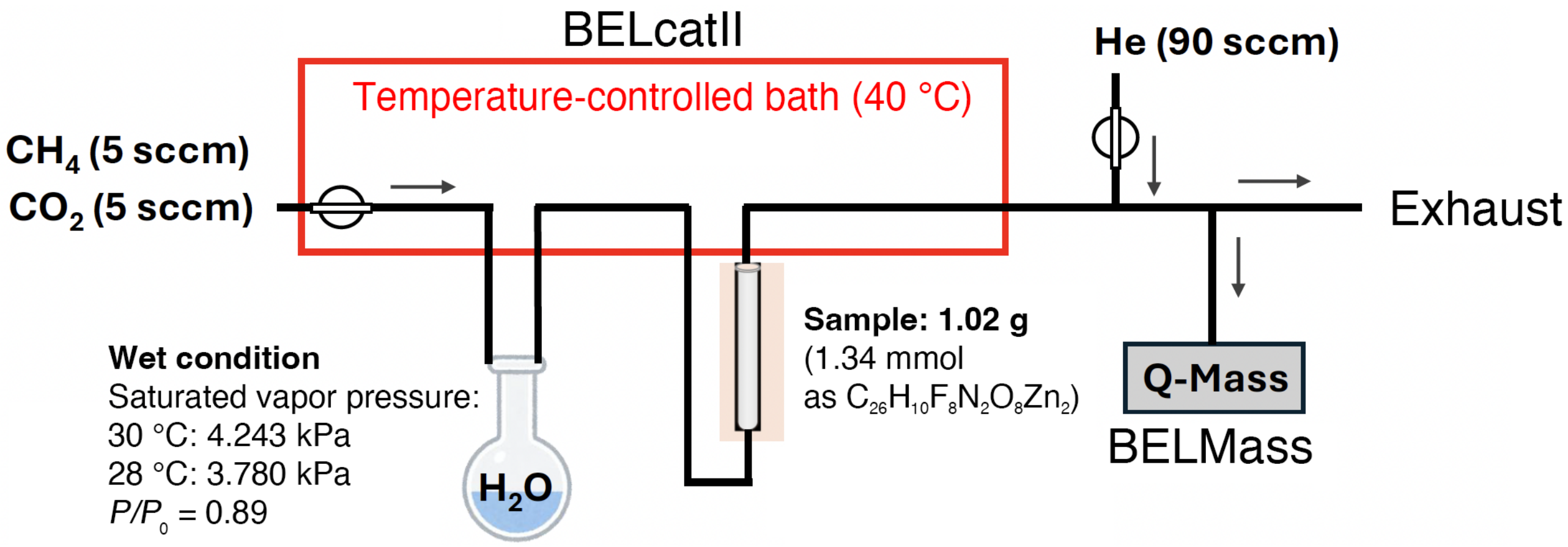

Figure S21. Experimental setup for the breakthrough measurements under humid conditions.

The sample was activated at 100 °C under N_2 flow before the breakthrough measurements. The sample temperature was then adjusted to 303 K. The CO_2/N_2 and CO_2/CH_4 mixtures were introduced at flow rates of 5 sccm for each component, with He used as the carrier gas. For the CO_2/CH_4 measurements, CO_2 and CH_4 were monitored at m/z = 44 and 16, respectively. The contribution of the CO_2 fragment to the m/z = 16 signal was corrected by subtracting 0.109 times the m/z = 44 signal, as determined from a CO_2-only measurement.

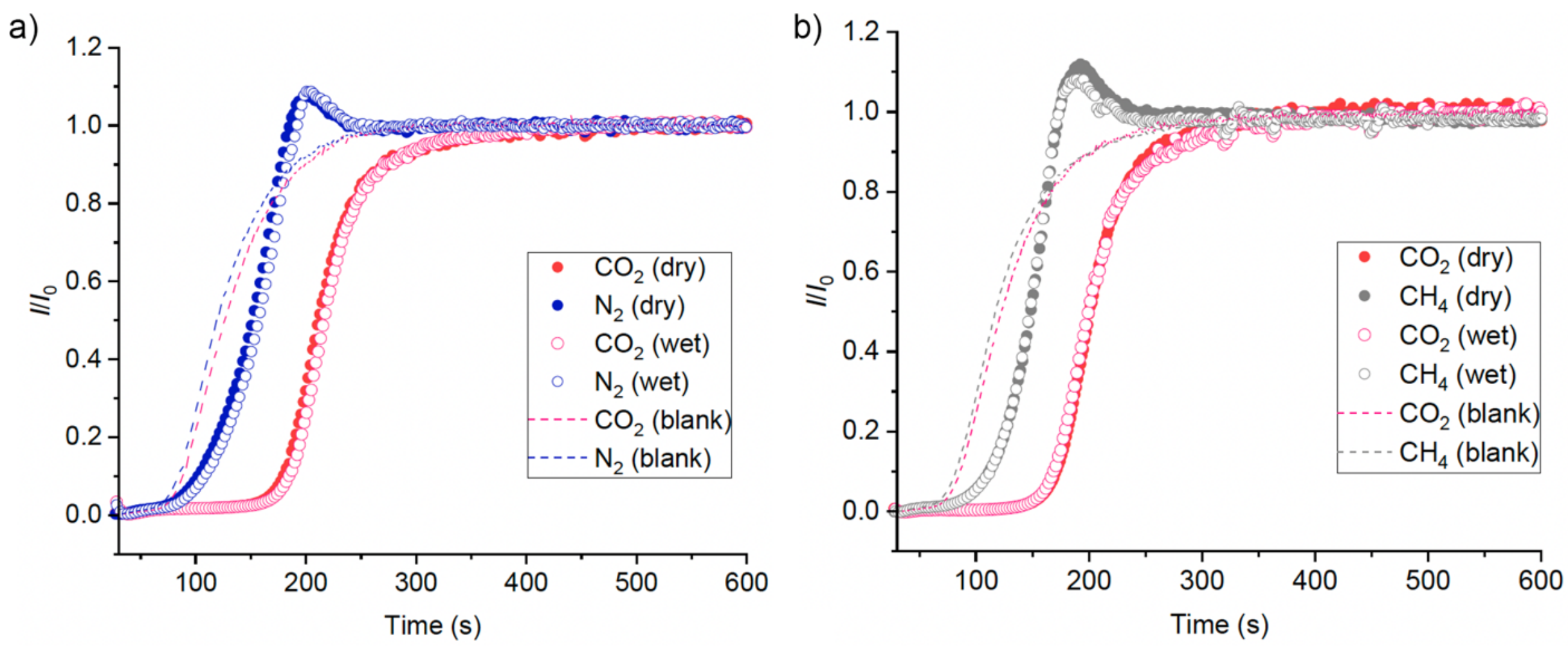

Figure S22. Breakthrough separation of a) CO_2/N_2 and b) CO_2/CH_4 mixtures.

S13. pXRD of guest-soaked crystals

Powder X-ray diffraction (pXRD) measurements described in this section were performed using a Bruker D8 Quest diffractometer equipped with Mo*K*α radiation (λ = 0.71073 Å). All measurements were conducted using the capillary method. A powdered sample of **1** was packed into a glass capillary and immersed for 1 min in a 1:1 molar mixture of C_6H_6 and C_6F_6. After soaking, the capillary was sealed with wax to prevent solvent evaporation. The resulting sample was then subjected to pXRD analysis.

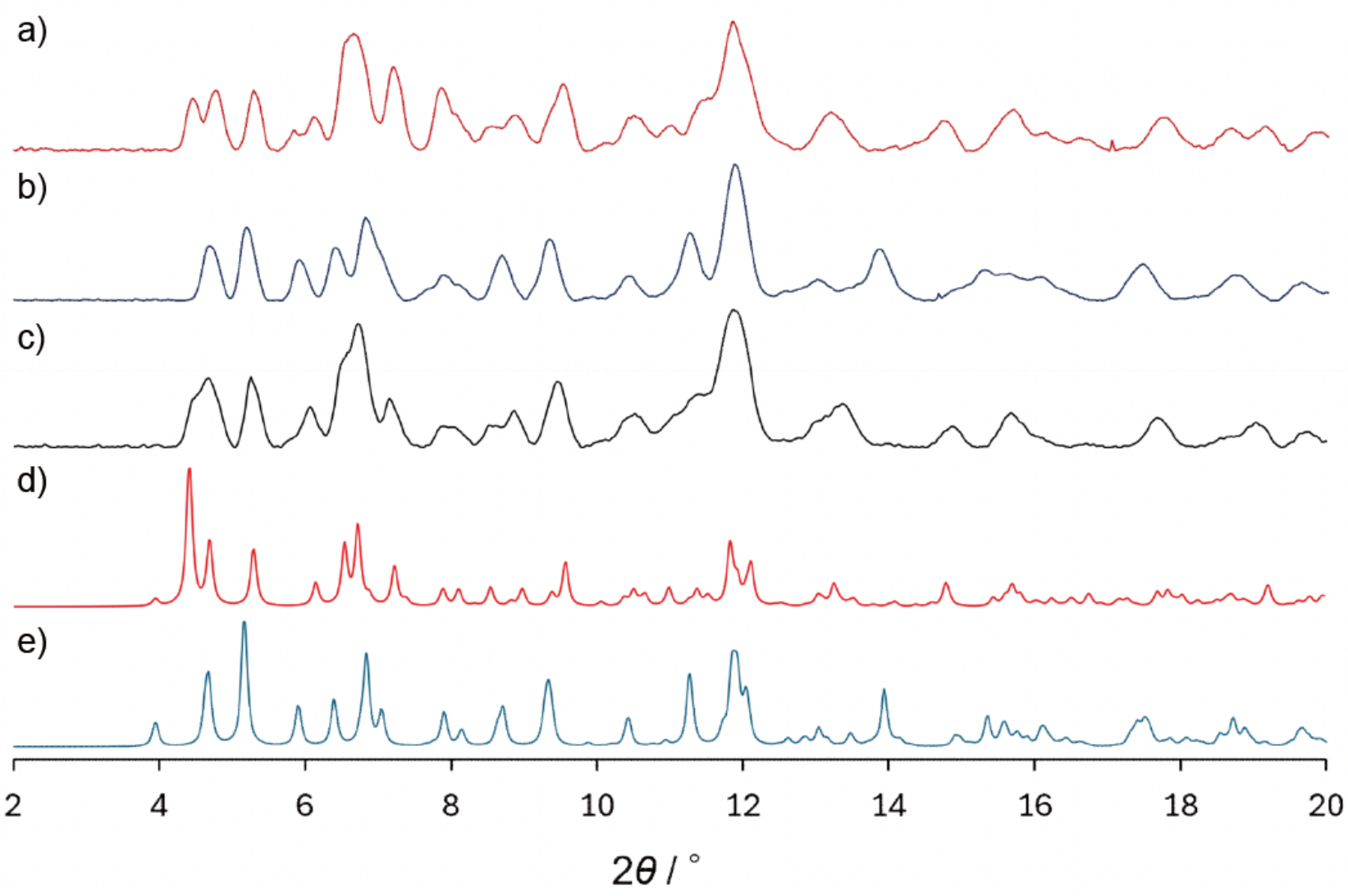

Figure S23. pXRD patterns obtained using the capillary method: a) **1** with C_6H_6, b) **1** with C_6F_6, c) **1** with 1:1 mixture of C_6H_6 and C_6F_6; simulations of SC-XRD of d) **1**•C_6H_6 and e) **1**•C_6F_6.

S14. NMR spectra of guest-inserted 1 in a C_6H_6-C_6F_6 (1:1) mixture

Empty single crystals of **1** were immersed in a C_6H_6-C_6F_6 (1:1) solution for 10 min, followed by dissolution of the guest-inserted crystals in DMSO-d_6. The uptake ratio was determined by integrating the aromatic protons of C_6H_6 relative to the pyridyl protons of **1**. Across five consecutive wetting–drying cycles (Cycles 1–5), the benzene uptake remained nearly constant (ca. 97% relative to **1**•C_6H_6 = 100%), demonstrating the excellent reusability of **1**. No signals attributable to C_6F_6 inclusion were observed.

(1) ^{1}H NMR of the empty crystal of **1** dissolved in DMSO-d_6: pyridine (C_5H_5N): δ 7.52-7.49 (t, J = 12 Hz, 2H), 7.93-7.89 (t, J = 16 Hz, 1H), 8.64-8.63 (d, J = 4 Hz, 2H). ^{13}C NMR of pyridine (C_5H_5N): δ 149.9, 138.2, 125.0 and tetrafluoroisophthalic acid ($C_8H_2F_4O_4$): δ 163.5.

^{1}H NMR

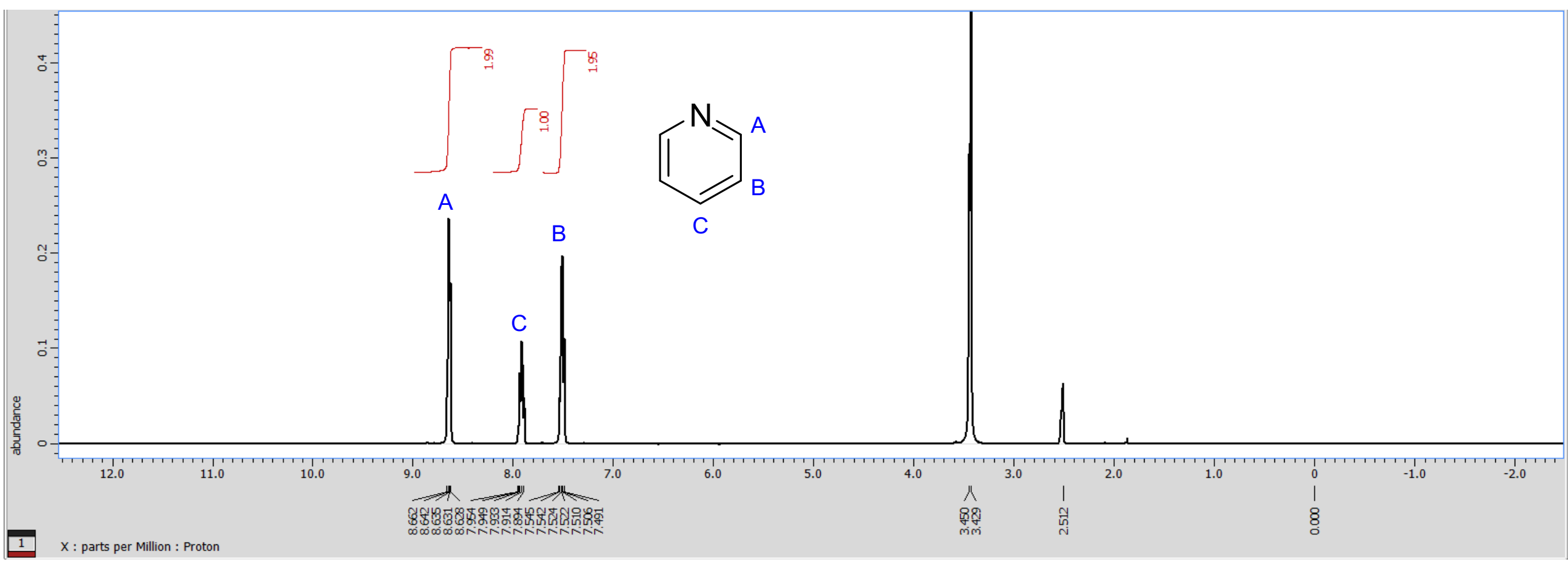

^{13}C NMR

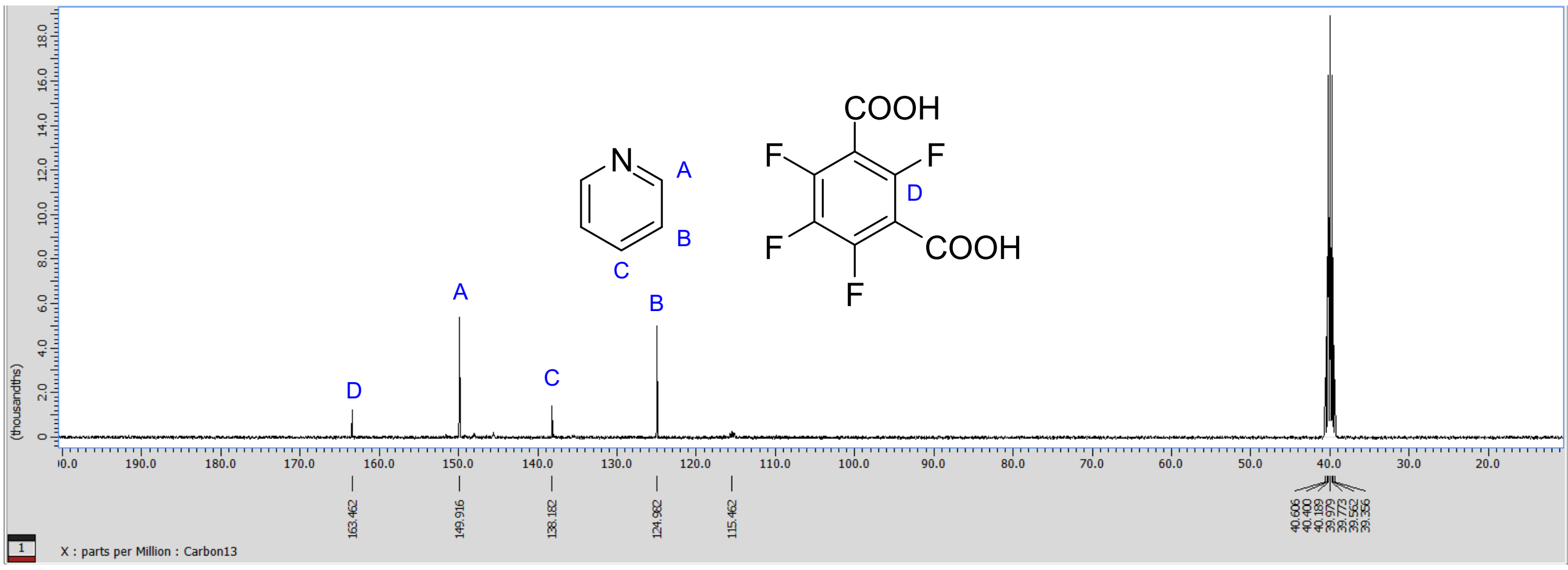

Figure S24. ^{1}H and ^{13}C NMR spectra of **1**.

(2) ^{1}H NMR of benzene-inserted crystal of **1**, dissolved in DMSO-d_6.

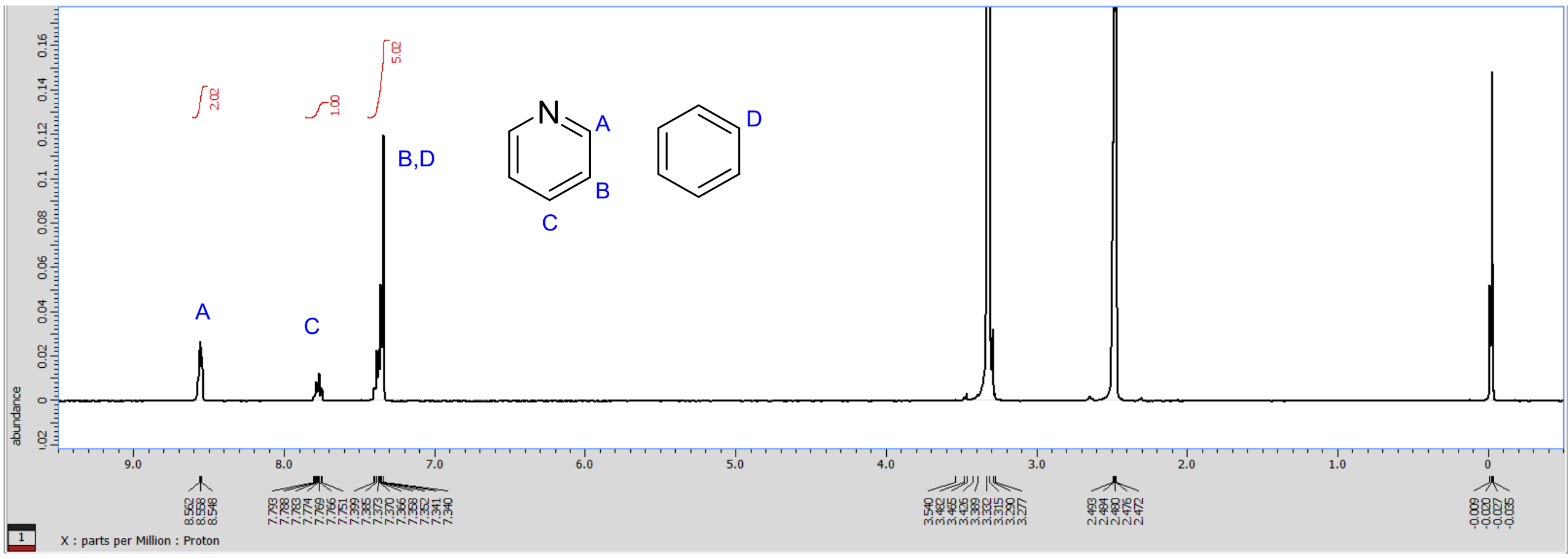

Figure S25. ^{1}H NMR spectra of **1** after immersion in C_6H_6.

(3) Repeated benzene-insertion, monitored by ^{1}H NMR (DMSO-d_6).

Cycles-1

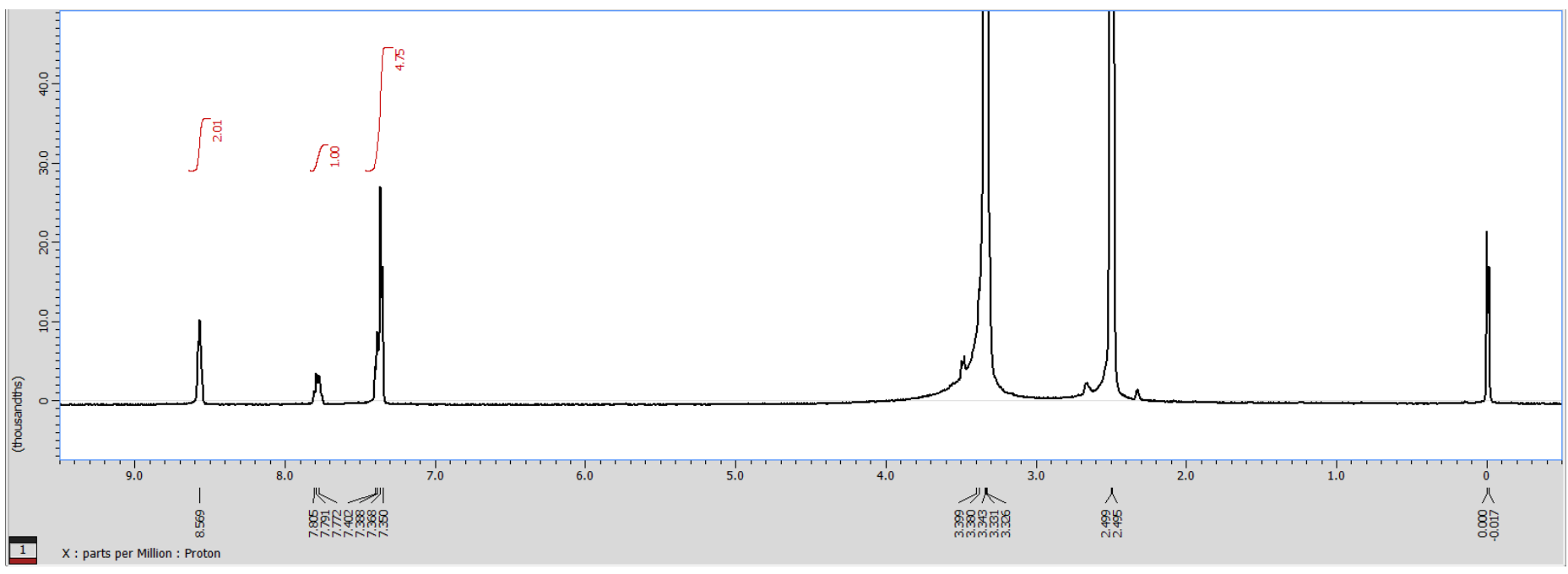

Cycles-2

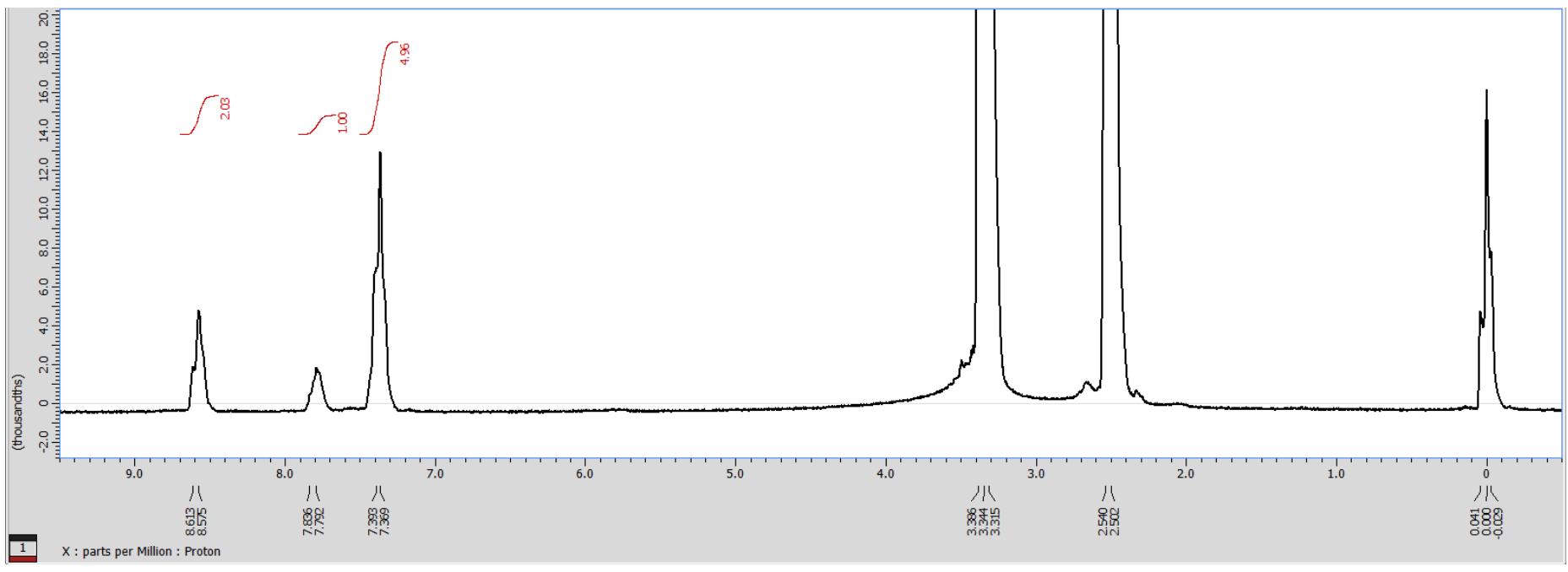

Cycles-3

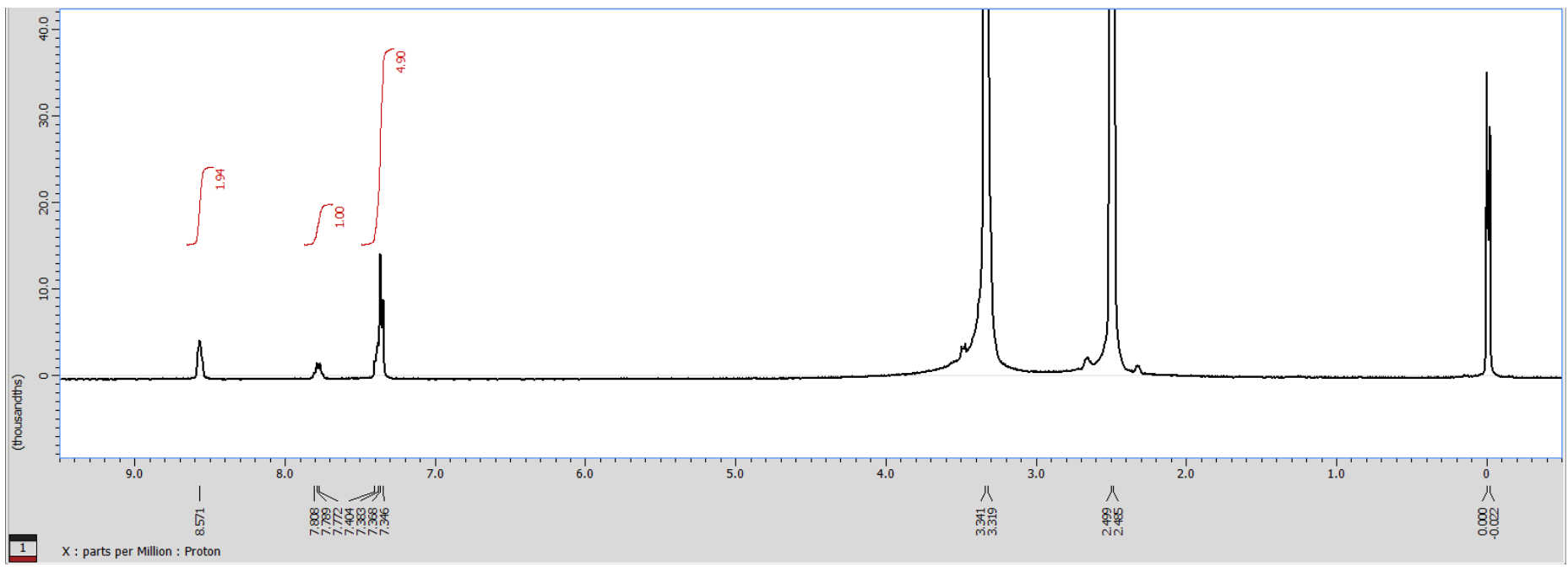

Cycles-4

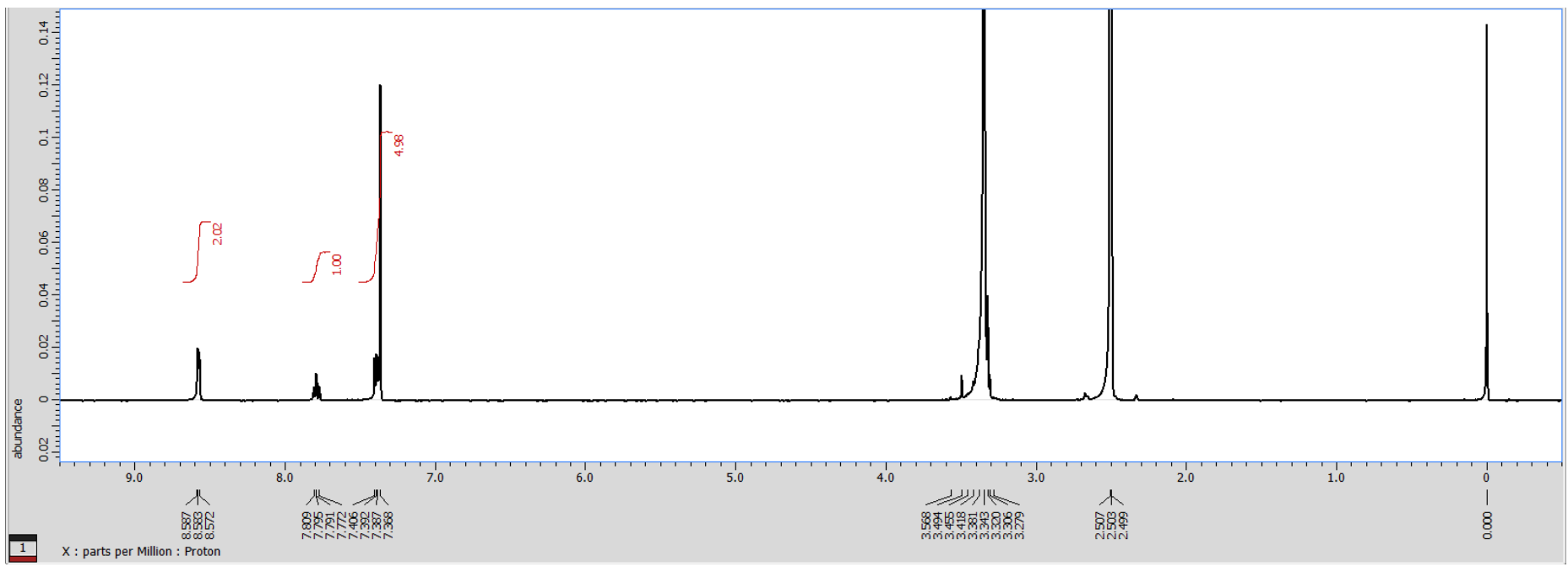

Cycles-5

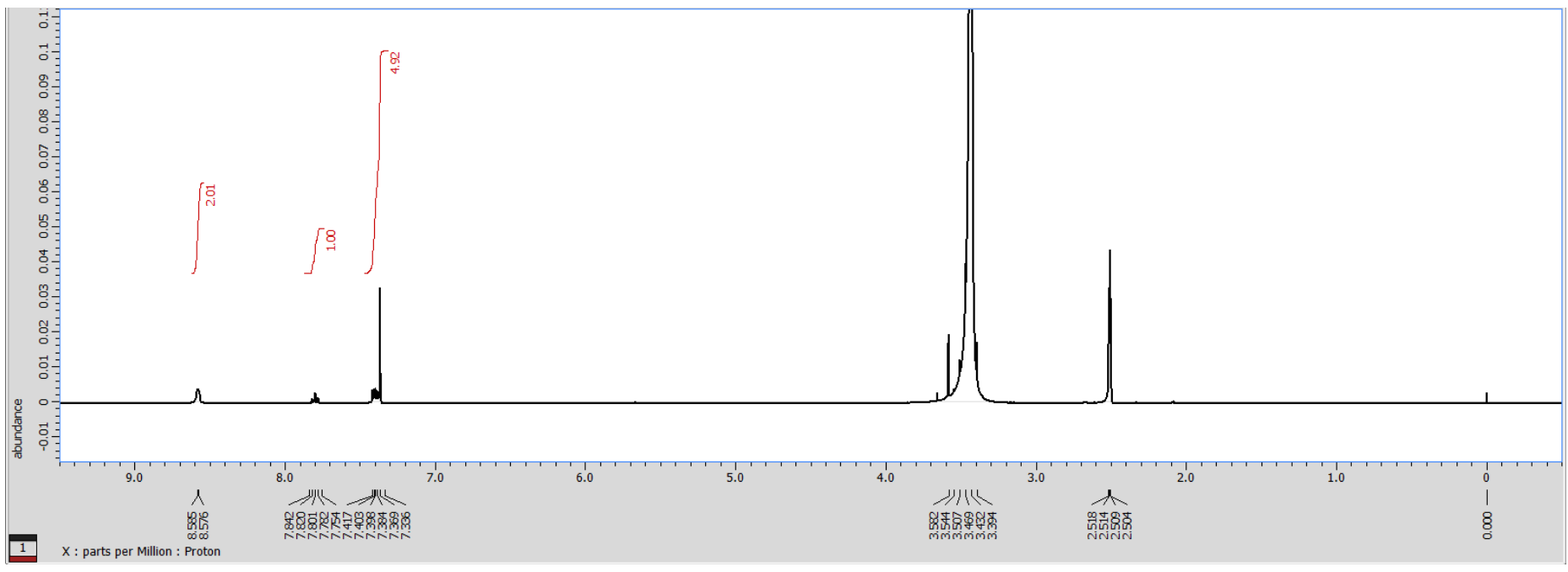

Figure S26. ^{1}H NMR spectra of **1** after immersion in a C_6H_6-C_6F_6 (1:1) mixture (Cycles 1-5).

(4) ^{13}C NMR of benzene-inserted crystal of **1**, dissolved in DMSO-d_6.

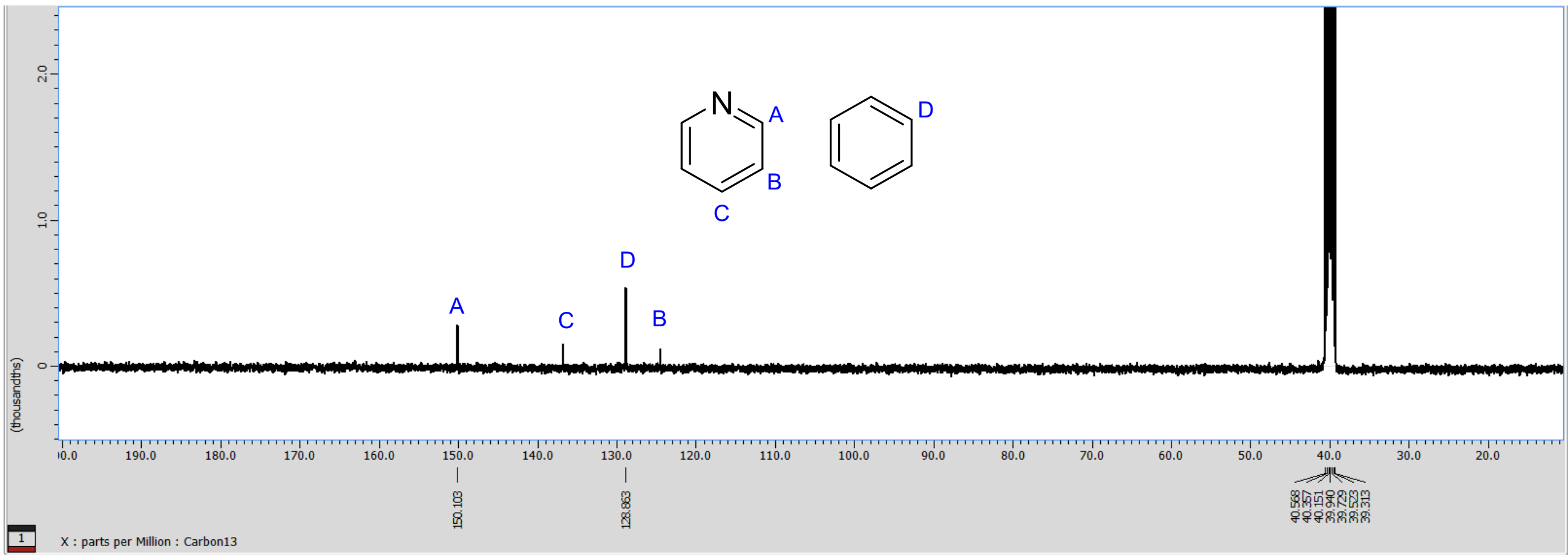

Figure S27. ^{13}C NMR spectra of **1** after immersion in a C_6H_6-C_6F_6 (1:1) mixture.

S15. Competitive guest uptake determined by ^1H NMR

Competitive uptake experiments were performed by immersing empty crystals of **1** in equimolar binary mixtures consisting of C_6H_6 and one competing guest: $C_6H_3F_3$, 1,3- and 1,4-cyclohexadienes (1:1 mixture), cyclohexene, cyclohexane, and *n*-hexane. After 10 min of exposure, the crystals were dissolved in DMSO-d_6, and the guest ratio was determined by integrating the aromatic or aliphatic proton signals relative to the pyridyl protons of **1**.

benzene and $C_6H_3F_3$

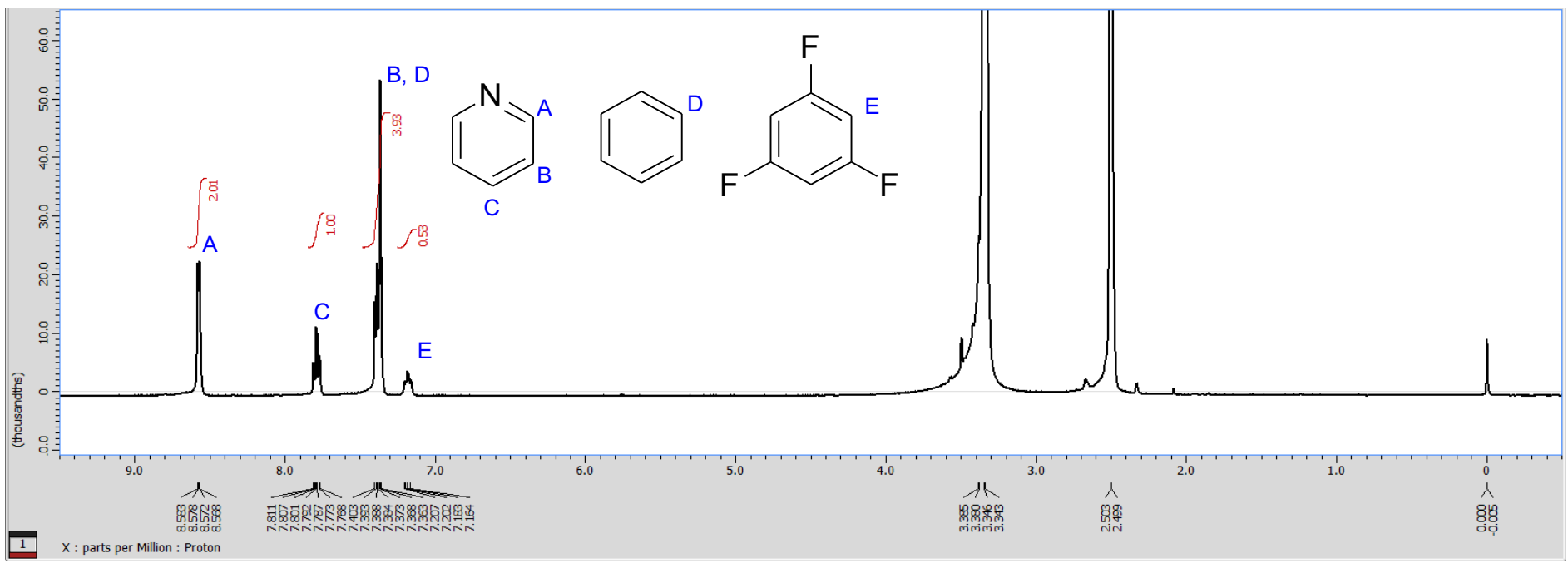

benzene and cyclo-hexadienes

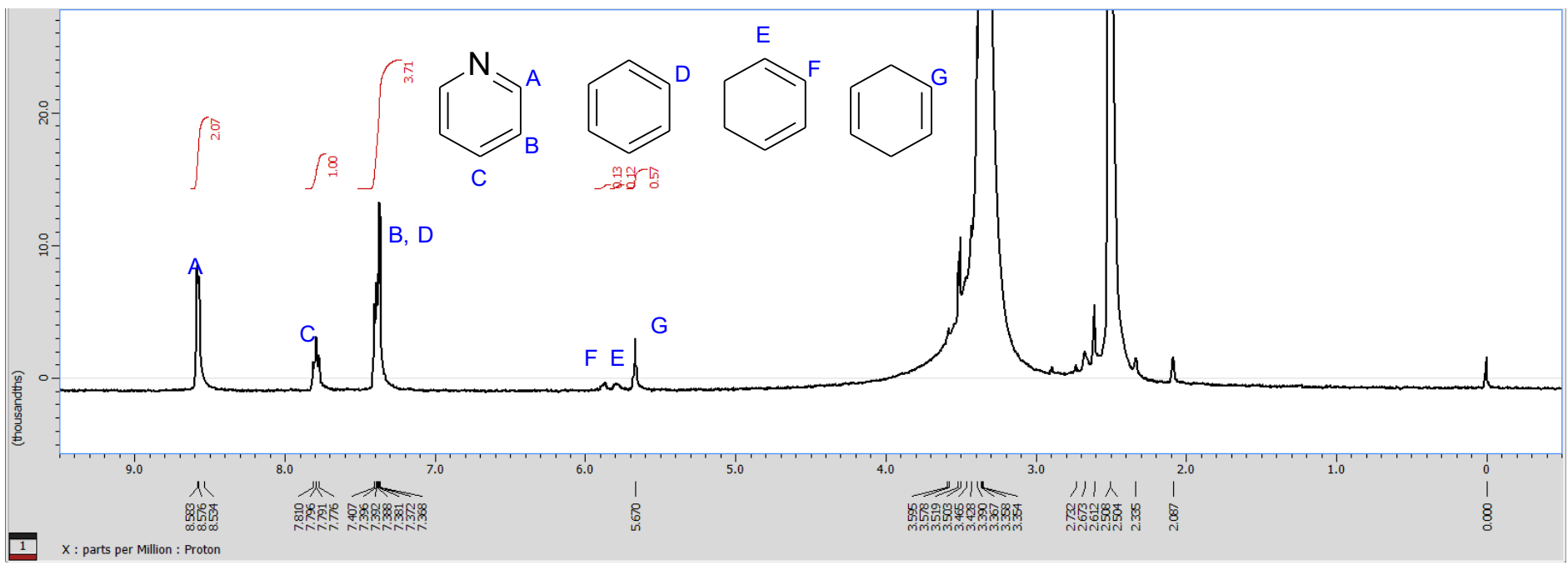

benzene and cyclohexene

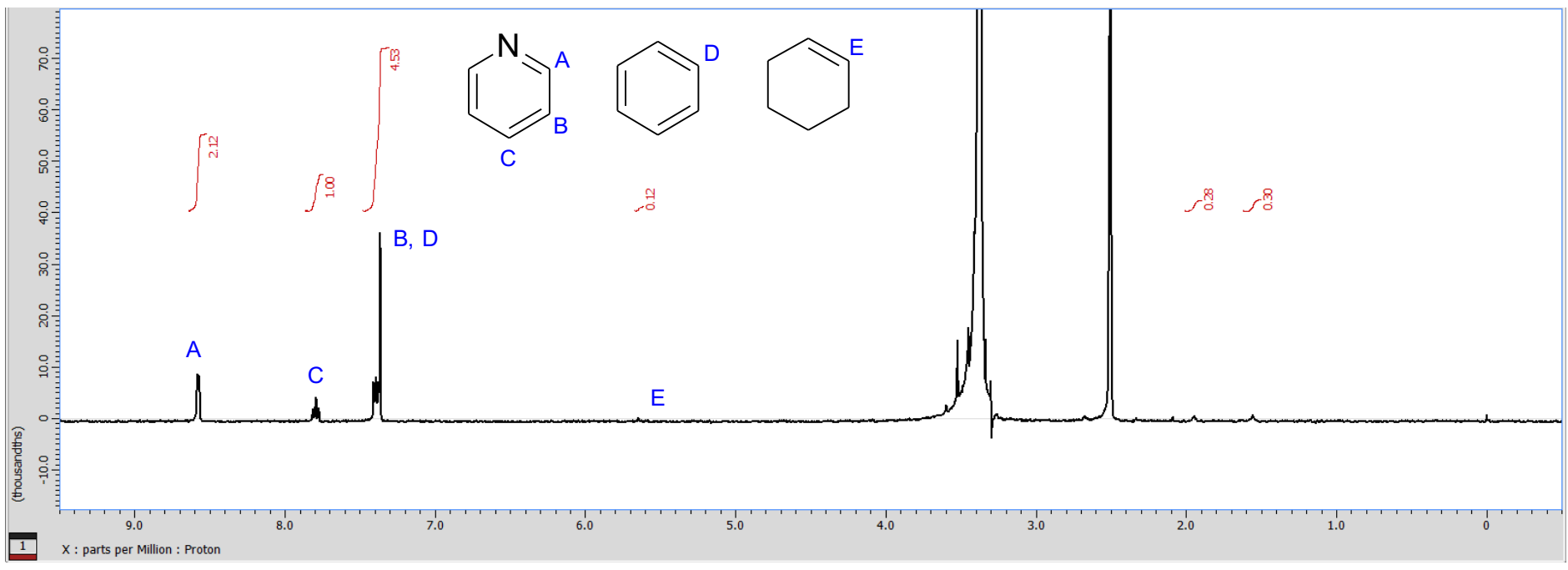

benzene and cyclohexane

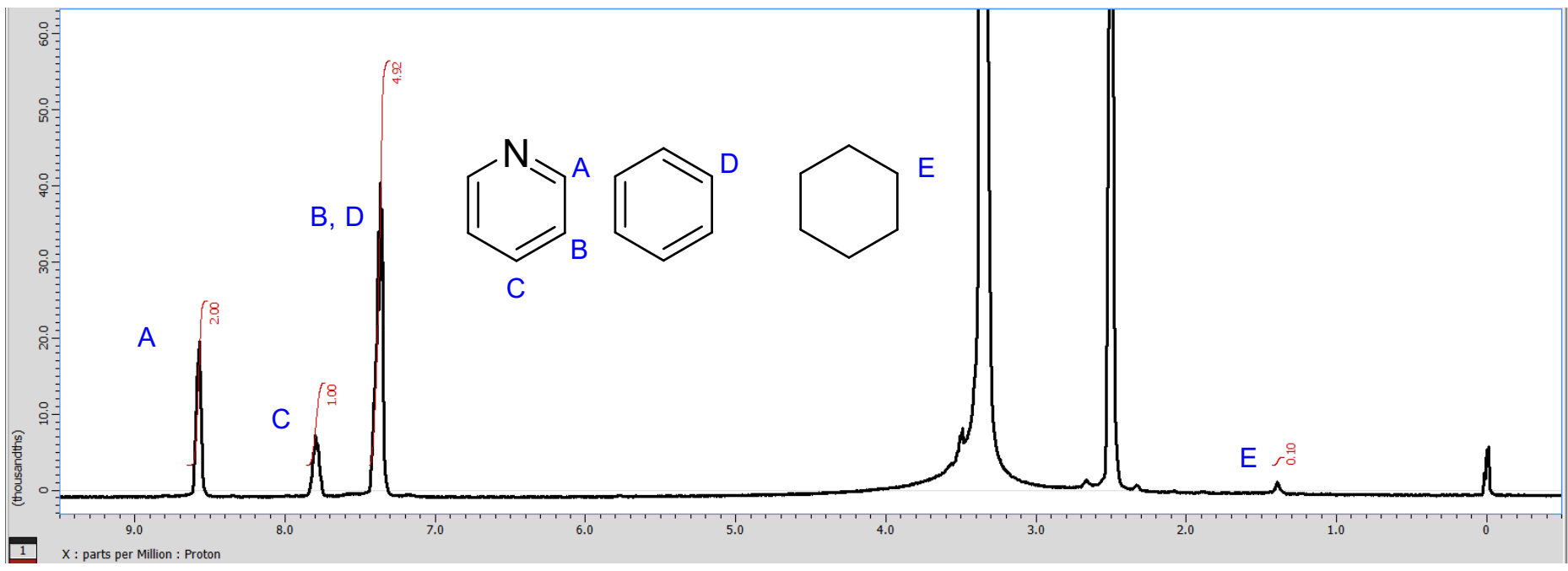

benzene and *n*-hexane

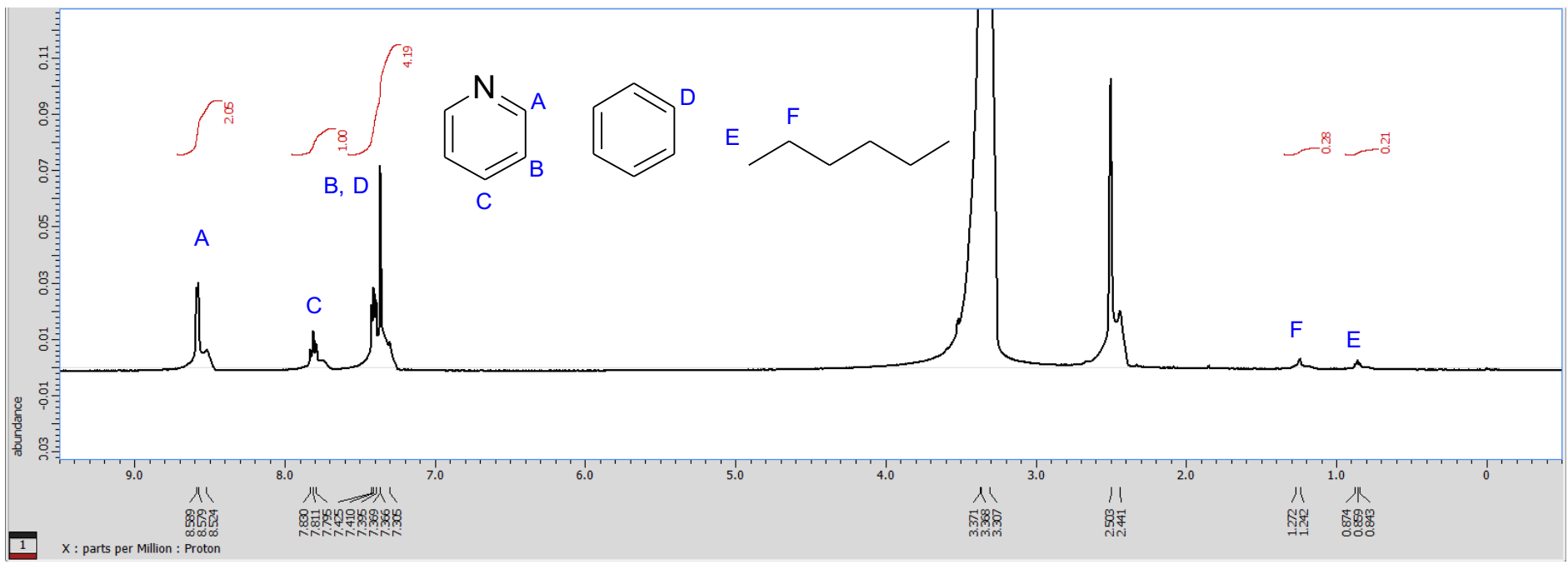

Figure S28. ^{1}H NMR spectra of **1** after immersion in 1:1 mixture of C_6H_6 and guest ($C_6H_3F_3$, cyclohexadienes, cyclohexene, cyclohexane, *n*-hexane).

In addition to the equimolar mixture (5:5, **Figure S28**), competitive uptake experiments were performed using benzene/cyclohexene and benzene/cyclohexane mixtures with additional feed compositions of 3:7, 1:9, and 0:10 (**Table S13**, **Figures S29** and **S30**).

Table S13. Competitive uptake ratios from benzene/cyclohexane and benzene/cyclohexene mixtures.

Feed ratio	C_6H_6/C_6H_{12} uptake (%)	C_6H_6/C_6H_{10} uptake (%)
5:5	97/2	84/12
3:7	98/3	68/25
1:9	74/1	11/9
0:10	0/0.06	0/10

benzene
and
cyclohexene
3:7

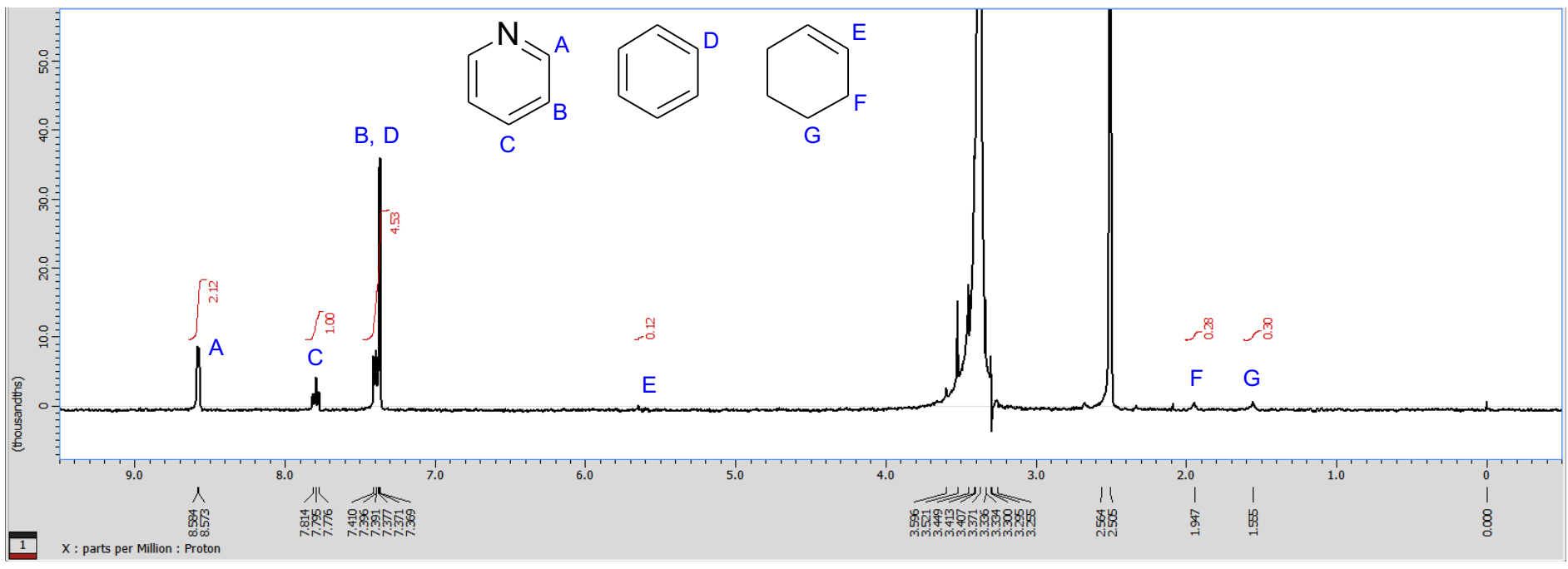

1:9

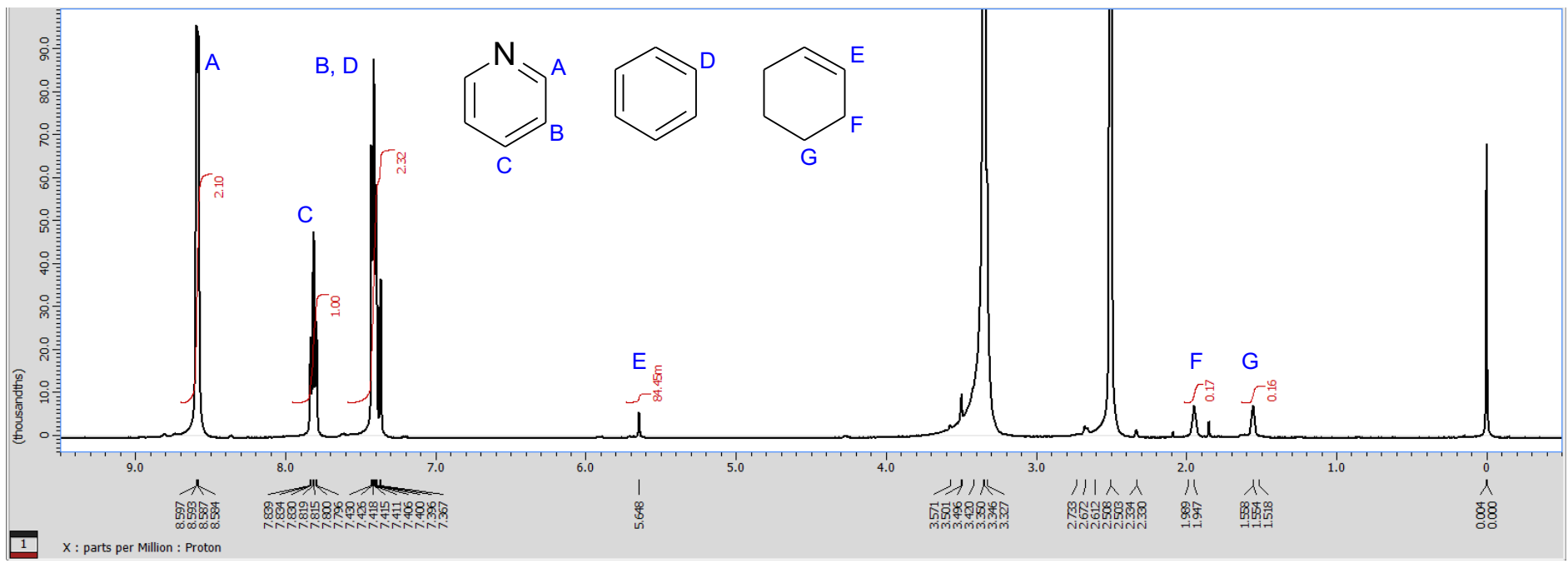

0:10

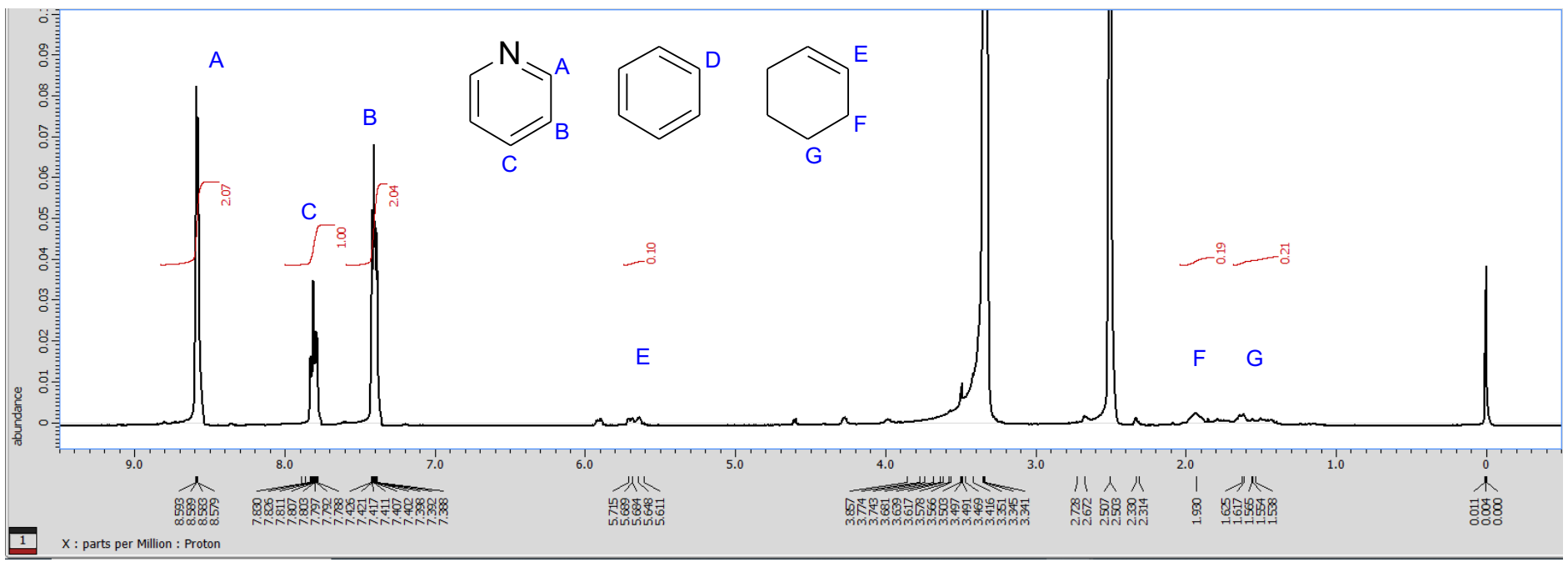

Figure S29. ^{1}H NMR spectra of **1** after immersion in mixtures of C_6H_6 and cyclohexene with varying compositions.

benzene
and
cyclohexane
3:7

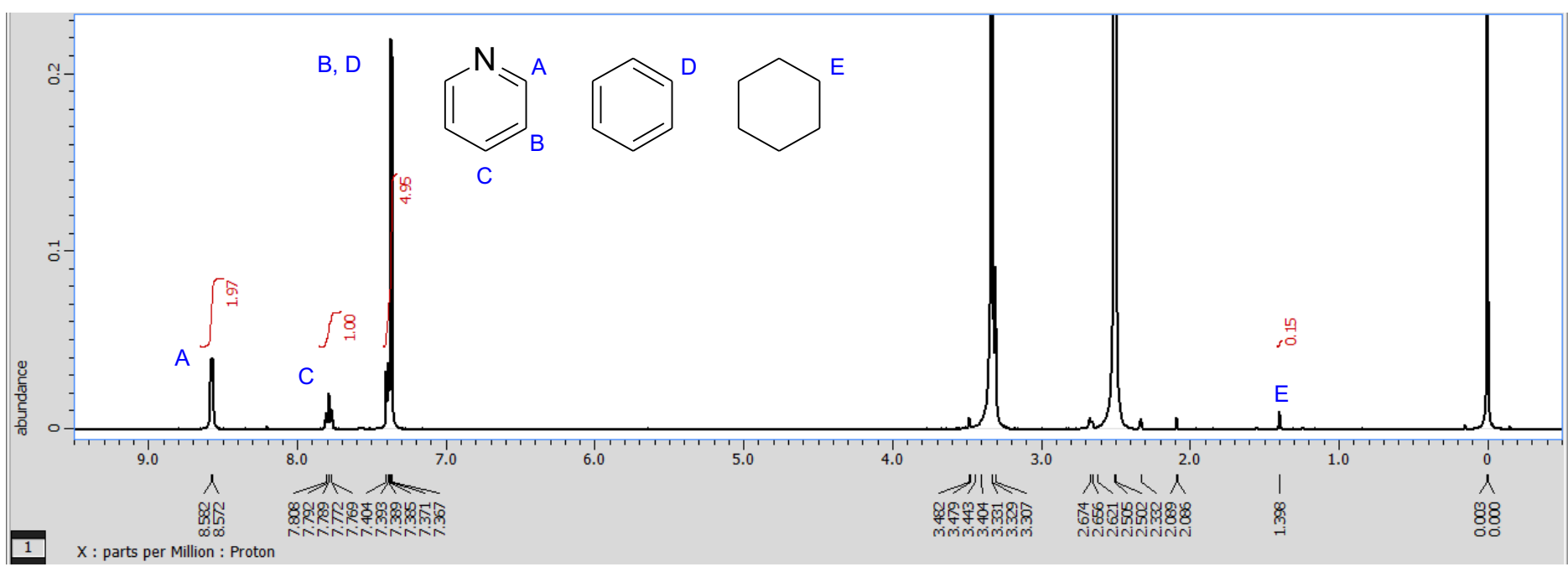

1:9

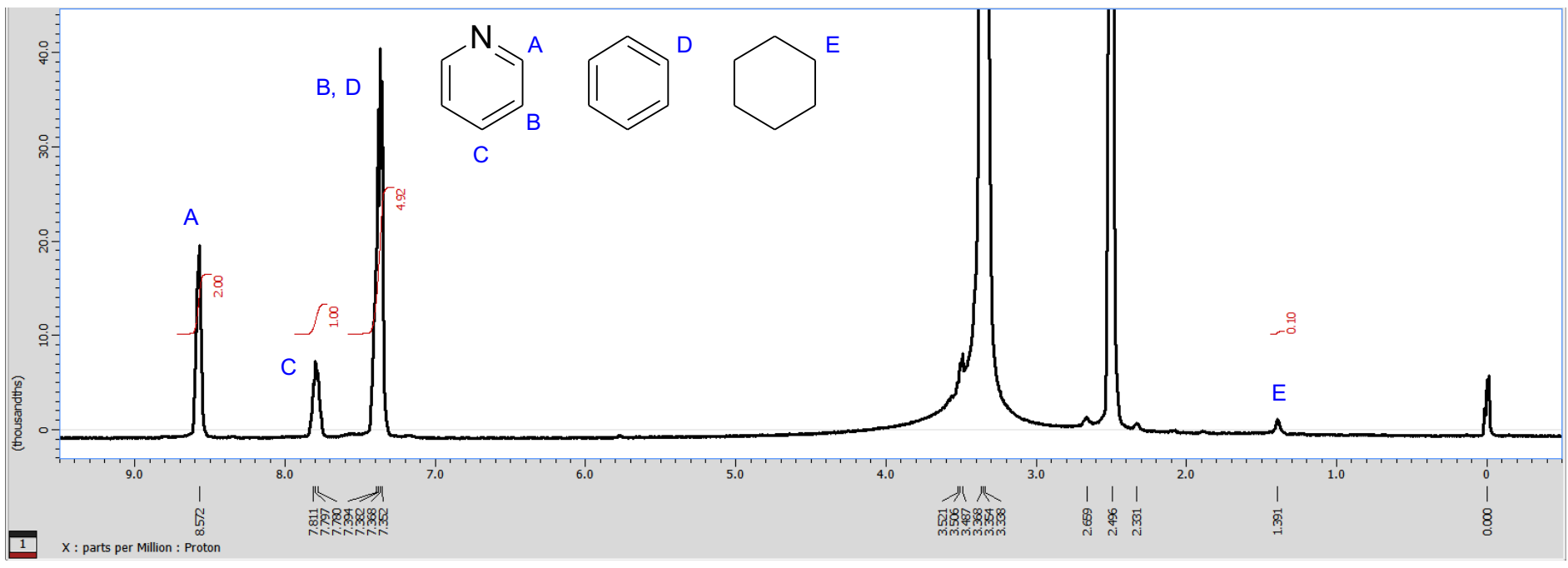

0:10

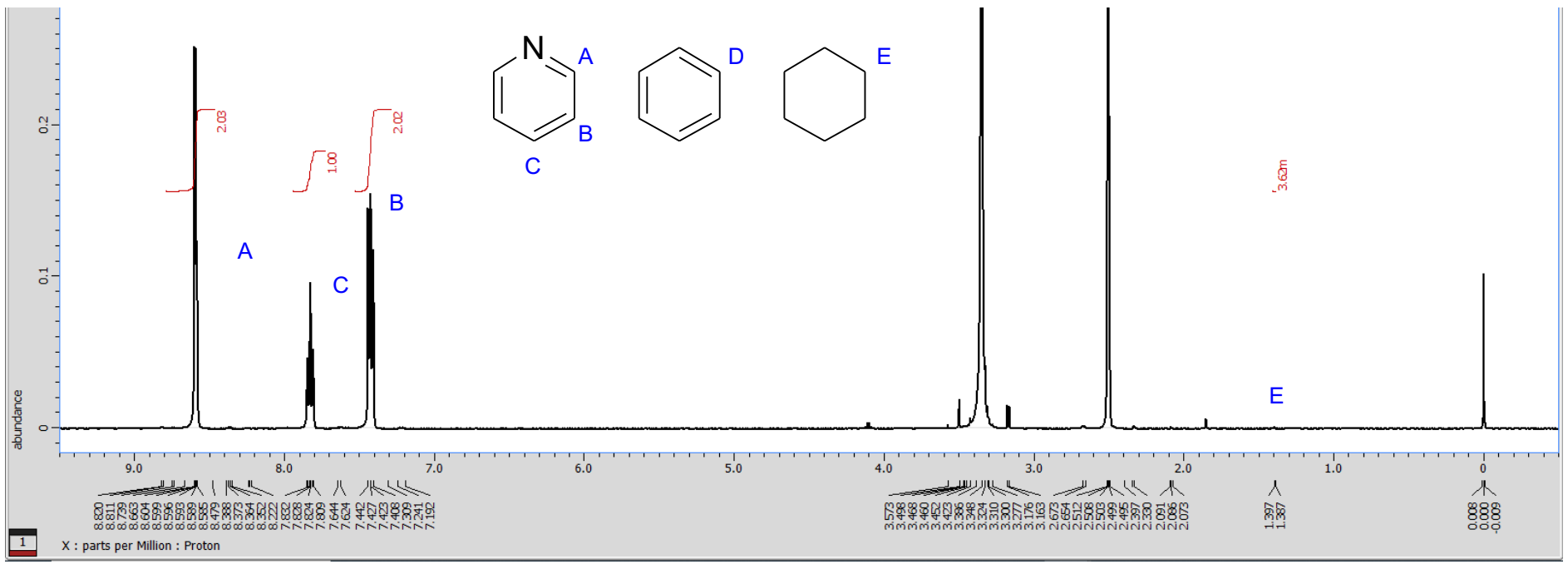

Figure S30. ^{1}H NMR spectra of **1** after immersion in mixtures of C_6H_6 and cyclohexane with varying compositions.

References

[S1] S. A. Bourne, J. Lu, A. Mondal, B. Moulton, M. J. Zaworotko, *Angew. Chem. Int. Ed.* **2001**, *40*, 2111-2113. https://doi.org/10.1002/1521-3773(20010601)40:11%3C2111::AID-ANIE2111%3E3.0.CO;2-F

[S2] NIST Computational Chemistry Comparison and Benchmark Database, NIST Standard Reference Database Number, 101, Release 22, May 2022, Editor: Russell D. Johnson III, http://cccbdb.nist.gov/

[S3] Z. Zhang, Y. Dai, S. Zhang, L. Chen, J. Gu, Y. Wang, W. Sun, *J. Energy Chem.*, **2025**, *101*, 278-297. https://doi.org/10.1016/j.jechem.2024.09.020

[S4] O. Shekhah, Y. Belmabkhout, Z. Chen, V. Guillerm, A. J. Cairns, K. Adil, M. Eddaoudi, *Nat. Commun.*, **2014**, *5*, 4228. https://doi.org/10.1038/ncomms5228

[S5] P. M. Bhatt, Y. Belmabkhout, A. Cadiau, K. Adil, O. Shekhah, A. Shkurenko, L. J. Barbour, M. Eddaoudi, *J. Am. Chem. Soc.*, **2016**, *138*, 9301–9307. https://doi.org/10.1021/jacs.6b05345
